# Transcriptome sequencing and analysis of the entomopathogenic fungus *Hirsutella sinensis* isolated from *Ophiocordyceps sinensis*

**DOI:** 10.1186/s12864-015-1269-y

**Published:** 2015-02-21

**Authors:** Zhi-Qiang Liu, Shan Lin, Peter James Baker, Ling-Fang Wu, Xiao-Rui Wang, Hui Wu, Feng Xu, Hong-Yan Wang, Mgavi Elombe Brathwaite, Yu-Guo Zheng

**Affiliations:** Institute of Bioengineering, Zhejiang University of Technology, Hangzhou, 310014 Zhejiang P R China; East China Pharmaceutical Group Limited Co., Ltd, Hangzhou, 311000 Zhejiang P R China; Polytechnic School of Engineering, New York University, 6 MetroTech Center, Brooklyn, NY 11201 USA

**Keywords:** *Ophiocordyceps sinensis*, *Hirsutella sinensis*, Transcriptome sequencing, Metabolic pathways, Gene differential expression

## Abstract

**Background:**

*Ophiocordyceps sinensis*, a worm and fungus combined mixture which *Hirsutella sinensis* is parasitic on the caterpillar body, has been used as a traditional medicine or healthy food in China for thousands of years. *H. sinensis* is reported as the only correct anamorph of *O. sinensis* and its main active ingredients are similar to the natural *O. sinensis*.

**Results:**

*H. sinensis* L0106, asexual strain of *O. sinensis*, was isolated and identified in this study. Three transcriptomes of *H. sinensis* at different cultivation periods (growth period 3d, pre-stable period 6d and stable period 9d) were sequenced for the first time by RNA-Seq method, and 25,511 unigenes (3d), 25,214 unigenes (6d) and 16,245 unigenes (9d) were assembled and obtained, respectively. These unigenes of the three samples were further assembled into 20,822 unigenes (All), and 62.3 percent of unigenes (All) could be annotated based on protein databases. Subsequently, the genes and enzymes involved in the biosynthesis of the active ingredients according to the sequencing and annotation results were predicted. Based on the predictions, we further investigated the interaction of different pathway networks and the corresponding enzymes. Furthermore, the differentially expressed genes (DEGs) of *H. sinensis* grown during different developmental stages (3d-VS-6d, 3d-VS-9d and 6d-VS-9d) were globally detected and analyzed based on the data from RNA-Seq, and 764 DEGs between 3d and 6d, 1,869 DEGs between 3d and 9d, and 770 DEGs between 6d and 9d were found, respectively.

**Conclusions:**

This work presented here would aid in understanding and carrying out future studies on the genetic basis of *H. sinensis* and contribute to the further artificial production and application of this organism. This study provided a substantial contribution and basis to further characterize the gene expression profiles of *H. sinensis* in the metabolic pathways of active ingredients.

**Electronic supplementary material:**

The online version of this article (doi:10.1186/s12864-015-1269-y) contains supplementary material, which is available to authorized users.

## Background

*Ophiocordyceps sinensiss*, a worm and fungus combined mixture which *Hirsutella sinensis* is parasitic on the caterpillar body [1], is found on the Tibetan plateau in the southwestern China [2], and widely used as one of important traditional Chinese medicines [3,4]. Modern pharmacological studies have proved that *H. sinensis* contains various active ingredients which have a broad therapeutic function [5,6]. *H. sinensis* attacks the caterpillar in the late autumn, and by the early summer of the following year, the caterpillar has been killed and the fruiting body of *H. sisnensis* protrudes from its head [6]. Recently, it is found that the *H. sinensis* has activities to modulate immune responses, inhibit tumour cell proliferation, enhance hepatic function, regulate insulin sensitivity and decrease plasma cholesterol levels [7-9]. As high demand for *O. sinensis* products grows and the supply of its wild type declines [10,11], mycelia of *H. sinensis* grown under artificial culture conditions are increasingly used in the traditional Chinese medicines. As the only correct anamorph of *O. sinensis* [12,13], *H. sinensis* could treat weakness after sickness, lung and kidney-associated diseases and sexual dysfunction [7]. It is reported that laboratory-grown *H. sinensis* mycelium has similar clinical functions and less associated toxicity compared to the wild *O. sinensis* [14]. So the fundamental research of the *H. sinensis* to investigate its function based on genetics is becoming more and more important and urgent.

Transcriptome could provide information of gene expression profiles and infer the gene functions, which has been widely applied to investigate the gene expression at RNA levels. The use of next-generation sequencing technology of transcriptome could systematically provide a complete view of expressed genes and their expression levels for the organisms at a given cultivation time, find genes and proteins involved in secondary metabolism and clarify functions of the corresponding metabolic pathways. Some methods such as serial analysis of gene expression (SAGE) [15], reassociation kinetics (Rot) [16], microarrays [17], sequencing of expressed sequence tags (ESTs) and full length transcripts [18] have been used to extensively study the transcriptomes. Gene and alternative isoform expression levels have been quantified using deep sequencing of RNAs by RNA-Seq [19,20]. RNA-Seq is more sensitive, both in terms of detection of lowly expressed and differentially expressed genes [21,22], and expression values from RNA-Seq correlate better with protein levels [23]. In RNA-Seq, all RNAs of a sample are randomly fragmented, reverse transcribed, ligated to adapters and then these fragments are sequenced. Gene expression levels can be estimated from the number of sequence reads deriving from each gene [24]. Expression estimates from RNA-Seq are quantitative over five orders of magnitude and replicates of mouse tissues are highly reproducible [19]. It was viable to directly analyze transcriptome of non-model organisms using RNA-Seq. Vera et al. [25] studied the transcriptome of butterflies by RNA-Seq at the condition of lacking species genetic information. RNA-Seq method can further be used to detect unknown genes, discover new transcripts, and accurately identify the variable shear loci and cSNP and UTR region [26]. Zhang et al. [27] used the paired-end RNA-Seq technology to sequence eight organs of cultivated rice, and detected 7,232 new transcription areas which have not yet been determined.

In recent years, due to the excessive excavation and high demand for *O. sinensis*, the supply of natural *O. sinensis* has been significantly reduced. Because of its important applications in the Chinese traditional medicines, it is very urgent to find new ways to meet the demand for *O. sinensis*. With the help of low-cost and fast sequencing technologies or approaches, the draft transcriptome sequence will facilitate understanding of the genetic basis of many traits at genome level and allow the undertaking of transcriptome-wide association studies of *H. sinensis,* which will provide theoretical foundation that *H. sinensis* is an alternative instead of the *O. sinensis* in the traditional Chinese medicines. Several features of *H. sinensis*, such as infection and low-temperature adaption, are investigated and confirmed based on its transcriptome and accordingly experiments. The availability of transcriptome will facilitate the development of new products and more efficient production of *H. sinensis*. This work presented here would aid in understanding active ingredients metabolic pathways and carrying out the future research of this organism on the genetic level. Moreover, the fruits from this study would further contribute to the use of *H. sinensis*, protect the wild *O. sinensis* and pave the foundations for developing new drugs in biopharmacologicals.

In this study, in order to protect the endangered wild *H. sinensis* resources, save biological information and investigate the important mechanisms of this traditional Chinese medicine, three transcriptomes of *H. sinensis* under different cultivation periods were sequenced and compared for the first time. 26,577,780 reads, 27,355,558 reads and 33,619,376 reads were obtained for 3d, 6d and 9d samples, respectively. After assembling, 25,511 unigenes, 25,214 unigenes and 16,245 unigenes were obtained for 3d, 6d and 9d samples, respectively. Compared with *O. sinensis* worm-part cDNA library which were clustered into 1,333 contigs and 4,172 unique sequences, as well as *O. sinensis* grass-part cDNA library which were clustered into 1,297 contigs and 3,805 unique sequences [28], *H. sinensis* has more abundant functional genes. Furthermore, we investigated and globally detected the differentially expressed genes of *H. sinensis* grown during different developmental stages based on RNA-Seq, and found 764 DEGs between 3d and 6d, including 549 and 215 genes up- and down-regulated from 3d to 6d (FDR ≤0.001), 1,869 DEGs between 3d and 9d, including 1,410 and 459 genes up- and down-regulated from 3d to 9d, and 770 DEGs between 6d and 9d, including 215 and 555 genes up- and down-regulated from 6d to 9d, respectively. Metabolic pathways of active ingredients including mannitol, cordycepin, purine nucleotides, pyrimidine nucleotides, unsaturated fatty acid, cordyceps polysaccharide and sphingolipid were further investigated based on the transcriptomes, as well as the transcriptome of genes involved in these metabolic pathways were predicted and verified according to the annotation information. The genes encoding exocellular hydrolytic enzymes such as protease, chitinase and lipase etc. which play important roles in the process of invading host, and the genes encoding low-temperature enzymes such as malate dehydrogenase, ethanol dehydrogenase and chitinase etc., were also predicted and cloned. The infection and cold tolerance mechanisms of *H. sinensis* were further investigated and discussed.

## Results

### Summary of RNA-Seq data sets

To obtain an overview of the *H. sinensis* transcriptome at different developmental stages, the RNA samples were prepared from the mycelium in different cultivation phases (3d, 6d and 9d), and poly(A)-enriched mRNA samples were subjected to high-throughput Illumina GA IIx sequencing. We totally obtained 26,577,780 reads and 27,355,558 reads both with an average length of 90 nt and 33,619,376 reads with an average length of 75 nt for the samples of 3d, 6d and 9d, respectively. These reads were assembled with short reads assembling program SOAPdenovo, resulting in 165,480 contigs for 3d, 142,072 contigs for 6d and 44,112 contigs for 9d (Table 1). The mean contig size was 178 nt (3d) with lengths ranging from 50 nt to 6,984 nt, 191 nt (6d) with lengths ranging from 50 nt to 6,863 nt and 386 nt (9d) with lengths ranging from 75 nt to 6,037 nt. SOAPdenovo connected the contigs using N to represent unknown sequences between each two contigs, and then 92,721 scaffolds were made, with mean sizes of 524 nt (3d), 520 nt (6d) and 776 nt (9d) (Table 1). With paried-end reads, the 35,516 scaffolds (3d) generated 25,511 unigenes (mean size: 681 nt), the 35,448 scaffolds (6d) generated 25,214 unigenes (mean size: 682 nt) and the 21,757 scaffolds (9d) generated 16,245 unigenes (mean size: 994 nt) (Table 1). Unigenes from each sample (3d, 6d and 9d) were taken into further process of sequence splicing and redundancy removing with sequence clustering software to acquire non-redundant unigenes of maximal length. At last, 20,822 unigenes (All) were obtained with a mean size of 1,013 nt. To demonstrate the quality of sequencing data, we randomly selected 133 unigenes (All) and accordingly designed primers for RT-PCR amplification. In this study, all primer pairs resulted in a band of the expected size and the identity of all PCR products were confirmed by Sanger sequencing, which indicated that the transcriptome quality of *H. sinensis* was good enough for further analysis.

**Table 1:** **Output statistics of sequencing and assembly**

**Samples**	**3d**	**6d**	**9d**	**All**
Reads				
Number of reads	26,577,780	27,355,558	33,619,376	
Read size (nt/read)	90	90	75	
Total nucleotides (nt)	2,392,000,200	2,462,000,220	2,521,453,200	
Q20 percentage (%)	89.46	89.66	86.08	
N percentage (%)	0.01	0.00	0.00	
GC percentage (%)	58.19	57.92	57.00	
Contigs				
Number of contigs	165,480	142,072	44,112	
Mean size of contigs	178	191	386	
Length of all contigs (nt)	29,423,650	27,169,112	17,023,492	
Scaffolds				
Number of scaffolds	35,516	35,448	21,757	
Mean size of scaffolds	524	520	776	
Length of all scaffolds (nt)	18,593,527	18,426,448	16,891,810	
Unigenes				
Number of unigenes	25,511	25,214	16,245	20,822
Mean size of unigenes	681	682	994	1,013
Length of all unigenes	17,383,818	17,203,586	16,152,293	21,085,467

### Functional annotation

**Figure 1:**
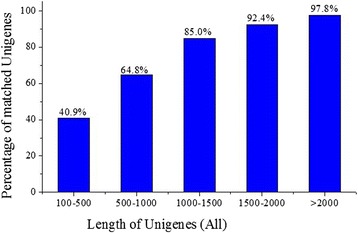
**Proportion of sequences with matches in protein databases.** Effects of unigene length on the percentage of sequences for which significant matches were found. The proportion of sequences with matches in protein databases is greater among the longer unigene sequences.

**Figure 2:**
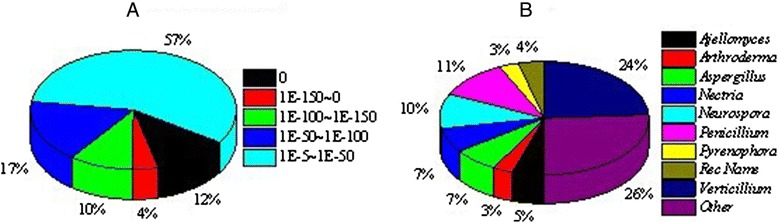
**Characteristics of homology search of uinigenes against the nr database. (A)** E-value distribution of the top hits for uinigenes with a cut-off E-value of 1.0e-5. **(B)** Species distribution is shown as a percentage of the total homologous uinigenes with an E-value of at least 1.0e-5.

The reads of *H. sinensis* in different cultivation periods (3d, 6d and 9d) were assembled, and then 25,511 unigenes (3d), 25,214 unigenes (6d) and 16,245 unigenes (9d) were obtained, respectively. Furthermore, these unigenes were spliced and 20,822 unigenes (All) were generated (Additional file 1: Table S1). Finally, the unigenes were carried out for COG functional annotation. For protein functional annotation, the unigene sequences were searched using BLASTx against the protein databases including non-redundant (nr), Swiss-Prot, KEGG and COG using a cut-off E-value of 1.0e-5. If the results of different databases conflict with each other, a priority order of nr, Swiss-Prot, KEGG and COG should be followed. Using this approach, 12,980 unigenes returned an above cut-off BLAST result (nr, 12,790 genes; Swiss-Prot, 172 genes; KEGG, 14 genes; COG: 4 genes). Figure 1 indicates that the proportion of sequences with matches in protein databases is greater among the longer unigene sequences. Specifically, a 97.8% of match efficiency was observed for sequences longer than 2,000 bp, whereas the match efficiency decreased to about 64.8% for those ranging from 500 to 1,000 bp and to 40.9% for sequences between 100 to 500 bp (Figure 1). The E-value distribution of the top hits in the nr database (12,790 unigenes) showed that 43% of the mapped sequences have strong homology (smaller than 1.0e-50), whereas 57% of the homology sequences ranged between 1.0e-5 to 1.0e-50 (Figure 2A). For species distribution, 24% of the unigenes (hit in nr database) trained with sequences from the *Verticillium alboatrum* (Figure 2B), followed by the *Penicillium chrysogenum* (11%) and *Neurospora crassa* (10%).

### GO and COG classification

**Figure 3:**
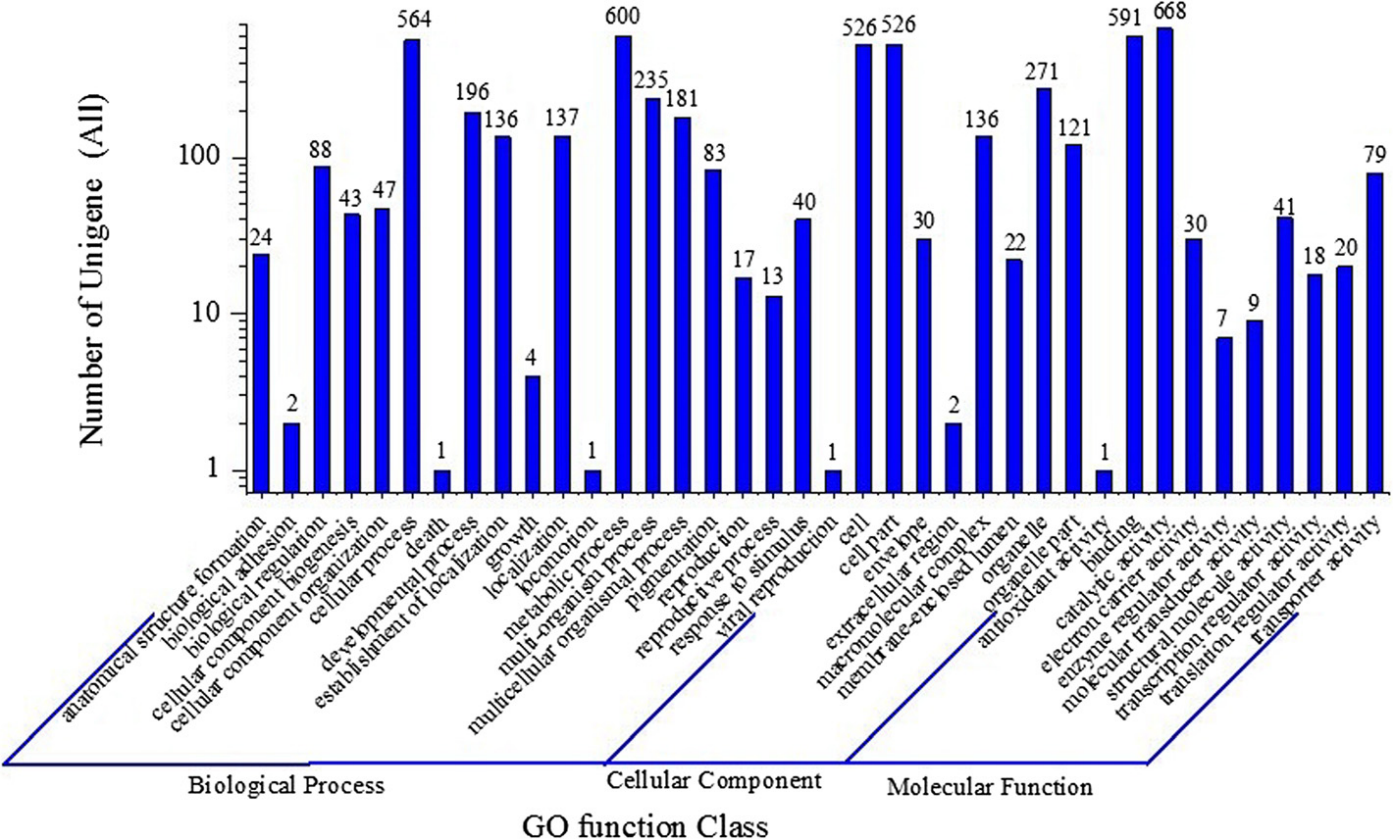
**Histogram presentation of Gene Ontology classification.** GO has three ontologies: molecular function, cellular component and biological process. It indicates the number of unigenes in a category. The basic unit of GO is GO-term, and every GO-term belongs to a type of ontology. GO functional analysis provided GO functional classification annotation for DEGs as well as GO functional enrichment analysis for DEGs.

**Figure 4:**
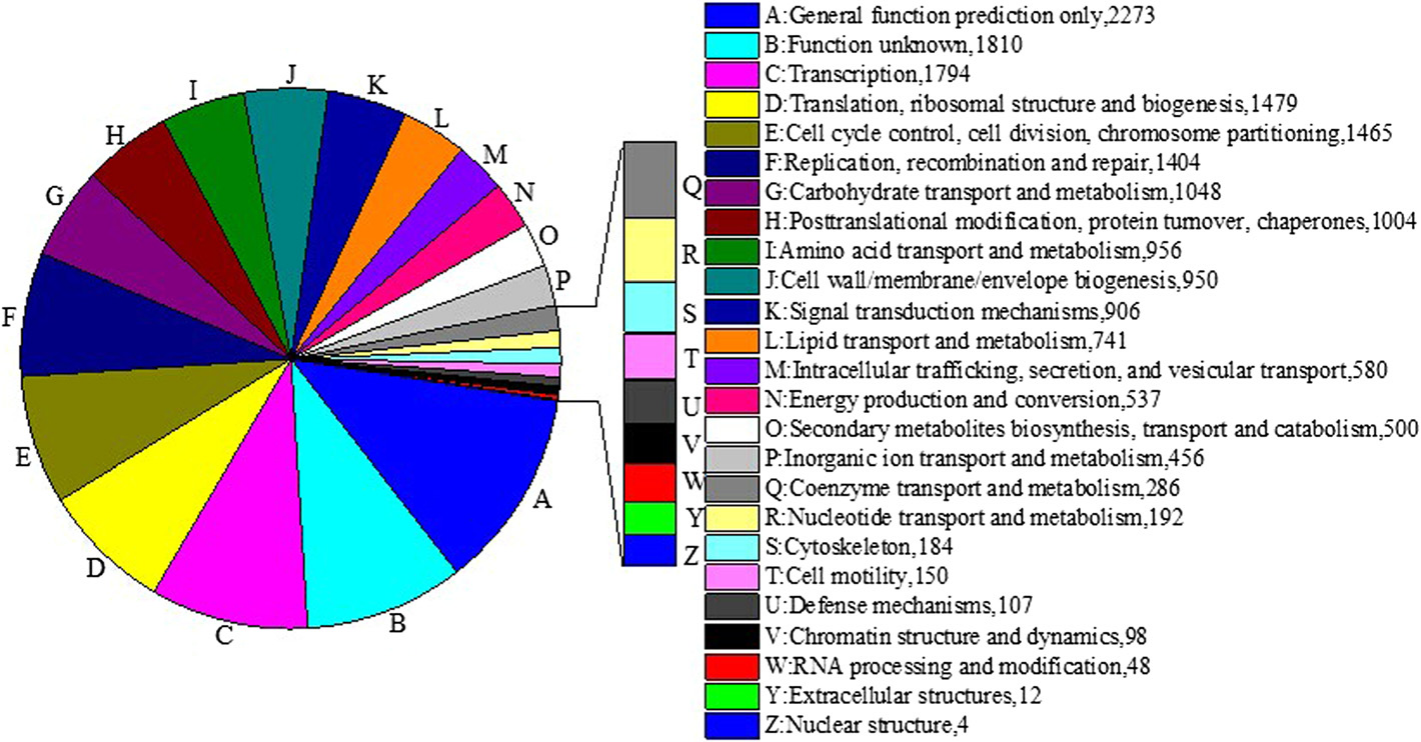
**Cluster of orthologous groups (COG) function classification of unigene sequence.**

GO assignments were used to classify the unigene functions of *H. sinensis*. Based on sequence homology, 1,353 unigenes were categorized into 38 functional groups (Figure 3). In terms of the biological process, unigenes involved in ‘cellular process’ (564 members), ‘metabolic process’ (600 members) and ‘multi-organism process’ (235 members) accounted for the majority, while unigenes involved in ‘biological adhesion’ (2 members), ‘cell death’ (1 member), ‘growth’ (4 members), ‘locomotion’ (1 member) and ‘viral reproduction’ (1 member) accounted for a minority. For cellular component, unigenes involved in ‘cell’ (526 members), ‘cell part’ (526 members) and ‘organelle’ (271 members) accounted for the majority, while unigenes involved in ‘extra cellular region’ (2 members) and ‘membrane-enclosed lumen’ (22 members) accounted for a minority. When the molecular function was investigated, it is found that unigenes involved in ‘binding’ (591 members) and ‘catalytic activity’ (668 members) accounted for the majority, while unigenes involved in ‘antioxidant activity’ (1 member), ‘enzyme regulator activity’ (7 members) and ‘molecular transducer activity’ (9 members) accounted for a minority (Figure 3). To further evaluate the completeness of transcriptome and the effectiveness of annotation process, the annotated sequences were screened for the genes involved in COG classifications. In total, out of 12790 nr hits, 6,353 sequences have COG classifications (Figure 4). Among the 25 COG categories, the cluster for ‘general function prediction’ represents the largest group (2,273 members) followed by ‘function unknown’ (1,810 members) and ‘transcription’ (1,794 members). The following categories: Nuclear structure (4 members); Extracellular structures (12 members) and RNA processing and modification (48 members), represent the small groups. To identify the biological pathways that are active in the *H. sinensis*, we mapped the 12,980 annotated unigenes to the reference canonical pathways in KEGG, there are totally 8,724 sequences can be assigned to 159 KEGG pathways. The most representative pathways by the unigenes were ‘metabolic pathways’ (3,500 members), ‘starch and sucrose metabolism’ (1,344 members) and ‘biosynthesis of secondary metabolites’ (1,196 members). These annotations provide a valuable resource for investigating specific processes, functions and pathways in *H. sinensis*. COG function classification of *H. sinensis* was also compared with *O. sinensis* grass-part (OSGP) and worm-part (OSWP). The results showed that *H. sinensi* has more unigenes than both OSGP library and OSWP library in each of the COG categories indicating *H. sinensis* transcriptome has a more active expression than both OSGP and OSWP (Additional file 2: Table S2).

### Reference genome analysis of the *H. sinensis* transcriptome

**Figure 5:**
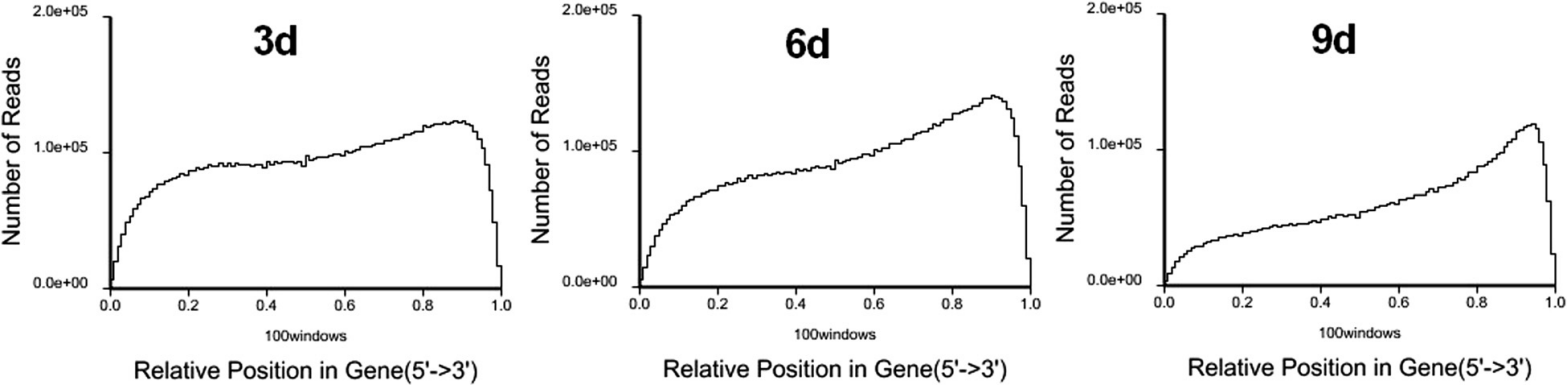
**Distribution statistics of** ***H. sinensis*** **transcriptome reads mapped to reference gene.** The randomness of 3d transcriptome is the best, followed with 6d and 9d.

**Figure 6:**
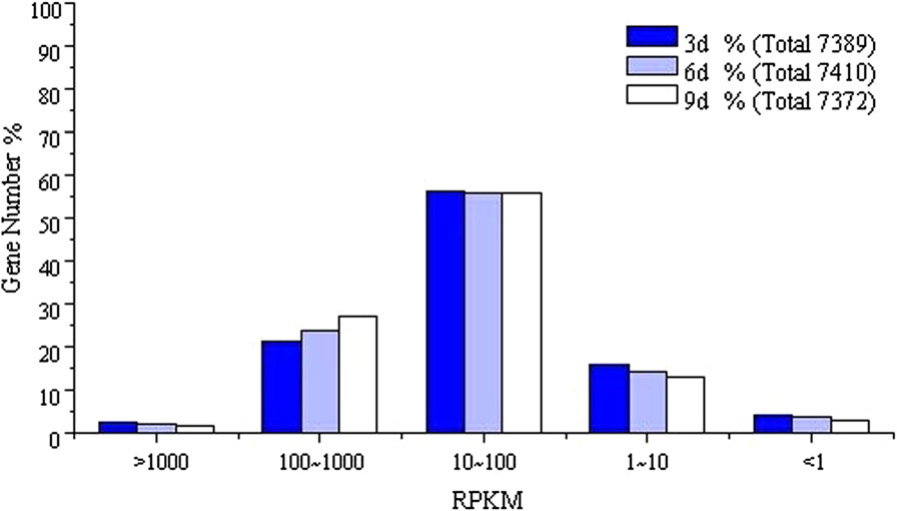
**Distribution statistics of** ***H. sinensis*** **transcriptome genes expression levels.**

The comparison and analysis between reference genome and sequenced transcriptome could clarify their intrinsic link in structure, function and evolution of species, and further discover their common features and uniqueness. The transcriptome against reference genome analysis revealed an extensive expression of the whole *H. sinensis* genome (the genome of the *H. sinensis* L0106 with 102.7 Mb in our laboratory had sequenced, data not shown). Of the transcriptome reads, about 71.30% (3d), 70.85% (6d) and 78.21% (9d) was mapped to the reference genome of *H. sinensis.* Moreover, 33.00% (3d), 31.55% (6d) and 32.92% (9d) could be mapped to reference genes (Additional file 3: Table S3). There were 13,031,334 perfect matched reads and accounted for 49.03% for 3d samples, 19,380,240 perfect matched reads and accounted for 70.85% for 6d samples, and 10,185,645 perfect matched reads and accounted for 60.59% for 9d samples, respectively. The mapped reads were classified by both of mismatch number and uniqueness of alignment position. There were 17,285,218 unique matched reads and 1,664,653 multi-position matched reads for 3d samples, 17,445,855 unique matched reads and 1,934,385 multi-position matched reads for 6d samples, 12,094,781 unique matched reads and 1,052,137 multi-position matched reads for 9d samples, respectively. In order to assess the randomness of RNA-Seq, the randomness of mRNA fragmentation was evaluated with the reads distribution in reference genes. The total number of reads aligned to reference genes was counted, and the reads were located in relative position in reference gene (Figure 5). More than 80% reads of 3d, 6d and 9d were located in relative position from 0.2 to 0.9, the reads number of 3d in this region distributed random and was around 1.0e + 05, the reads number of 6d with the relative position at 0.2 was around 0.5e + 05 but the relative position at 0.9 was around 1.5e + 05, and the reads number of 9d with the relative position at 0.2 was around 0.3e + 05 but the relative position at 0.9 was around 1.3e + 05, which indicated that the distribution quality of reads of 3d was better than both 6d and 9d. Therefore, the distribution of reads in the reference genes was homogeneous and the randomness of fragmentation was good, it indicated that fragmentation of mRNA was performed well and conducive to transcriptome analysis. Gene coverage is the percentage of a gene covered by reads, this value equals to ratio of the number of bases in a gene covered by unique mapping reads to number of total bases in the gene. We found that 7,496 genes were covered by the reads of 3d, 6d and 9d transcriptomes, and the genes with more than 70% of the gene coverage were over 90%. Number of RPKM (defined in Method) was calculated to quantify the overall transcriptional activity of the genes (Figure 6). The results showed that RNA-Seq data obtained in this study was more sensitive, which displayed a comprehensive landscape of the *H. sinensis* transcriptome.

### Prediction and optimization of novel transcripts

**Figure 7:**
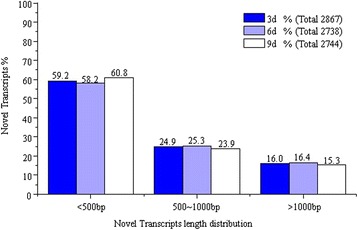
**Predication of** ***H. sinensis*** **novel transcript units.**

**Figure 8:**
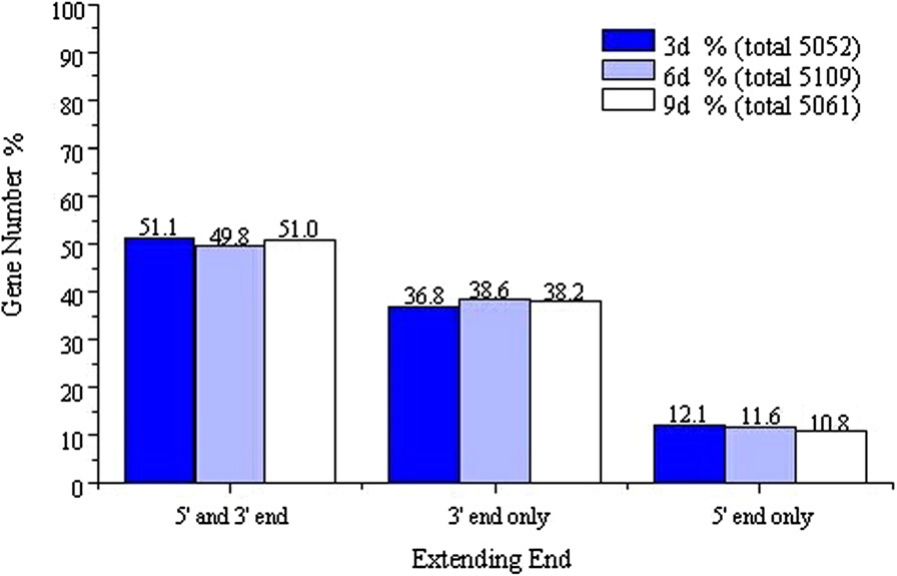
**Refinement of** ***H. sinensis*** **transcript gene structures.**

Novel transcripts can be found by high throughput sequencing since present databases may be incomplete. Gene models with a length more than 150 bp found in intergenic regions (200 bp away from upstream or downstream genes) were thought to be candidates of the novel transcripts. Extensive reads mapping and clustering revealed that 2,867 (3d), 2,738 (6d) and 2,744 (9d) novel transcripts with the significant expression levels were above the surrounding intergenic region, of which 40.84% (3d), 41.78% (6d) and 39.18% (9d) were longer than 500 bp and provided a sufficient candidate number of novel transcripts (Figure 7), and most of these novel transcripts belong to the non-coding RNA. We also globally mapped the 5′- and 3′-boundaries of *H. sinensis* genes by searching for a sharp reduction of RNA-Seq reads signals at both ends of annotated genes. Genes whose 5′- or 3′-boundaries overlap with other genes were excluded from the analysis. The results defined or extended 5′- or (and) 3′-boundary regions for 5,052 transcribed genes in *H. sinensis* transcriptome of 3d, 5,109 transcribed genes in *H. sinensis* transcriptome of 6d, and 5,061 transcribed genes in *H. sinensis* transcriptome of 9d (Figure 8).

### Alternative splicing in *H. sinensis*

**Figure 9:**
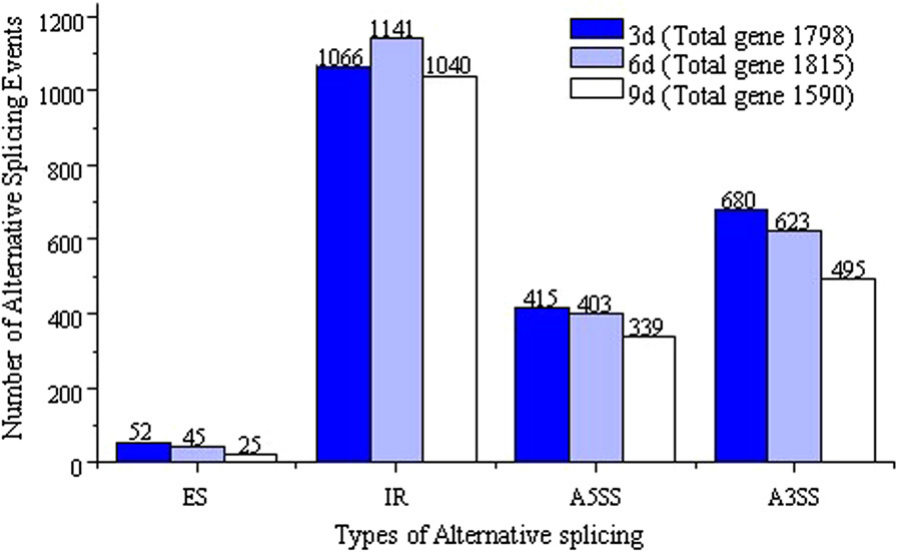
**AS events in** ***H. sinensis*** **transcriptome.**

Alternative splicing (AS) is a mechanism brings remarkable diversity to proteins which make it possible for a gene to generate different mRNA transcripts and then translate into distinguishable proteins [29-31]. Though alternative splicing is known to be universal in eukaryotes, we may underestimate the number of genes that undergo alternative splicing. Recently, many new alternative splicing events were discovered in human [20,32,33], mouse [19,34] and Arabidopsis [35]. Alternative splicing events mostly occur on the genes which involved in signal transduction and expression regulation, mechanisms of cell differentiation and apoptosis could be clarified by studying these alternative splicing events. To assess the genome-wide extent of AS events in *H. sinensis*, with different developmental stages (3d, 6d and 9d), we performed computational analysis to determine the known and putative splicing junctions and then to identify sequence reads mapping to these regions using stringent criteria. 5,203 genes of *H. sinensis* underwent AS with 6,324 AS events (Figure 9), 1,798 genes of 3d samples underwent AS with 2,213 AS events, 1,813 genes of 6d samples underwent AS with 2,212 AS events, and 1,590 genes of 9d samples underwent AS with 1,899 AS events, respectively in four common types of AS events, including Exon skipping (ES), Intron retention (IR), Alternative 5′ splice site (A5SS) and Alternative 3′ splice site (A3SS). For the data obtained in this study, 24.33% (3d), 24.49% (6d) and 21.57% (9d) of *H. sinensis* genes were estimated to undergo AS.

In this study, *Ustilago maydis* [36] and *Magnaporthe grisea* [37], two important fungal pathogens to plant, shared similar infection process with *H. sinensis*, are chosen as references to develop transcriptome analysis. The IR in *H. sinensis* is the predominant form of AS, accounting for 59.29% (3d), 62.87% (6d) and 65.41% (9d) of all AS isoforms, indicating *H. sinensis* has a richer gene activity compared with *U. maydis* and *M. grisea*.

### Gene expression difference analysis

Studies on the up- and down-regulated DEGs of *H. sinensis* were carried out by GO annotation, the results were shown in Table 2. The number of up- and down-regulated DEGs were further compared among 3d-VS-6d, 9d-VS-3d and 9d-VS-6d by GO component ontology, GO function ontology and GO process ontology and the details were shown in Table 3, Table 4 and Table 5, respectively. There were 467 DEGs and 255 up-regulated genes in GO component ontology, 1,040 DEGs and 423 up-regulated genes in GO function ontology, and 568 DEGs and 240 up-regulated genes in GO process ontology. A large number of differentially expressed genes were up-regulated in GO function ontology, especially in terms of ‘catalytic activity’, ‘oxidoreductase activity’ and ‘hydrolase activity’, which indicated *H. sinensis* could secrete large amounts of enzymes involved in secondary metabolism pathway, infection mechanism and cold tolerance mechanism. 36 down-regulated genes (3d-VS-6d), 63 up-regulated genes (9d-VS-3d), and 13 up-regulated genes (9d-VS-6d) were involved in ribonucleoprotein complex by GO component ontology, indicating that the speed and efficiency of protein synthesis in stable period were higher than growth period. 36 down-regulated genes (3d-VS-6d), 63 up-regulated genes (9d-VS-3d), and 12 up-regulated genes (9d-VS-6d) were involved in structural molecule activity by GO function ontology, showing that structural molecule in stable period was more stable than growth period. 35 down-regulated genes (3d-VS-6d), 58 down-regulated genes (9d-VS-3d), and 27 down-regulated genes (9d-VS-6d) were involved in metabolic process by GO process ontology, which demonstrated that genes involved in metabolic processes were more abundant in stable period. Subsequently, ExtendGene, Exon skipping and Intron retention analysis of 3d, 6d and 9d were absolutely compared, respectively. There were common 4,177 ExtendGenes, 10 skipping Exons and 460 retentional Introns among 3d, 6d and 9d. Comparisons among of alternative 5′ splice site, alternative 3′ splice site and the number of transcripts analysis of 3d, 6d and 9d showed that there were common 131 alternative 5′ splice sites, 173 alternative 3′ splice sites and 7,239 transcripts among 3d, 6d and 9d. Finally, the comparison of differential expression genes, up- and down-regulated genes analysis of 3d-VS-6d, 9d-VS-3d and 9d-VS-6d were carried out. There were common 113 differential expression genes, while no common up- and down-regulated genes among 3d-VS-6d, 9d-VS-3d and 9d-VS-6d (Additional file 4: Figure S1).

**Table 2:** **Statistic chart of** ***H. sinensis*** **DEGs carried out by GO annotation**

**GO annotation**	**Up-regulated DEGs**	**Down-regulated DEGs**	**Component ontology**	**Function ontology**
3d-VS-6d	203	91	117	259
9d-VS-3d	249	435	279	557
9d-VS-6d	117	158	96	235

**Table 3:** **Statistics chart of up- and down-regulated** ***H. sinensis*** **DEGs by GO component ontology**

**GO component ontology**	**3d-VS-6d**		**9d-VS-3d**		**9d-VS-6d**	
	**Up**	**Down**	**Up**	**Down**	**Up**	**Down**
Ribonucleoprotein complex	0	36	63	1	13	0
Intrinsic to membrane	23	5	10	32	6	10
Intracellular membrane-bounded organelle	11	4	25	22	9	8
Membrane	7	2	4	16	3	7
Cell part	8	0	7	22	2	9
Intracellular part	5	3	24	13	9	3
Organelle inner membrane	3	2	5	5	2	1
Intracellular	2	1	1	4	0	1
Protein complex	1	2	8	2	4	1

**Table 4:** **Statistics chart of up- and down-regulated** ***H. sinensis*** **DEGs by GO function ontology**

**GO function ontology**	**3d-VS-6d**		**9d-VS-3d**		**9d-VS-6d**	
	**Up**	**Down**	**Up**	**Down**	**Up**	**Down**
Structural molecule activity	0	36	63	0	12	0
Catalytic activity	22	5	10	55	7	24
Oxidoreductase activity	19	6	17	27	15	13
Hydrolase activity	20	4	4	24	6	6
Binding	16	6	11	44	5	17
Transition metal ion binding	17	0	7	29	2	8
Metal ion binding	11	3	10	20	4	9
Transferase activity	12	2	10	31	7	12
Iron ion binding	12	1	1	14	3	6
Nucleic acid binding	8	4	22	20	5	4
Coenzyme binding	7	2	3	9	1	2
Cation binding	3	5	10	6	4	2
Nucleoside-triphosphatase activity	6	2	5	14	4	4
Lyase activity	3	2	1	3	1	2
Kinase activity	4	1	9	4	4	2
Structural molecule activity	0	36	63	0	12	0

**Table 5:** **Statistics chart of up- and down-regulated** ***H. sinensis*** **DEGs by GO process ontology**

**GO Process ontology**	**3d-VS-6d**		**9d-VS-3d**		**9d-VS-6d**	
	**Up**	**Down**	**Up**	**Down**	**Up**	**Down**
Metabolic process	6	35	8	58	10	27
Gene expression	0	34	64	2	14	2
Transport	19	3	9	31	7	13
Transcription	14	1	2	25	0	9
Establishment of localization	6	1	6	8	3	1
Carboxylic acid metabolic process	6	0	0	11	0	5
Protein modification process	2	1	3	12	2	1
Carboxylic acid metabolic process	6	0	0	11	0	5
Translation	0	3	8	3	0	2
Response to stimulus	1	3	9	1	3	0
Cellular metabolic process	2	1	3	4	2	1
Glucose catabolic process	0	0	8	0	5	0
Primary metabolic process	0	0	2	6	3	3
DNA metabolic process	0	0	5	2	2	3

### Functional analysis of differential gene expression based on RNA-Seq data

**Figure 10:**
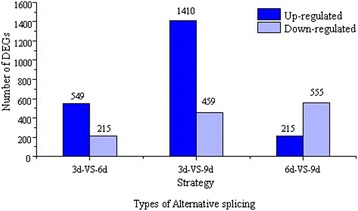
**Statistics of DEGs from** ***H. sinensis*** **between different developmental stages.**

So far, the molecular mechanisms resulting in various kinds of functional complexity in *H. sinensis* mycelium have not been illuminated [38,39]. To understand this molecular mechanisms, the DEGs of *H. sinensis* grown during different developmental stages (3d-VS-6d, 3d-VS-9d and 6d-VS-9d) were also used to investigate the gene functions based on the differential gene expression level in different culture times. We found 764 DEGs between 3d and 6d, including 549 and 215 genes up- and down-regulated from 3d to 6d (FDR ≤0.001), 1,869 DEGs between 3d and 9d, including 1,410 and 459 genes up- and down-regulated from 3d to 9d, and 770 DEGs between 6d and 9d, including 215 and 555 genes up- and down-regulated from 6d to 9d, respectively (Figure 10). GO functional enrichment analysis revealed that genes up-regulated during the developmental stages of *H. sinensis* were mainly involved in ‘structural molecule activity’, ‘ribonucleoprotein complex’, ‘macromolecular complex’, ‘gene expression’ and ‘intrinsic to membrane’ (Corrected *p*-value ≤ 0.05) (Additional file 4: Figure S2), suggesting these up-regulated genes played important roles and promoted the formation of cell structures in the process of mycelia reproduction. In addition, the KEGG metabolic pathway analysis indicated that the genes up-regulated from 3d to 6d were specifically located in the pathways of ‘ribosome’, ‘caprolactam degradation’, ‘metabolic pathways’, ‘nitrogen metabolism’ and ‘fatty acid metabolism’, while genes up-regulated from 6d to 9d were mainly associated with ‘ribosome’, ‘metabolic pathways’, ‘phenylalanine, tyrosine and tryptophan biosynthesis’ and ‘biosynthesis of secondary metabolites’ (Additional file 4: Figure S3). The results indicated that these up-regulated genes in such life stage mainly promoted secondary metabolism and biosynthesis of active ingredients, which was consistent with the description of the *H. sinensis* life cycle (Additional file 4: Figure S4). Therefore, these analyses indicated that *H. sinensis* drastically altered the manner of gene expression during the developmental stages to produce numerous functional components.

### Secondary metabolism analysis and verification

According to the metabolic pathways from annotated KEGG analysis based on the transcriptome, 7 main metabolic pathways of active ingredients including mannitol, cordycepin, purine nucleotides, pyrimidine nucleotides, unsaturated fatty acid, cordyceps polysaccharide and sphingolipid in *H. sinensis* were predicted, and the genes involved in these pathways were further cloned and expressed to verify the predictions. Using the glycolytic pathway (map00010) and fructose-mannose pathway (map00051) as references, mannitol metabolic pathway of *H. sinensis* was predicted (Additional file 4: Figure S5). However, the mannitol-1-phosphatase converting mannitol-1-P into mannitol is not found, which indicates that other unknown phosphatases take place the role of mannitol-1-phosphate to generate mannitol. Subsequently, 6 hexokinase genes, 3 glucose phosphate isomerise genes and 1 mannitol-1-P dehydrogenase gene involved in mannitol metabolic pathway were successfully cloned (Additional file 4: Figure S6), and corresponding proteins which expressed in *E. coli* BL21 were detected by Sodium Dodecyl Sulfate Polyacrylamide Gel Electrophoresis (SDS-PAGE) (Additional file 4: Figure S7). In the KEGG, the biosynthesis of adenosine (map00230) has been confirmed. To date, there were still no literatures about the biosynthesis of cordycepin from adenosine [40]. It is found that in the pathway of cordycepin of *H. sinensis* there is special simple sugars which was called cordycepose, we speculated that cordycepose and adenosine exchanged the glycosylation by the function of N-glycosylation lyase, and then generated cordycepin (Additional file 4: Figure S8). The genes involved in cordycepin metabolic pathway were further verified by PCR, 1 ADP-ribose pyrophosphatase gene, 2 ribose-phosphate pyrophosphokinase genes, 2 5′-nucleotidase genes and 1 N-glycosylase gene etc. were successfully cloned (Additional file 4: Figure S9), and corresponding proteins which expressed in *E. coli* BL21 were also detected by SDS-PAGE (Additional file 4: Figure S10). Furthermore, purine biosynthesis pathway of *H. sinensis* was predicted according to the previously reported purine metabolic pathway (map00230). It is found that purine metabolic pathway starts from adenosine and ends with urate after 7 steps of catalysis (Additional file 4: Figure S11), and genes which encoding purine nucleosidase and adenosine kinase etc. were cloned (Additional file 4: Figure S12), and then the corresponding proteins were successfully expressed (Additional file 4: Figure S13). For the pyrimidine metabolism pathway (map00240) based on KEGG, the biosynthesis pathway which starts from L-glutamine and ends with uridylic acid, cytidylic acid, thymidylic acid etc. in *H. sinensis* was analyzed and verified (Additional file 4: Figure S14), uridine triphosphate, cytidine triphosphate and cytidine diphosphate were intermediate products in this pathway. Then the biosynthesis pathways which starts from acetyl-CoA and hexadecanoate and ends with docosahexaenoic acid and butyryl-[acp] were obtained in the summary of the KEGG fatty acid biosynthesis pathway (map00061) and unsaturated fatty acids biosynthesis pathway (map01040) (Additional file 4: Figure S15), hexadecanoate was generated by fatty acid biosynthesis pathway and then used as a starting material to promote the unsaturated fatty acids biosynthesis. Moreover, based on the fructose and mannose metabolism pathway (map00051), galactose metabolism pathway (map00052), N-glycan biosynthesis pathway (map00510) and sphingolipid metabolism pathway (map00600) from the KEGG, the biosynthesis pathway of cordyceps polysaccharide which starts from D-glucose and D-galactose and ends with UDP-glucose and (GlacNAc)2(Man)5(Asn)1 (Additional file 4: Figure S16), as well as the sphingolipid biosynthesis pathway which starts from palmitoyl CoA andends with sphingosine (Additional file 4: Figure S17), could be predicted and verified in the same manner as described above in this study.

## Discussion

The use of *O. sinensis* has a long history both in traditional Chinese medicine and Tibetan medicine in China. The huge market demand has led to over harvesting and severe devastation of fragile alpine environments, however, *O. sinensis* grows slowly on artificial media and attempts at solid cultivating the fungus to produce fruiting bodies have consistently failed [40]. To meet the requirement of *O. sinensis* from the market, mycelia of *H. sinensis* grown under artificial liquid cultivation are increasingly used as an alternative in the medicinal products. As the anamorph of *O. sinensis* [12,13], *H. sinensis* has activities to modulate immune responses, inhibit tumour cell proliferation, enhance hepatic function, regulate insulin sensitivity and decrease plasma cholesterol levels, this makes it become more and more valuable in pharmacology. Studies have demonstrated the diverse bioactive ingredients and broad medical effects of *H. sinensis*, but there were little reports which demonstrated genetic information of *H. sinensis*. Therefore, the fundamental research of the *H. sinensis* to investigate and preserve its genetic information based on RNA-Seq is becoming more and more important and urgent.

Next generation sequencing has opened the door to transcriptome analysis of non-model organisms [41]. The growing number of species for which significant genetic resources are available is sparking a new era of study in which fundamental genetic questions underlying phenotypic evolution, adaptation and speciation can be addressed [41]. In this study, the next-generation sequencing technology was applied to *H. sinensis* transcriptome sequencing, and the unigenes generated from transcriptomes under different culture periods were systematically investigated. To our knowledge, this is first de novo assembly of the *H. sinensis* transcriptome from a short read sequencing platform and the results are presented that allows high throughput analysis and further research for comparative transcriptomics. Being an insect pathogen, *H. sinensis* contains thousands of genes putatively involved in interactions with insect hosts. Assembled unigenes were firstly aligned by blastx to protein databases like nr, Swiss-Prot, KEGG and COG, and the information about putative heterozygous nucleotide variations offered intriguing leads for the analysis of transcriptomic events and their effects on biological functions of *H. sinensis*. This study gave a new way to find secondary metabolism genes and clarify the functions of the corresponding metabolic pathways in *H. sinensis*. In addition, this study also provided a scientific basis for medicinal mechanism of *H. sinenesis* and effective protection for the sustainable use of *O. sinensis* resources. The availability of transcriptome sequence opens new avenues for new exploration, application and improvements of *H. sinensis*. It will lead to the identification and manipulation of candidate genes or genomic regions to generate the new ways to synthesize new compounds with potentials in pharmaceuticals, and pave foundation for development of new drugs for the pharmaceutical manufacturing or provide the theoretical basis for the sustainable use of *O. sinensis*.

The sequences of the *H. sinensis* transcriptome were captured by preparing RNA-Seq libraries from three different cultivation periods. Compared with worm-part and grass-part of *O. sinensis* cDNA libraries, *H. sinensis* transcriprome contained more genes which involved in translation, transcription and cell division etc. [28]. This study indicated that *H. sinensis* is more active in translation, transcription and cell division etc. Compared to other insect pathogens which have similar infection processes and life cycles to *H. sinensis*, such as *P. chrysogenum* [42], *Aspergillus niger* [43], *Metarhizium anisopliae* [44], *M. acridum* [44], *C. militaris* [40] and asexual strain of *O. sinensis* [39], *H. sinensis* has similar quantities of functional genes involved in transportation, cell metabolism, transcription and protein fate, while has larger quantities of functional genes involved in cell component, homeostasis and protein synthesis. This phenomenon indicates that *H. sinensis* could secret and transport more proteins for infecting the host larvae and propagating in host *vivo* as compared to these fungi.

The life cycle of *H. sinensis* is poorly understood aside from knowing that *H. sinensis* infects *H. armoricanus* pupae [45]. Its survival in soil may depend on the asexual stage of *O. sinensis* which provides resilient long-lived ascospores as described in other fungi [46]. As the asexual type of *O. sinensis*, in the nature, *H. sinensis* mainly infects host larvae in the soil through the skin of the head by the effects of enzymes and mechanical force when *H. sinensis* grows [47,48]. It is reported that the infection process of *H. sinensis* can be divided into three stages including attaching to the epidermis of larvae, penetrating body wall and propagation *in vivo* causing the death of host. The entire process includes host recognition, mechanical damage, nutrient competition, disturbing metabolism, secretion of toxins and the damaging the tissue structure of the host, these multi-factors interaction ultimately lead to host death and form the matured *O. sinensis* [49]. In the process of invading the body wall of insect, *H. sinensis* secretes many kinds of exocellular hydrolytic enzymes such as protease [50], chitinase [51] and lipase etc. which play important roles in the process of invading. Degradation of proteases is not only helpful to the mycelium penetration, but also provides nutrients for mycelial growth [52]. After screening transcriptome of *H. sinensis*, 157 proteases, 52 chitinases and 124 lipases were predicted and confirmed, comparing to *O. sinensis* whose gene families encoding 19 cuticle degrading proteases and 9 chitinases [39], indicating that *H. sinensis* has stronger infecting virulence. 7 chitinase genes, 7 protease genes and 3 lipase genes were successfully cloned and expressed in *E. coli* BL21 and the corresponding enzyme activities were successfully detected. Therefore, the results obtained in this study demonstrated that *H. sinensis* could produce these enzymes to accomplish the invasion.

Studies based on the molecular level have confirmed that nucleosides [53], sterols [54], polysaccharides [55] and mannitol [54] were the material basis for pharmacological effects of *H. sinensis*. At present, there are few reports about metabolic pathways both in *H. sinensis* and *O. sinensis*, so it is much more necessary and urgent to investigate metabolic pathways which will provide new information and method to further regulate, control and optimize the fermentation process to obtain *H. sinensis* mycelium with high quality. In order to model the biosynthesis of cordycepin, Zheng et al. [40] constructed the purine metabolic pathway in *C. militaris* based on the KEGG annotations, and suggested that the biosynthesis of cordycepin proceeds through a reductive mechanism as described for the formation of 2′-deoxyadenosine [56]. Hu et al. [46] found *O. sinensis* has multiple polyketide synthases, modular non-ribosomal peptide synthases and terpene cyclases for producing an array of secondary metabolites, these enzymes likely play roles after the latent period when the fungus is colonizing and killing the host, and they are also potential candidates for production of pharmacologically active ingredients. According to the transcriptome annotation results, we predicted the corresponding metabolic pathways of mannitol, cordycepin, purine nucleotides, pyrimidine nucleotides, unsaturated fatty acid, cordyceps polysaccharide and sphingolipid in *H. sinensis* for the first time, as well as the enzymes involved in each step, which can be concluded that this study provided a theoretical basis for regulation of metabolic pathways. One of the main pharmaceutically active ingredients of *H. sinens* is cordycepin [57], which is structurally similar to 2′-deoxyadenosine. *H. sinensis* possesses most of the genes required for metabolism of adenine and adenosine (Additional file 4: Figure S8). For metabolic pathway of cordycepic acid in *H. sinensis*, mannitol-1-phosphate phosphatase catalyzed D-mannitol-1-P to D-mannitol was not annotated in *H. sinensis* transcriptome, which indicated that other phosphatases took place the role of mannitol-1-phosphate phosphatase or the mannitol-1-phosphate phosphatase gene of *H. sinensis* was un-annotated to protein database since its low homology with currently reported mannitol-1-phosphate phosphatases from other organisms. In order to ensure the up- and down-regulated genes which encoding the enzymes involved in cordycepin metabolism pathway, real-time PCR was carried out to determinate differential expression genes [58]. The genes which encoding 5′-nucleotidase and N-glycosylase were detected obviously up-regulated which consistent with the RNA-Seq analysis. In this present study, 5′-nucleotidase and N-glycosylase were up-regulated 15.03 folds and 7.31 folds in different cultivation periods (9d-VS-3d) of *H. sinensis*, respectively. These two enzymes were significantly up-regulated when compared with other enzymes involved in cordycepin metabolic pathway. This result indicated that 5′-nucleotidase and N-glycosylase played important roles in cordycepin metabolic pathway, enhancing the expression of these genes could promote biosynthesis of cordycepin. This method was helpful to ensure the gene expression levels of DEGs which could be controlled to regulate the expression level of different enzymes and achieve the desired purpose. In addition, this result will pave the theoretical foundation to carry out further research on secondary metabolic mechanisms.

As a psychrophilic fungus, *H. sinensis* could secret many low-temperature enzymes which involved in cold tolerance mechanism. It is found that large numbers of lipid droplets were contained in *H. sinensis* cells, as described in other fungi adapted to long-term survival in frigid conditions [59]. Compared with other fungi, *H. sinensis* has fewer lipases (7 vs. average 42 in other fungi) and fatty acid hydroxylases (3 vs. average 14 in other fungi), but it has a larger range and number of genes involved in triacylglycerol and fatty acid biosynthesis consistent with an emphasis on synthesizing rather than degrading lipids, and the *H. sinensis* transcriptome was enriched in fatty acid desaturases (10 vs. average 3 in other fungi) when compared with cold-adapted plants and bacteria [60], indicating this fungus may respond to low temperatures by increasing membrane lipid instauration [39,45]. Considering the above possibilities, the cold tolerance mechanism of *H. sinensis* may lead to low demand of lipases and fatty acid hydroxylases, and the numbers of lipases and fatty acid hydroxylases are sufficient to *H. sinensis* adapting in frigid conditions. *H. sinensis* also could secrete many low-temperature enzymes involved in the metabolism which provides the basic energy for the survival. We screened the transcriptome annotation results and found some genes of low-temperature enzymes including 2 malate dehydrogenase genes, 6 ethanol dehydrogenase genes, 5 citroyl synthetase genes and 7 chitinase genes etc., which were further obtained and verified in this study. Therefore, the analysis of transcriptomes demonstrated that *H. sinensis* has the genes encoding these low-temperature enzymes to resist to the low temperature and protect itself in the cold environment.

## Conclusions

In this study, three transcriptomes of *H. sinensis* at different cultivation periods were sequenced for the first time, and the extensive transcriptomic analysis demonstrated that *H. sinensis* may have important systemic effects on *O. sinensis* at the level of genes. The genes which encoding the enzymes involved in the biosynthesis of active ingredients were predicted according to the annotation results, and the metabolic pathways of mannitol, cordycepin, purine nucleotides, pyrimidine nucleotides, unsaturated fatty acid, cordyceps polysaccharide, and sphingolipid in *H. sinensis* were described. Based on the predictions, we further investigated the interaction of different pathway networks and the corresponding enzymes. Many key or important genes involved in metabolic pathways, infection mechanism and cold tolerance mechanism were found by investigating the comparative differential expression genes from different cultivation periods. 764 DEGs between 3d and 6d, 1,869 DEGs between 3d and 9d, and 770 DEGs between 6d and 9d were found and confirmed, respectively. These findings provide a substantial contribution and basis to further characterize the gene expression profiles for *H. sinensis* in the metabolic pathways of active ingredients.

## Methods

### Strains, medium and culture condition

No vertebrates or any regulated invertebrates subjects were involved in the culture and sample preparation of the *O. sinensis* and *H. sinensis* during the whole process of this study. All procedures were performed within the research guidelines of the Zhejiang University of Technology, China and did not require approval of an ethics committee.

Wild *O. sinensis* samples were collected from Qinghai-Tibet plateau in Qinghai province during May (early worm season). The wild *O. sinensis* was collected and placed in a portable refrigerator, brought back to the lab and stored at 4°C, and the isolation process of *H. sinensis* was mentioned in Additional file 4: Figure S1-S20. *H. sinensis* L0106 was isolated from the wild *O. sinensis* and identified according to morphology, physiology (Additional file 5: Table S4) and its 18S rRNA sequence. The *H. sinensis* L0106 strain was deposited at the China Center for Type Culture Collection (Wuhan, China) under accession No. CCTCC M 2011278. *H. sinensis* was grown on the defined medium with glucose and corn powder as carbon sources, and dried silkworm chrysalis meal and fish meal as nitrogen sources using 200-liter submerged stirred fermentors at controlled pH 7.0 at 16°C. Biomass samples for transcriptome analysis were taken after 3 days, 6 days and 9 days. *Escherichia coli* JM109 (Invitrogen, Carlsbad, CA) was used as the host for plasmid pMD18-T (Invitrogen), and *Escherichia coli* BL21 (DE3) (Invitrogen) was employed as a host for expression of pET28a (Invitrogen). *E. coli* transformants were grown in LB medium at 37°C with shaking (200 rpm).

### Transcriptome sequencing and analysis

Total RNA samples from pure cultured 3 days (growth period), 6 days (pre-stable period) and 9 days (stable period) of *H. sinensis* were isolated using a standard TRIzol method and further qualified by UV determination at 260 nm and 280 nm and formaldehyde gel electrophoresis, respectively. 1 mg of the high-quality total RNA was dissolved in 500 μl of the *RNase*-free water, and then incubated in a water bath (Blue Pard Ltd, Shanghai, China) at 65°C for 10 min. For Illumina sequencing, the total RNA extracted using TRIzol was treated with *RNase*-free DNase I (TaKaRa) for 30 min according to the manufacturer’s protocols. The integrity of total RNA was checked using Agilent Technologies 2,100 Bioanalyzer (Agilent Technologies, Palo Alto, CA), and the RNA Integrity Number (RIN) value greater than eight. The mRNA was isolated from total RNA using Promega PolyATtract mRNA Isolation Systems [61]. The detailed procedure was carried out according to the technical manual of Promega PolyATtract mRNA Isolation Systems.

The cDNA libraries were prepared according to the manufacturer’s instructions (Illumina, San Diego, CA). The poly(A) containing mRNA molecules was purified using Sera-mag Magnetic Oligo(dT) Beads (Illumina) from 20 mg total RNA of each sample. 10 mM Tris–HCl was used to elute the mRNA from the magnetic beads. To avoid priming bias when synthesizing cDNA, the mRNA was first fragmented before cDNA synthesis. The mRNA was fragmented into small pieces using divalent cations at elevated temperature. The cleaned mRNA fragments primed by random primers were converted to double-stranded cDNA using SuperScript II (Invitrogen), *RNase* H (Invitrogen) and DNA Pol I (Invitrogen). The resulting cDNA was purified using the QIAquick PCR Purification Kit (Qiagen, Hilden, Germany). cDNA was then subjected to end-repair and phosphorylation using T4 DNA polymerase, Klenow DNA polymerase and T4 PNK, and subsequent purification was performed using QIAquick PCR Purification Kit (Qiagen). These repaired cDNA fragments were 30-adenylated using Klenow Exo- (Illumina) and purified using MinElute PCR Purification Kit (Qiagen), producing cDNA fragments with a single ‘A’ base overhung at their 30-ends for subsequent adapter-ligation. Illumina PE adapters were ligated to the ends of these 30-adenylated cDNA fragments, followed by purification using MinElute PCR Purification Kit (Qiagen). To select a size range of templates for downstream enrichment, the products from ligation reactions were purified on a 2% TAE-agarose gel (Certified Low-Range Ultra Agarose, Biorad). A range of cDNA fragments (200 ± 25 bp) was selected from the gel and extracted using QIAquick Gel Extraction Kit (Qiagen). Fifteen rounds of PCR amplification were performed to enrich the adapter modified cDNA library using primers complementary to the ends of the adapters. The PCR products of size 200 ± 25 bp were purified using QIAquick Gel Extraction Kit (Qiagen) except that Qiaquick spin columns were substituted with MinElute spin columns (Qiagen). Finally, after quantification on an Agilent Technologies 2,100 Bioanalyzer using the Agilent DNA 1,000 chip kit, the cDNA library products were sequenced using the 1G Illumina Genome Analyzer. Two biological replicates of every sample were analyzed.

After checking and filtering steps, the SOAPdenovo software (http://soap.genomics.org.cn/soapdenovo.html) was used to carry out the whole transcriptome assembly [62]. The contigs without any gap were obtained after SOAPdenovo assembly and correction. Subsequently, the obtained reads were aligned onto the contigs sequences and aligned paired ends. Meanwhile, the relationship and rate of consistent between each pair of contigs were evaluated, and then the scaffolds were constructed step by step. Paired-end reads were used again for gap filling of scaffolds to get sequences with least Ns and cannot be extended on either end. Such sequences were defined as unigenes.

### Unigene function annotation

Unigene annotation provides information of expression and functional annotation of unigene. Information of functional annotation includes protein functional annotation, COG functional annotation and Gene Ontology (GO) (http://geneontology.org/) functional annotation of unigenes. Unigene sequences are firstly aligned by blastx to protein databases like nr (http://www.ncbi.nlm.nih.gov/sites/entrez?db=protein), Swiss-Prot (http://www.expasy.org), KEGG (http://www.kegg.jp/) and COG (E-value < 0.00001) (http://www.ncbi.nlm.nih.gov/COG/), retrieving proteins with the highest sequence similarity with the given unigenes along with their protein functional annotations. KEGG database contains systematic analysis of inner-cell metabolic pathways and functions of gene products, which helps to study the complicated biological behaviors of genes. Pathway annotation of unigenes was obtained by KEGG database, and then the unigenes are aligned to COG database to predict and classify possible functions.

### Gene ontology analysis

The gene ontology terms of each *H. sinensis* gene were obtained by the software Blast2GO (http://www.blast2go.com) using the default parameters. Blast2GO was also used for GO functional enrichment analysis of certain genes, by performing Fisher’s exact test with robust false discovery rate (FDR) correction to obtain an adjusted *p*-value between certain test gene groups and the whole annotation.

### Pathway analysis with KEGG

Pathway analysis was carried out according to KEGG mapping method [63]. Enzyme commission (EC) numbers were assigned to unique sequences that had BLASTX scores with cutoff values of E < 1.0e5, as determined based on searching the protein databases. The sequences were mapped to the KEGG biochemical pathways according to the EC distribution in the pathway database.

### Normalized expression level of gene by RNA-Seq

The expression level of gene by RNA-Seq was normalized by the number of reads per kilobase of exon region per million mapped reads (RPKM) [64]. The cutoff value for determining gene transcriptional activity was determined based on a 95% confidence interval for all RPKM values of each gene.

### Discovery of novel transcripts

Novel transcripts can be found by high throughput sequencing [24] since present databases may be incomplete. Gene models found in intergenic regions (200 bp away from upstream or downstream genes) were thought to be candidate of novel transcripts.

### Refinement of gene structures

The gene structure was optimized according to the distribution of the reads, information of paired-end and the annotation of reference gene. The distribution of reads in the transcriptome was obtained by aligning the continuous and overlap reads form a Transcription Active Region (TAR). According to paired-end data, different TARs were connected to form a potential gene model. Subsequently, the gene model with the existing gene annotated to extend the gene 5′ and 3′ end was further compared.

### Alternative splicing analysis

Alternative splicing analysis [65] was developed in this study. There are mainly four types of alternative splicing in the *H. sinensis* transcriptome analysis including ES, IR, A5SS and A3SS. The following algorithms were used to detect alternative splicing events. First, junction sites giving information about boundaries and combinations of different exons in a transcript, are detected by TopHat (with all default parameters) [66]. All junction sites of the same gene are used to distinguish type of its alternative splicing event. If junctions were detected, it indicated that exon skipping occurred in transcript. If the junctions meet the following conditions, there is an Intron Retention event between Exon1 and Exon2. 1) Junction 1 is detected, which means there is an intron between Exon1 and Exon2; 2) 90% of this intron is covered by unique-mapping reads; 3) the coverage depth of the intron is at least 15% of the coverage depth of Exon1 or Exon2; 4) 5 bp both upstream and downstream of this intron’s both boundaries is required to be covered by reads; 5) the intron region cannot be covered by other genes. If either Junction 2 or Junction 3, which has the same 3′ but different 5′ splice sites with Junction 1, is detected, then there is an Alternative 5′ Splice Site event between Exon1 and Exon2. If either Junction 2 or Junction 3, which has the same 5′ but different 3′ splice sites with Junction 1, is detected, then there is an Alternative 3′ Splice Site event between Exon1 and Exon2.

### Verification of predicted enzymes in metabolic pathways

In this study, we used bioinformatics softwares including DNAMAN (Version 5.2.2, Lynnon Biosoft, Canada) and Primer 5.0 (http://www.primer-e.com), and online bioinformatic tools such as KEGG pathway database (http://www.kegg.jp/), Blast (http://blast.ncbi.nlm.nih.gov/Blast.cgi) and ORF Finder (http://www.ncbi.nlm.nih.gov/gorf/gorf.html) to analyze metabolic pathways of seven main bioactive ingredients including mannitol, cordycepin, purine nucleotides, pyrimidine nucleotides, unsaturated fatty acid, cordyceps polysaccharide and sphingolipid in *H. sinensis*. The functional genes and enzymes which were involved in metabolic pathways of *H. sinensis* were then verified by using gene cloning and protein expression according to the previously reported methods [67]*.*

PCR was performed to validate the genes which can encode the enzymes involved in different pathways. Primers were designed according to the predicted ORF sequences of target genes (Additional file 6: Table S5-S7). Genes related to synthesis pathways were cloned by PCR amplification method. Subsequently, the genes were sub-cloned to the pMD18-T vector (Invitrogen). Finally, the recombinant plasmid was successfully transformed to *E. coli* JM109 competent cells (Invitrogen). The recombinant *E. coli* JM109 was cultivated in the shaker at 200 rpm and then the recombinant plasmids were extracted using plasmid extraction kit (Qiagen). After sequencing, the target genes were sub-cloned into the pET28a (Invitrogen), the recombinant plasmid was also successfully transferred to *E. coli* BL21 competent cells (Invitrogen). Then the genes were expressed in *E. coli* BL21 using IPTG as inducer. The expressed proteins were further detected by SDS-PAGE [68].

### Sequence submission

The 18S rRNA sequence of *H. sinensis* has been deposited in a public repository of NCBI GenBank Database (http://www.ncbi.nlm.nih.gov/WebSub/?tool=genbank) with a GenBank accession number of KP090933. The gene and protein sequences of hexokinase (HK-1), hexokinase (HK-3), hexokinase (HK-4), hexokinase (HK-5), hexokinase (HK-6), hexokinase (HK-8), glucosephosphate isomerase (GPI-1), glucosephosphate isomerase (GPI-2), glucosephosphate isomerase (GPI-3), mannitol-1-phosphate 5-dehydrogenase (mtlD-1), mannitol-1-phosphate 5-dehydrogenase (mtlD-2), ADP-ribose pyrophosphatase (ADPR-PPase), ribose-phosphate pyrophosphokinase (PRPS-1), ribose-phosphate pyrophosphokinase (PRPS-2), amidophosphoribosyltransferase (purF-2), amidophosphoribosyltransferase (purF-3), phosphoribosylamine-glycine ligase (purD-1), phosphoribosylglycinamide formyltransferase (GAR TFase-1), phosphoribosylformylglycinamidine synthase (purL-1), phosphoribosylformylglycinamidine cyclo-ligase (purM-1), phosphoribosylaminoimidazole carboxylase (PAICS-1), phosphoribosylaminoimidazole-succinocarboxamide synthase (purC-1), adenylosuccinate lyase (purB-1), phosphoribosylaminoimidazolecarboxamide formyltransferase (purH-1), adenylosuccinate synthase (purA-2), 5′-nucleotidase (5′-Nuc-1), 5′-nucleotidase (5′-Nuc-2), N-glycosylase/DNA lyase (N-Gly-1), purine nucleosidase (iunH-1), adenosine kinase (ADK-1), adenine phosphoribosyltransferase (APRT-1), AMP deaminase (AMPD-1), AMP deaminase (AMPD-6), IMP dehydrogenase (guaB-1), IMP dehydrogenase (guaB-2), GMP synthase (guaA-1), GMP synthase (guaA-2), GMP synthase (guaA-3), guanine deaminase (guaD-1), guanine deaminase (guaD-2) and xanthine dehydrogenase/oxidase (XDH-1), which were verified by experiments in this study, have been deposited in the NCBI GenBank Database, and the GenBank accession numbers for these sequences were recorded as KP090934, KP090935, KP090936, KP090937, KP090938, KP090939, KP090940, KP090941, KP090942, KP090943, KP090944, KP090945, KP090946, KP090947, KP090948, KP090949, KP090950, KP090951, KP090952, KP090950, KP090953, KP090954, KP090955, KP090956, KP090957, KP090958, KP090959, KP090960, KP090961, KP090962, KP090963, KP090964, KP090965, KP090966, KP090967, KP090968, KP090969, KP090970, KP090971, KP090972 and KP090973, respectively. The detailed gene and protein sequences were shown in Additional file 7.

## Additional files

Additional file 1: Table S1. Unigene annotations provide functional annotations of unigene (All) and expression levels. Functional annotations of unigene including protein sequence similarity, KEGG Pathway, COG and Gene Ontology (GO). Additional file 2: Table S2. COG function classification of *H. sinensis* unigenes (All) compared with *O. sinensis* grass-part (OSGP) and *O. sinensis* worm-part (OSWP). Additional file 3: Table S3. Statistics of *H. sinensis* transcriptome mapped to reference genome and reference gene. Additional file 4: Figures S1-S20. Supplementary results and methods, figure legends for supplementary figures and tables. Additional file 5: Table S4. Biolog metabolic fingerprinting analysis of *H. sinensis*. Additional file 6: Table S5. The primers used for cloning and expressing genes involved in mannitol metabolic pathway. **Table S6.** The primers used for cloning and expressing genes involved in cordycepin metabolic pathway. **Table S7.** The primers used for cloning and expressing genes involved in purine nucleotides metabolic pathway. **Table S8.** The primers used for real-time PCR involved in mannitol metabolic pathway. **Table S9.** The primers used for real-time PCR involved in cordycepin metabolic pathway. **Table S10.** The primers used for real-time PCR involved in purine nucleotides metabolic pathway. Additional file 7:
**List of 18S rRNA gene, mannitol anabolic functional genes, cordycepin anabolic functional genes and purine nucleotides anabolic functional genes including GenBank accession numbers.**
