# Supplementary material for: Transcriptome sequencing and analysis of the entomopathogenic fungus Hirsutella sinensis isolated from Ophiocordyceps sinensis
Source: BMC Genomics. 2015 Feb 21;16(1):106. doi: 10.1186/s12864-015-1269-y (PMC4342880; doi:10.1186/s12864-015-1269-y)
Supplement: Additional file 2: Table S2. — COG function classification of H. sinensis unigenes (All) compared with O. sinensis grass-part (OSGP) and O. sinensis worm-part (OSWP). [file 12864_2015_1269_MOESM2_ESM.doc]

### Additional file 2: Table S2 COG function classification of *H. sinensis* unigenes (All) compared with *O. sinensis* grass-part (OSGP) and *O. sinensis* worm-part (OSWP).

| **Class definitions** | ***H. sinensis*** | **OSGP** | **OSWP** |
| --- | --- | --- | --- |
| General function prediction only | 2,273 | 141 | 138 |
| Translation, ribosomal structure and biogenesis | 1,479 | 152 | 113 |
| Posttranslational modification, protein tur nover, chaperones | 1,004 | 98 | 96 |
| Carbohydrate transport and metabolism | 1,048 | 62 | 79 |
| Energy production and conversion | 537 | 60 | 79 |
| Amino acid transport and metabolism | 956 | 50 | 63 |
| Transcription | 1,794 | 35 | 33 |
| Inorganic ion transport and metabolism | 456 | 26 | 33 |
| Lipid transport and metabolism | 741 | 20 | 36 |
| Replication, recombination and repair | 1,404 | 24 | 27 |
| Function unknown | 1,810 | 21 | 27 |
| Coenzyme transport and metabolism | 286 | 14 | 30 |
| Nucleotide transport and metabolism | 192 | 17 | 21 |
| Intracellular trafficking, secretion, and vesicular transport | 580 | 20 | 22 |
| Signal transduction mechanisms | 906 | 22 | 12 |
| Secondary metabolites biosynthe sis, transport and catabolism | 500 | 15 | 17 |
| Cytoskeleton | 184 | 13 | 12 |
| Cell cycle control, cell division, chromosome partitioning | 1,465 | 16 | 12 |
| Cell wall/membrane/envelope biogenesis | 950 | 10 | 13 |
| Chromatin structure and dynamics | 98 | 13 | 4 |
| RNA processing and modification | 48 | 7 | 4 |
| Defense mechanisms | 107 | 2 | 4 |
| Cell motility | 150 | 2 | 0 |
| Nuclear structure | 4 | 1 | 1 |
