# Supplementary material for: Transcriptome sequencing and analysis of the entomopathogenic fungus Hirsutella sinensis isolated from Ophiocordyceps sinensis
Source: BMC Genomics. 2015 Feb 21;16(1):106. doi: 10.1186/s12864-015-1269-y (PMC4342880; doi:10.1186/s12864-015-1269-y)
Supplement: Additional file 3: Table S3. — Statistics of H. sinensis transcriptome mapped to reference genome and reference gene. [file 12864_2015_1269_MOESM3_ESM.doc]

### Additional file 3: Table S3 Statistics of *H. sinensis* transcriptome mapped to reference genome and reference gene.

|  | **Map to Genome** | | **Map to Gene** | |
| --- | --- | --- | --- | --- |
|  | **reads number** | **percentage** | **reads number** | **percentage** |
| 3d |  |  |  |  |
| Total Reads | 26,577,778 | 100.00% | 26,577,778 | 100.00% |
| Total BasePairs | 2,392,000,020 | 100.00% | 2,392,000,020 | 100.00% |
| Total Mapped Reads | 18,949,871 | 71.30% | 8,771,448 | 33.00% |
| perfect match | 13,031,334 | 49.03% | 5,861,924 | 22.06% |
| <=5bp mismatch | 5,918,537 | 22.27% | 2,909,524 | 10.95% |
| unique match | 17,285,218 | 65.04% | 8,384,162 | 31.55% |
| multi-position match | 1,664,653 | 6.26% | 387,286 | 1.46% |
| Total Unmapped Reads | 7,627,907 | 28.70% | 17,806,330 | 67.00% |
| 6d |  |  |  |  |
| Total Reads | 27,355,556 | 100.00% | 27,355,556 | 100.00% |
| Total BasePairs | 2,462,000,040 | 100.00% | 2,462,000,040 | 100.00% |
| Total Mapped Reads | 19,380,240 | 70.85% | 8,630,354 | 31.55% |
| perfect match | 13,250,776 | 48.44% | 5,731,706 | 20.95% |
| <=5bp mismatch | 6,129,464 | 22.41% | 2,898,648 | 10.60% |
| unique match | 17,445,855 | 63.77% | 8,234,999 | 30.10% |
| multi-position match | 1,934,385 | 7.07% | 395,355 | 1.45% |
| Total Unmapped Reads | 7,975,316 | 29.15% | 18,725,202 | 68.45% |
| 9d |  |  |  |  |
| Total Reads | 16,809,688 | 100.00% | 16,809,688 | 100.00% |
| Total BasePairs | 1,260,726,600 | 100.00% | 1,260,726,600 | 100.00% |
| Total Mapped Reads | 13,146,918 | 78.21% | 5,532,921 | 32.92% |
| perfect match | 10,185,645 | 60.59% | 4,189,643 | 24.92% |
| <=5bp mismatch | 2,961,273 | 17.62% | 1,343,278 | 7.99% |
| unique match | 12,094,781 | 71.95% | 5,258,746 | 31.28% |
| multi-position match | 1,052,137 | 6.26% | 274,175 | 1.63% |
| Total Unmapped Reads | 3,662,770 | 21.79% | 11,276,767 | 67.08% |
