# Supplementary material for: Transcriptome sequencing and analysis of the entomopathogenic fungus Hirsutella sinensis isolated from Ophiocordyceps sinensis
Source: BMC Genomics. 2015 Feb 21;16(1):106. doi: 10.1186/s12864-015-1269-y (PMC4342880; doi:10.1186/s12864-015-1269-y)
Supplement: Additional file 4: Figures S1-S20. — Supplementary results and methods, figure legends for supplementary figures and tables. [file 12864_2015_1269_MOESM4_ESM.doc]

**Supplementary Material for Liu et al.**

# Transcriptome sequencing and analysis of the entomopathogenic fungus *Hirsutella sinensis* isolated from *Ophiocordyceps sinensis*

**Zhi-Qiang Liu^1^, Shan Lin^1^, Peter James Baker^1^, Ling-Fang Wu^1^, Xiao-Rui Wang^1^, Hui Wu^2^, Feng Xu^2^, Hong-Yan Wang^2^, Mgavi Elombe Brathwaite^3^, Yu-Guo Zheng^1§^**

^1^ Institute of Bioengineering, Zhejiang University of Technology, Hangzhou 310014, Zhejiang, P. R. China

^2^ East China Pharmaceutical Group Limited Co., Ltd, Hangzhou 311000, Zhejiang,P.R. China

^3^ Polytechnic School of Engineering, New York University, 6 MetroTech Center, Brooklyn, NY, 11201, USA

^§^ Corresponding author: Yu-Guo Zheng [zhengyg@zjut.edu.cn](mailto:zhengyg@zjut.edu.cn)

**Supplementary Results Pages 2**

**Supplementary Methods Pages 3 - 7**

**Supplementary Figures Pages 8 - 27**

**Supplementary Table Legends Page 28**

**Supplementary References Page 29**

**Supplemental Results**

**Isolation and Identification of *H. sinensis***

The teleomorph and anamorph strains from the stroma and sclerotium of *Ophiocordyceps sinensis* were isolated, respectively. The 18S rDNA gene sequences of teleomorph and anamorph of *O. sinensis* were amplified and used as BLAST queries against the NCBI database indicating that the two strains show 99% homology with *O. sinensis* (gi: 190612558/gb: EU570952.1), according to the life cycle of *O. sinensis*, the teleomorph and anamorph of *O. sinensis* were isolated, and the anamorph of *O. sinensis* was named *Hirsutella sinensis* L0106. The analysis of colonial morphology of *H. sinensis* was carried out (Figure S18), the color of single colonies was white, hyphae were fluffy and outward, and the diameter of the colonies ranged from 1 cm to 2 cm, indicating that colonial morphology of *H. sinensis* was similar to anamorph of *O. sinensis*. Biolog metabolic fingerprinting analysis of *H. sinensis* showed it could strongly use 26 kinds of carbon source (Additional file 5: Table S4), but could not use or weakly use other 69 kinds of carbon source, indicating that Biolog metabolic fingerprinting *H. sinensis* was similar to anamorph of *O. sinensis*. In addition, the mycelia of *H. sinensis* were clearly observed by electron microscope images (Figure S19), the SEM photographs showed that it exists in the form of mycelia, mycelia present a woven mesh, the diameter of the mycelium ranges from 1 to 2 μm, sporangium can be observed at the edge of the mycelium, *H. sinensis* presents a unique form of fungi. Phylogenesis analysis between *H. sinensis* and other entomogenous fungi was performed, and the phylogenetic tree showed that *H. sinensis* has a close genetic relationship to *O. sinensis*, *H. liboensis* and *H. minnesotensis* (Figure S20).

**Supplemental Methods**

**Sample collection and growth conditions**

The samples were collected during May (early worm season). The samples were collected from locations at the surface and various depths with maximum distance of 4 km. The temperature on the sampling sites varied between 11 and 17 °C in wet seasons and 28-34 °C in dry seasons. The pH of samples was 6.5-8.2. Samples were collected in sterile plastic containers and were cultured not later than 18 h after collection. All samples were cultured in a saline and transferred to sterilized poly-ethylene bags and transported to the laboratory.

**Preparation of isolation media**

The isolation media of potato dextrose agar (PDA) was prepared and then autoclaved at 115 °C for 30 min before use. Liquid PDA medium was composed 20% potatoes, 2.0 g/L glucose, 0.46 g/L KH_2_PO_4_, 0.5 g/L MgS0_4_, 10.0 mg/L VB_1_, and 1.0 mg/L K_2_HPO_4_, and solid PDA medium needs addition of 2% agar. The fermentation medium consisted of 1.0% glucose, 1.0% molasses, 0.5% silkworm chrysalis powder, 1.0% soybean meal, 0.5% yeast extract, 0.01% MgSO_4_, and 0.02% KH_2_PO_4_.

**Isolating and cultivating of *H. sinensis***

Fresh *O. sinensis* was selected for isolation of *H. sinensis*, and impurities on the surface of fruiting bodies were clean up by sterile water. Then fruiting bodies were washed several times with sterile purified water, and disinfection was carried out by conventional method by using 0.1% mercuric chloride. Subsequently, worms and stromata were correctly cut with sterile scalpel in sterile conditions, three parts of the tissues were picked and cultured on the sterilized PDA slant medium in 16 °C constant temperature incubator with daily growth observed and recorded. In addition, worms and stromata were broken apart with sterile forceps, and white mycelium tissues located in the center were directly taken and seeded in PDA medium.

When the cultured tissues were germinated after about 15 days, they were inoculated to liquid PDA medium by pure culture with the condition of 16 °C constant temperature shaking culture. Cultured medium became pale yellow and a little thick after 15 days, at this point, the inoculated tissue surface was covered with white mycelia. After 30 days culture, liquid mycelia were inoculated into solid PDA medium, and then the surface was covered with about 3 cm stromata after 20 days. Finally, several species identification methods, such as molecular identification, Biolog identification and morphological identification were carried out to identify the isolated strains whether were *H. sinensis*. After this procedure, it can be basically determined that the anamorph of *O. sinensis* named *H. sinensis* were successfully isolated.

In order to obtain more mycelium used in Chinese medicine, the isolated *H. sinensis* were inoculated into fermentation medium with the condition of 16 °C. *H. sinensis* was grown on the defined medium with glucose and corn powder as carbon sources, and dried silkworm chrysalis meal and fish meal as nitrogen sources using 200-liter submerged stirred fermentor at controlled pH 7.0 at 16 °C. Biomass samples for the transcriptome analysis were taken after 3 days, 6 days and 9 days.

**Real-time PCR**

Total RNA were firstly extracted from pure samples of *H. sinensis* cultiviated for 3 days, 6 days and 9 days using a standard TRIzol method and were then qualified by formaldehyde gel electrophoresis and UV determination at 260 nm and 280 nm, respectively. Then the mRNA from different samples were isolated from total RNA using Promega PolyATtract mRNA Isolation Systems, and the cDNA libraries were subsequently prepared according to the manufacturer’s instructions (Illumina). Meanwhile, the real-time PCR primers were designed using the Primer Express tool (Additional file 6: Table S8, Table S9 and Table S10). We selected the 18S rDNA gene expression level of *H. sinensis* as the internal control since other housekeeping genes such as *β*-tubulin, actin and GAPDH etc were not obtained by screening the transcriptome of *H. sinensis*. The relative expression levels were calculated by comparing the cycle thresholds (CTs) of the target genes with that of the housekeeping 18S rDNA gene, using the 2^-ΔΔCt^ method. Using the Student’s T-test, differences in relative transcript expression levels were compared at P<0.05 level between the growth period 3d and the stable period 9d.

10 μL of real-time PCR mixture was composed of 1 μl of cDNA from 3 days, 6 days and 9 days samples, respectively, 5 μl of SYBR Green PCR Master Mix (2×) (Promega Corporation), and 0.5 μl (100 μmol/L) of each forward and reverse primer. The real-time PCR was carried out according to the temperature-time profile as following: denaturation of 95°C for 2 min, 40 cycles of 95°C for 15 sec, and 60 °C for 1 min. The real-time PCR analyses were performed three times with independent RNA samples.

**Analysis of KEGG pathway**

Pathway-based analysis helps to further understand genes biological functions. KEGG is the major public pathway-related database of biological systems that integrates genomic, chemical and systemic functional information . KEGG provides a basic knowledge for linking genomes to life through the process of pathway mapping. Pathway enrichment analysis identifies significantly enriched metabolic pathways or signal transduction pathways in DEGs comparing with the whole genome background. The calculating formula is shown below:

N is the number of all genes that with KEGG annotation, n is the number of DEGs in N, M is the number of all genes annotated to specific pathways, and m is number of DEGs in M. And pathways with q-value ≤ 0.05 are significantly enriched in DEGs.

**GO functional classification**

Gene Ontology (GO) is an international standardized gene functional classification system which offers a dynamic-updated controlled vocabulary and a strictly defined concept to comprehensively describe properties of genes and their products in organisms. GO has three ontologies: molecular function, cellular component and biological process. The basic unit of GO is GO-term. Every GO-term belongs to a type of ontology. With nr annotation, we use Blast2GO program to get GO annotation of Unigenes. Then, we use WEGO software to do GO functional classification for all Unigenes and to understand the distribution of gene functions of the species from the macro level.

**Calculation of Unigene expression**

The RPKM method (Reads Per kb per Million reads) was used to calculate the Unigene expression , and the formula of RPKM is shown below:

$$RPKM=\frac{10^{6}C}{NL/10^{3}}$$

In this formula, RPKM (A) is the expression of Unigene A, and C is the number of reads that uniquely aligned to Unigene A, N is the total number of reads that uniquely aligned to all Unigenes, and L is the number of bases on Unigene A. The RPKM method is able to eliminate the influence of different gene length and sequencing level on the calculation of gene expression. Therefore, the calculated gene expression can be directly used for comparing the difference of gene expression between samples.

**Alignment of Unigenes**

When a Unigene happens to be unaligned to non of the above databases, a software named ESTScan will be introduced to predict its coding regions as well as to decide its sequence direction. For Unigenes with sequence directions, we provide their sequences from 5' end to 3' end, for those without any direction we provide their sequences from assembly software.

**Identification of differentially expressed genes**

We have developed a rigorous algorithm to identify differentially expressed genes between two samples using digital gene expression method . The number of unambiguous clean tag from gene A is set as x, as every gene's expression occupies only a small part of the library, the p(x) is in the Poisson distribution.

$$p(\mathrm{x})=\frac{e^{-\text{λ}}\text{λ}^{x}}{x!}$$

N1 represents the total clean tag number of the sample 1, and N2 represents total clean tag number of sample 2, gene A holds x tags in sample1 and y tags in sample 2. The probability of gene A expressed equally between two samples can be calculated with the following formula:

$2\sum_{i=0}^{i=y} p(\mathrm{i}\text{︱}\mathrm{x})$ Or $2\times\left(1-\sum_{i=0}^{i=y} p(\mathrm{i}\text{︱}\mathrm{x})\right)$ $\left(if\sum_{i=0}^{i=y} p(\mathrm{i}\text{︱}\mathrm{x})>0.5\right)$

$$p(y\text{︱}\mathrm{x})=\left(\frac{N_{2}}{N_{1}}\right)^{y}\frac{\left(x+y\right)!}{x!y!\left(1+\frac{N_{2}}{N_{1}}\right)^{\left(x+y+1\right)}}$$

*p*-value corresponds to differential gene expression test. FDR (False Discovery Rate) is a method to determine the threshold of *p*-value in multiple test and analysis through manipulating the FDR value. If R differentially expressed genes were picked out, and in which S genes were really show differential expression, while the other V genes were false positive, the error ratio should be "Q = V/R". If we wanted the error ratio to stay below a cutoff (1%), we should preset the FDR to a number no larger than 0.01. We use "FDR ≤ 0.001 and the absolute value of log_2_-ratio ≤ 1" as the threshold to judge the significance of gene expression difference. More stringent criteria with smaller FDR and bigger fold-change value can be used to identify DEGs.

**Supplemental Figures**

**Figure S1:** **Gene expression difference analysis among 3d-VS-6d, 9d-VS-3d and 9d-VS-6d.**

ExtendGene, Exon skipping and Intron retention analysis of 3d, 6d and 9d were compared, which were shown in A, B and C, respectively. And alternative 5' splice site, alternative 3' splice site and the number of transcripts analysis of 3d, 6d and 9d were also compared, which were shown in D, E and F, respectively. Finally, the comparison of differential expression genes, up-regulated and down-regulated genes analysis of 3d-VS-6d, 9d-VS-3d and 9d-VS-6d were carried out and shown in G, H and I, respectively.


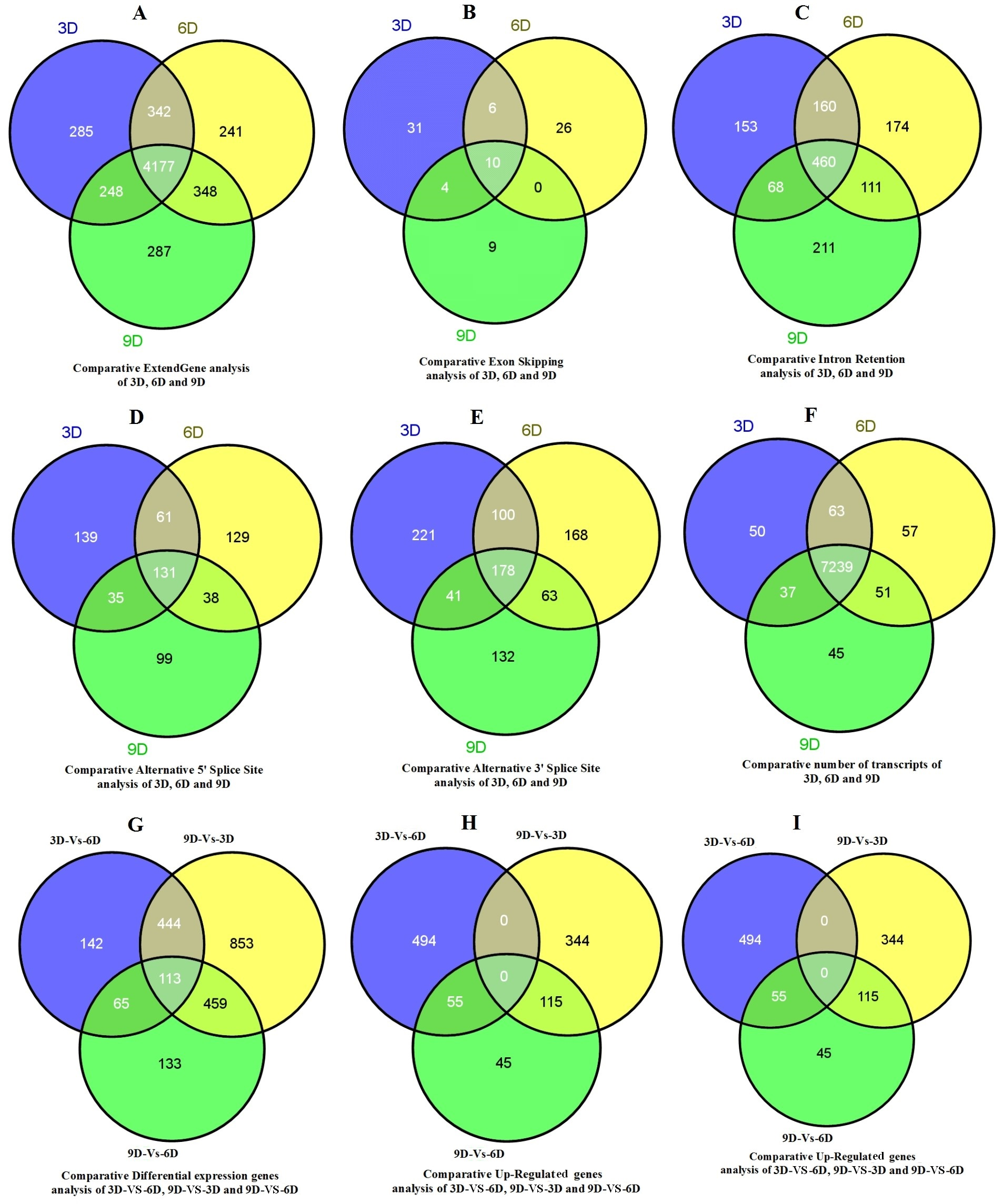


**Figure S2: Characteristics of *H. sinensis* DEGs’ GO functional enrichment.**

**
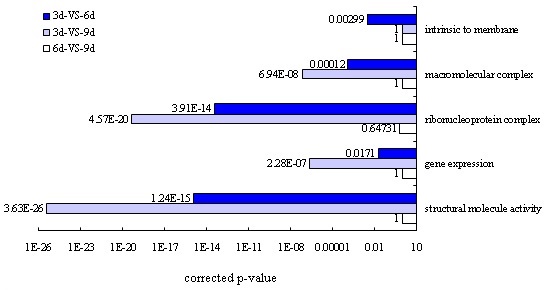
**

**Figure S3: Characteristics of *H. sinensis* DEGs’ KEGG pathway** **enrichment.**

**
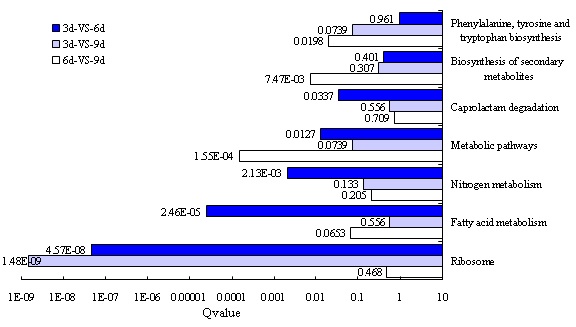
**

**Figure S4:** **The life cycle of *H. sinensis*.**

**
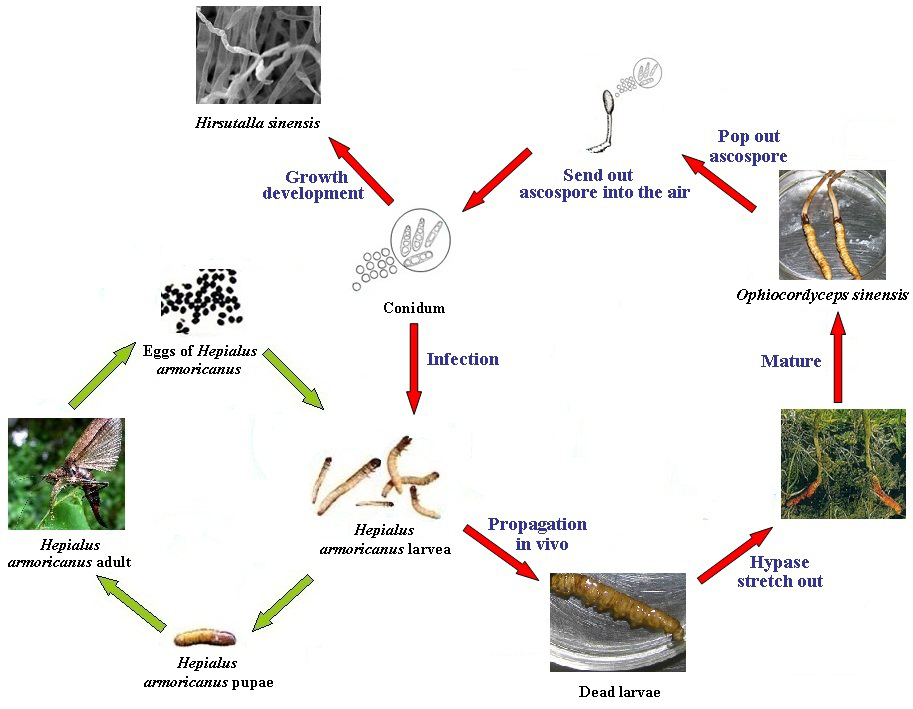
**

**Figure S5:** **Mannitol metabolic pathway of** ***H. sinensis*.**


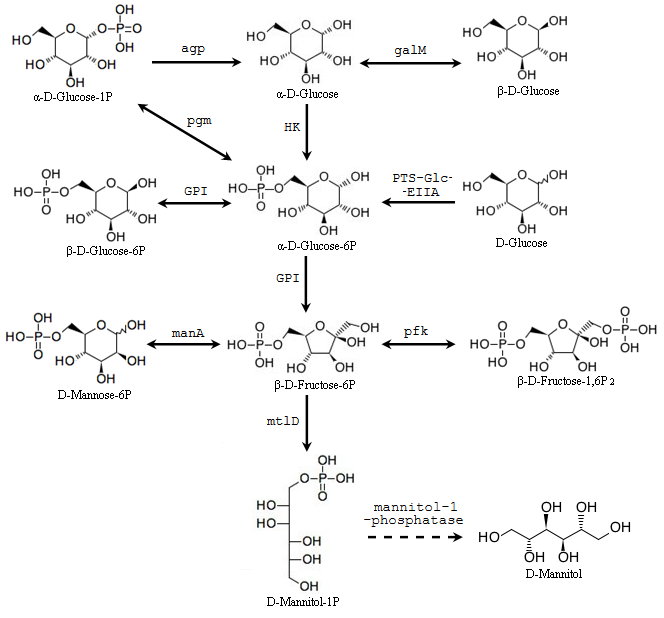


**Figure S6:** **Agarose gel electrophoresis of resulting PCR fragment of the mannitol** **anaboli****c functional genes from *H. sinensis*.**


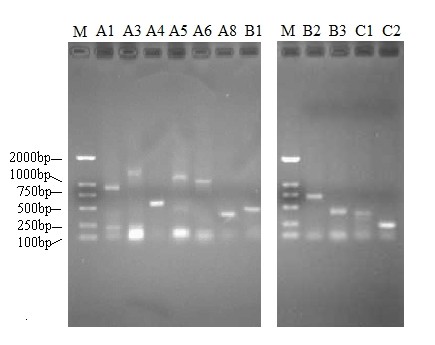


**Figure S7:** **SDS-PAGE analysis of expression products of mannitol anabolic functional genes from *H. sinensis*.**


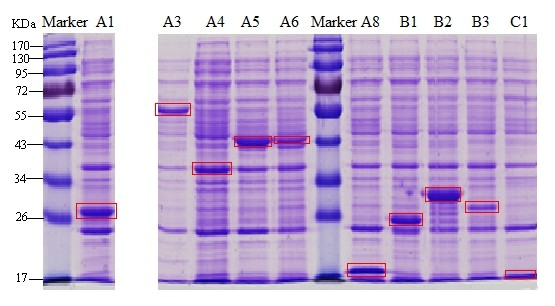


**Figure S8:** **Cordycepin metabolic pathway of *H. sinensis*.**


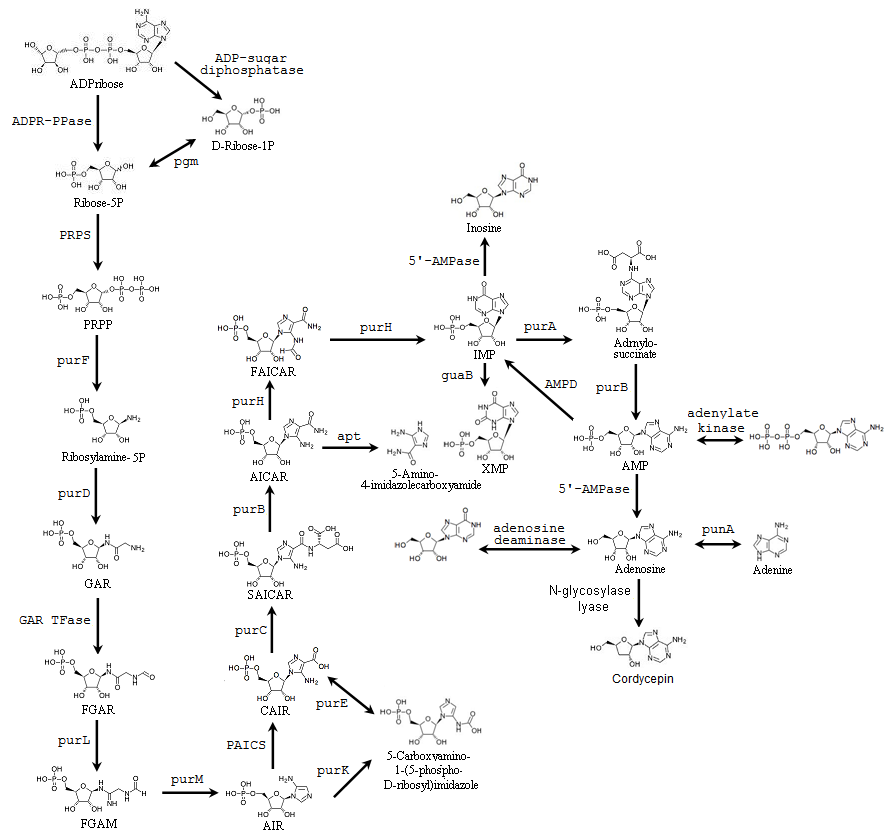


**Figure S9:** **Agarose gel electrophoresis of resulting PCR fragment of the cordycepin anabolic functional genes from *H. sinensis*.**


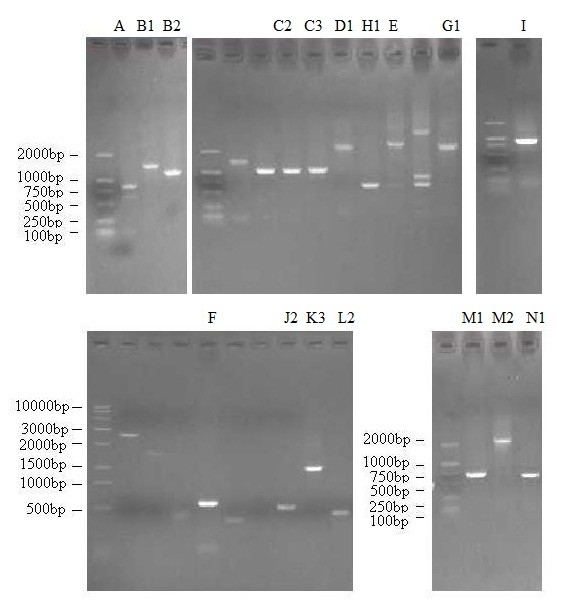


**Figure S10: SDS-PAGE analysis of expression products of cordycepin anabolic functional genes from *H. sinensis*.**


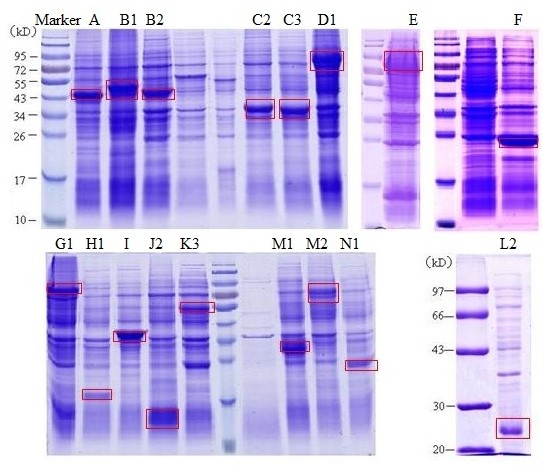


**Figure S11: Purine nucleotides metabolic pathway of *H. sinensis*.**

**
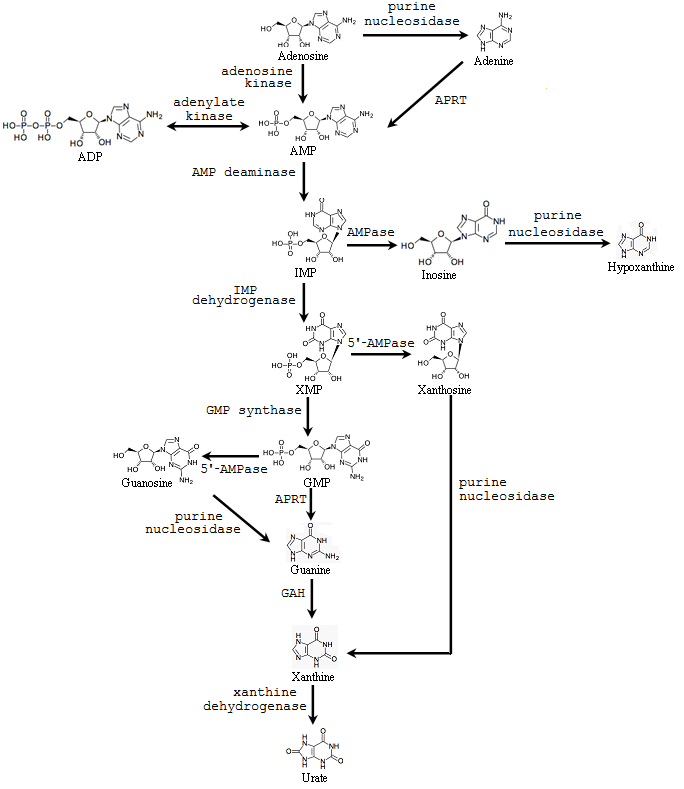
**

**Figure S12: Agarose gel electrophoresis of resulting PCR fragment of the purine nucleotides anabolic functional genes from *H. sinensis*.**

**
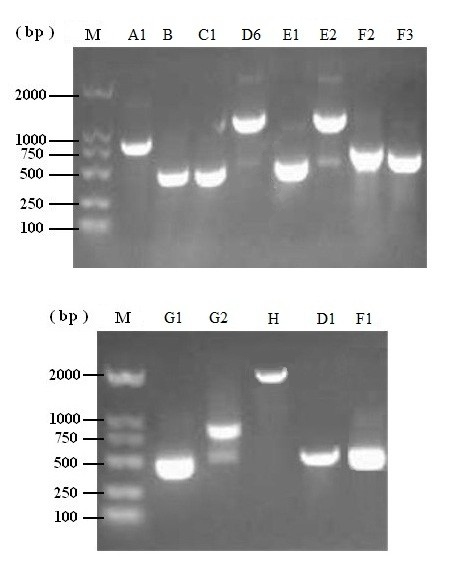
**

**Figure S13: SDS-PAGE analysis of expression products of purine nucleotides anabolic functional genes from *H. sinensis*.**


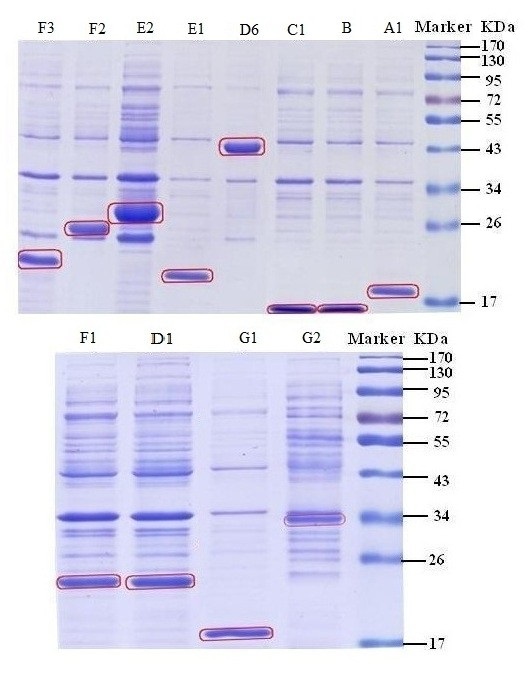


**Figure S14: Pyrimidine nucleotides metabolic pathway of *H. sinensis*.**

**
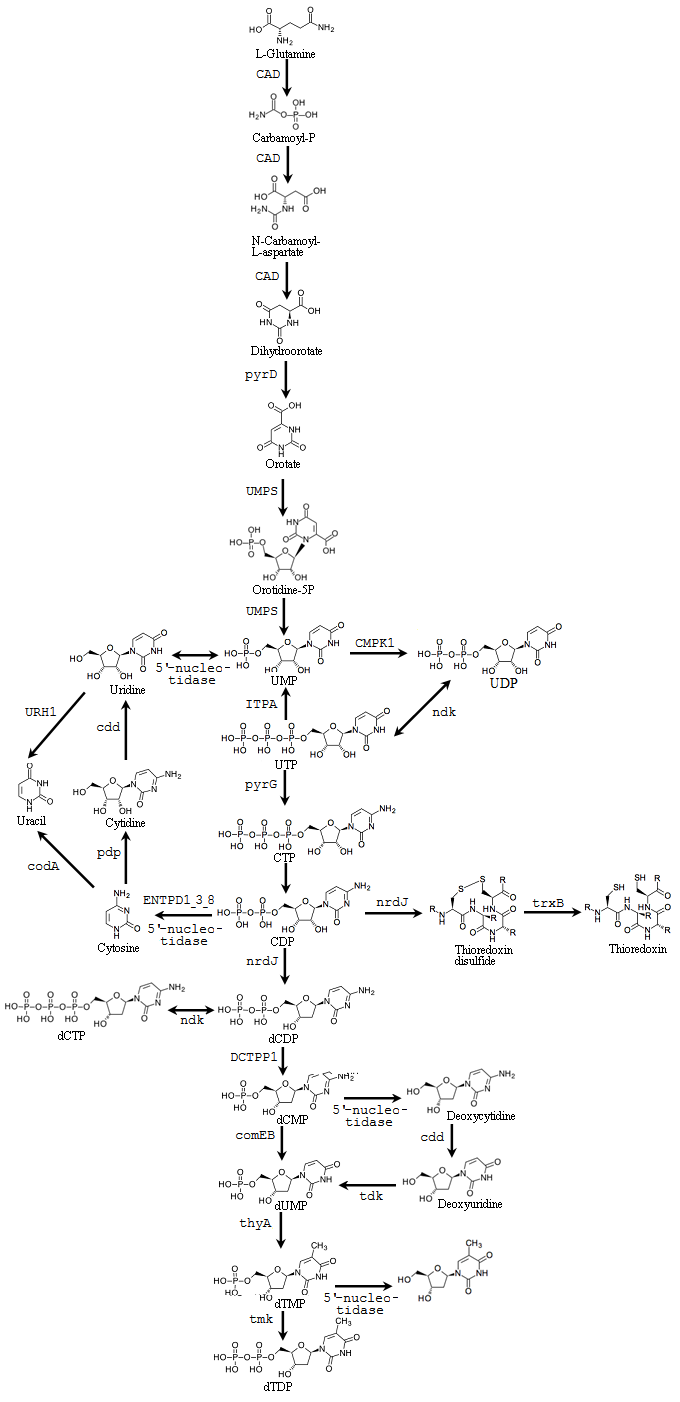
**

**Figure S15: Unsaturated fatty acid metabolic pathway of *H. sinensis*.**

**
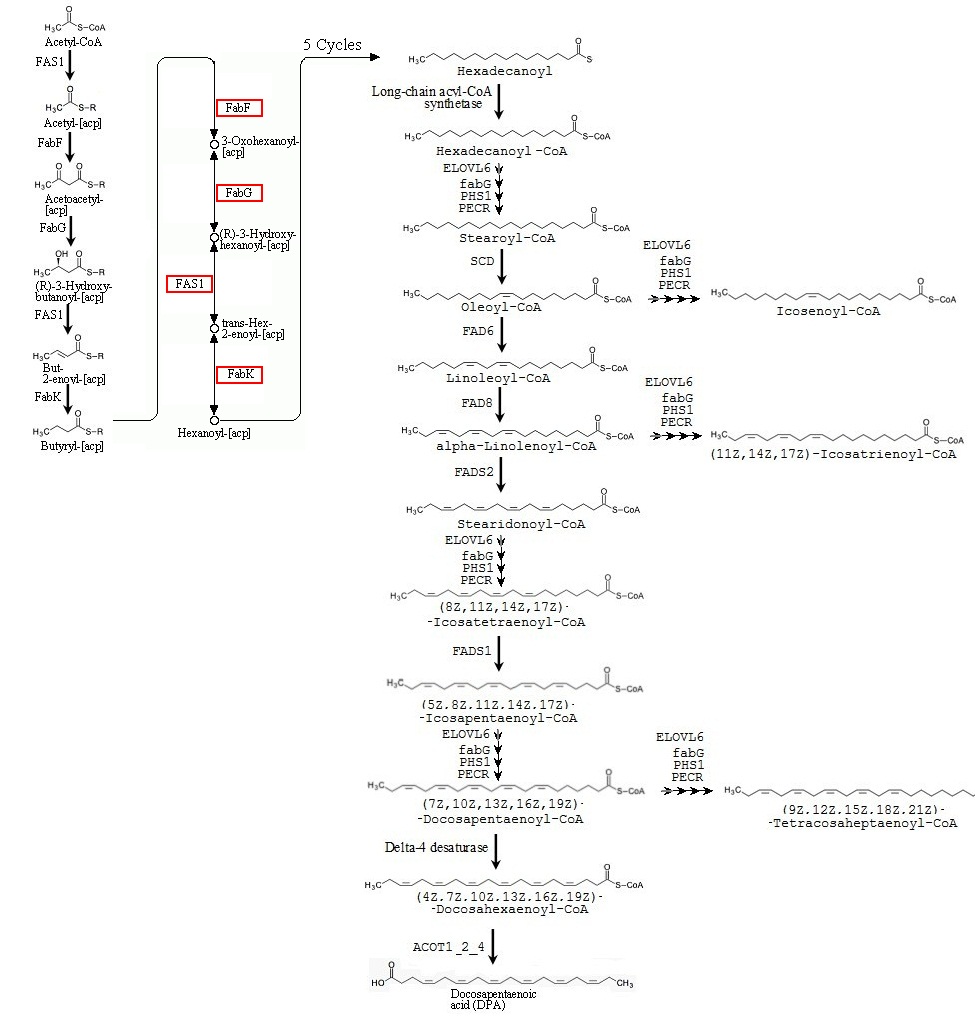
**

**Figure S16: Cordyceps polysaccharide metabolic pathway of *H. sinensis*.**

**
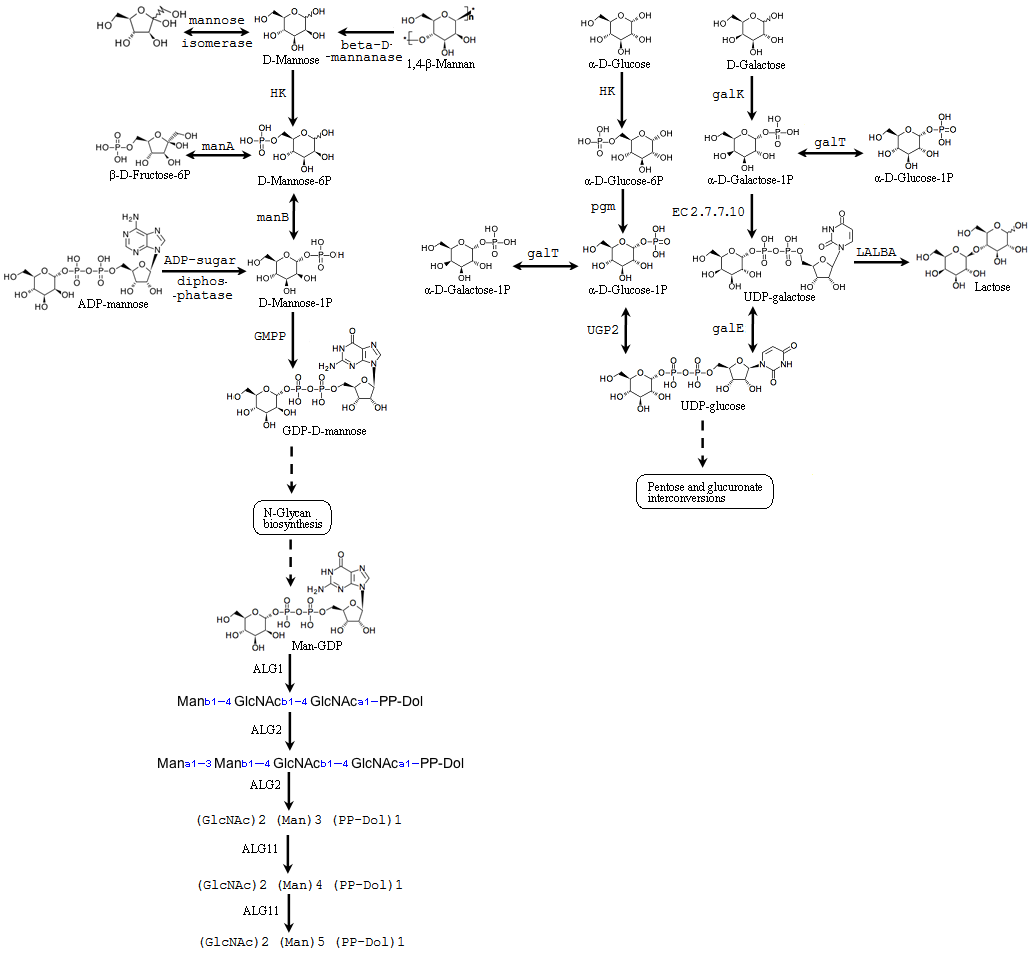
**

**Figure S17:** **Sphingolipid metabolic pathway of *H. sinensis*.**

**
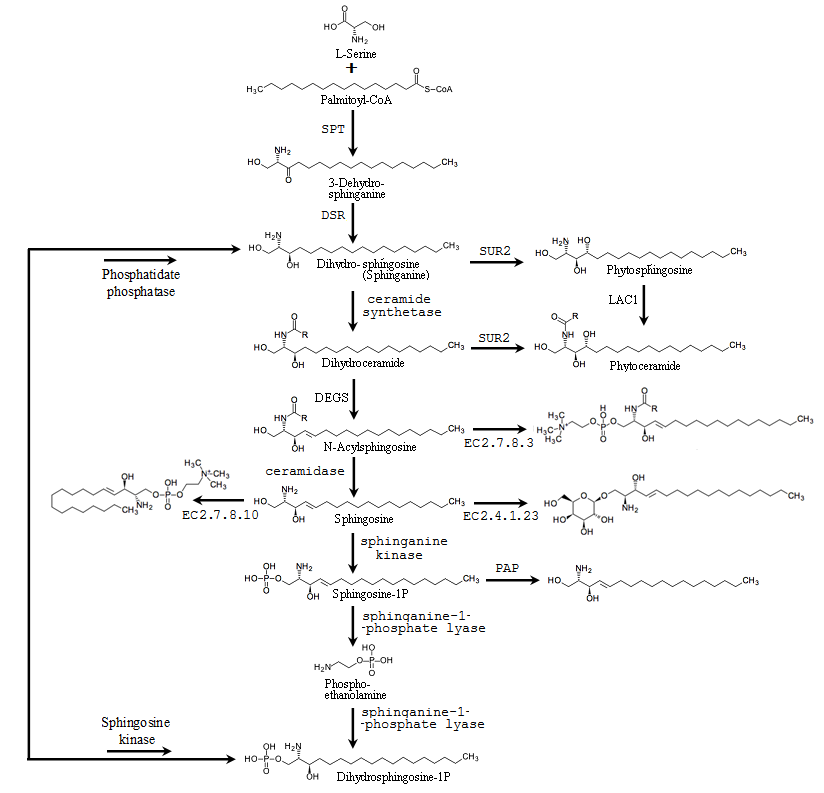
**

**Figure S18: The single colonies morphology photograph of *H. sinensis*.**

The color of single colonies was white, hyphae were fluffy and outward, and the diameter of the colonies ranged from 1 cm to 2 cm, indicating that colonial morphology of *H. sinensis* was similar to anamorph of *O. sinensis*.


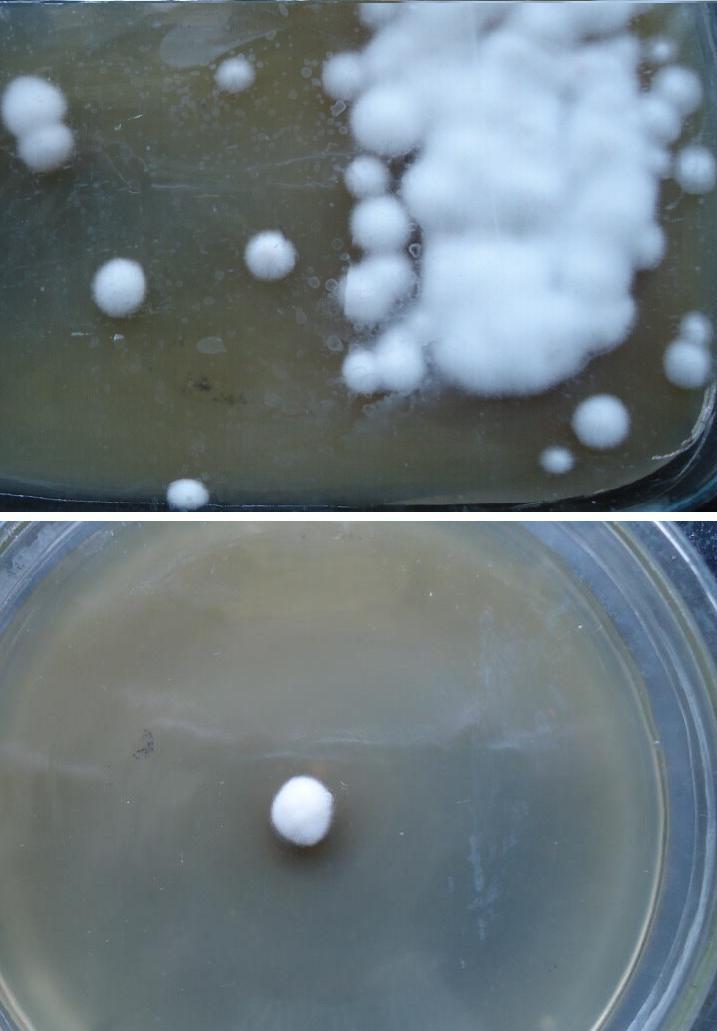


**Figure S19: The SEM photographs of *H. sinensis*.**

The SEM photographs showed that *H. sinensis* exists in the form of mycelia, mycelia present a woven mesh, the diameter of the mycelium ranges from 1 to 2 μm, sporangium can be observed at the edge of the mycelium, *H. sinensis* presents a unique form of fungi.


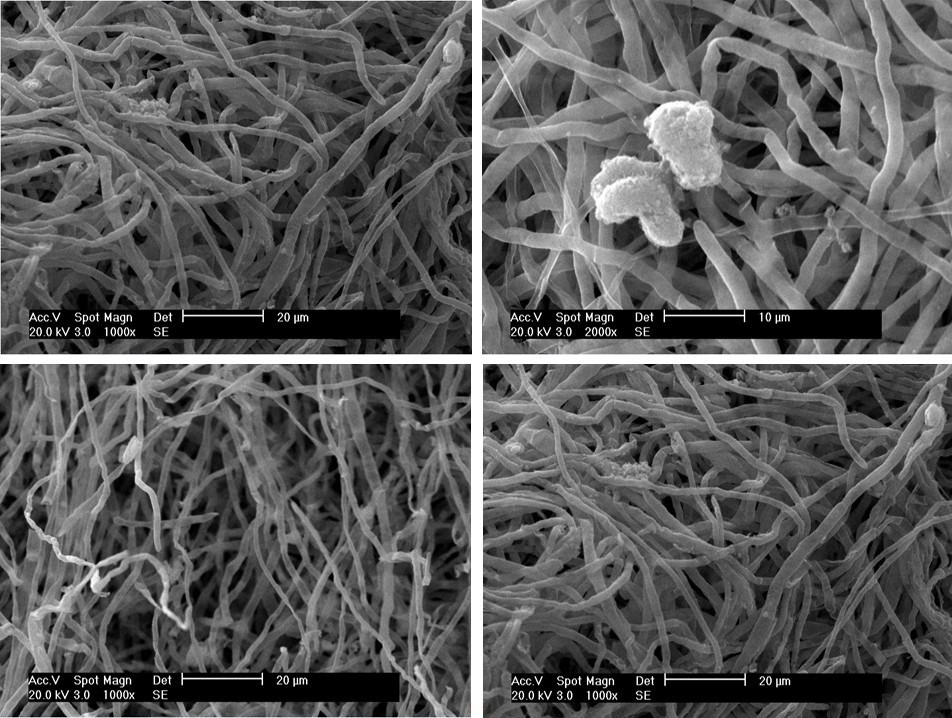


**Figure S20: Phylogenetic analysis of *H. sinensis*.**

Phylogenetic tree showed genetic relationships among *H. sinensis* and other entomogenous fungi based on alignment of the complete 18S rDNA gene sequences. The reliability of the neighbor-joining tree was estimated by bootstrap analysis using 1,000 pseudoreplicate. The marker denotes a measurement of relative phylogenetic distance. The analysis of this phylogenetic tree showed that *H. sinensis* has a close genetic relationship to *O. sinensis*, *H. liboensis*, *Elaphocordyceps capitata* and *H. minnesotensis*.


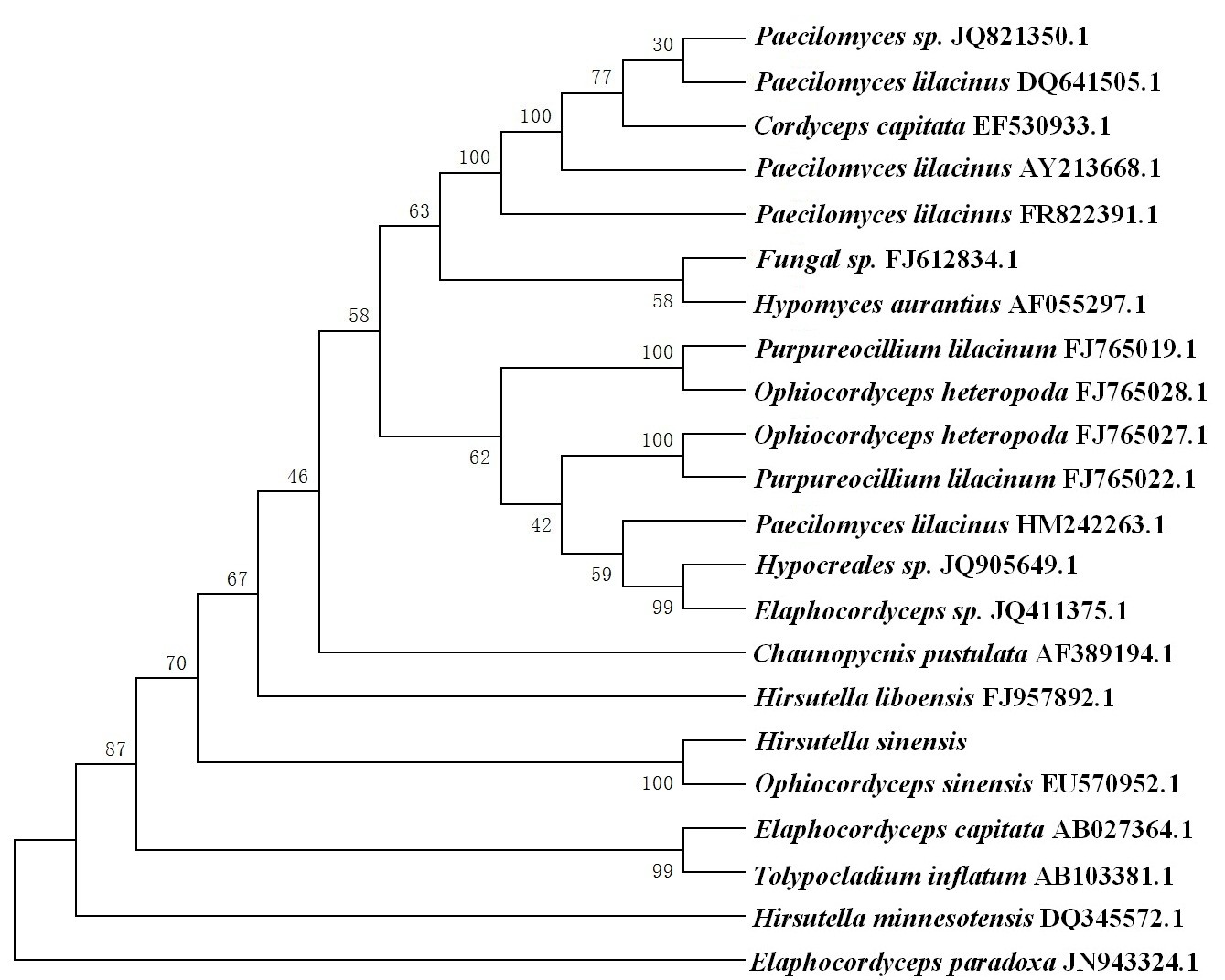


**Table Legends**

**Additional_file_1 as XLS**

**Additional file 1: Table S1** Unigene annotations provide functional annotations of unigene (All) and expression levels. Functional annotations of unigene including protein sequence similarity, KEGG Pathway, COG and Gene Ontology (GO).

**Additional_file_2 as DOC**

**Additional file 2: Table S2** COG function classification of *H. sinensis* unigenes (All) compared with *O. sinensis* grass-part (OSGP) and *O. sinensis* worm-part (OSWP).

**Additional_file_3 as DOC**

**Additional file 3: Table S3** Statistics of *H. sinensis* transcriptome mapped to reference genome and reference gene.

**Additional_file_5 as DOC**

**Additional file 5: Table S4** Biolog metabolic fingerprinting analysis of *H. sinensis*.

**Additional_file_6 as DOC**

**Additional file 6: Table S5** The primers used for cloning and expressing genes involved in mannitol metabolic pathway. **Table S6** The primers used for cloning and expressing genes involved in cordycepin metabolic pathway. **Table S7** The primers used for cloning and expressing genes involved in purine nucleotides metabolic pathway. **Table S8** The primers used for real-time PCR involved in mannitol metabolic pathway. **Table S9** The primers used for real-time PCR involved in cordycepin metabolic pathway. **Table S10** The primers used for real-time PCR involved in purine nucleotides metabolic pathway.

**Additional_file_7 as DOC**

**Additional file 7:** List of 18S rRNA gene, mannitol anabolic functional genes, cordycepin anabolic functional genes and purine nucleotides anabolic functional genes including GenBank accession numbers.

**Supplementary References**

1. Kanehisa M, Araki M, Goto S, Hattori M, Hirakawa M, Itoh M, Katayama T, Kawashima S, Okuda S, Tokimatsu T: **KEGG for linking genomes to life and the environment**. *Nucleic Acids Res* 2008, **36**(suppl 1):D480-D484.

2. Conesa A, Götz S, García Gómez JM, Terol J, Talón M, Robles M: **Blast2GO: a universal tool for annotation, visualization and analysis in functional genomics research**. *Bioinformatics* 2005, **21**(18):3674-3676.

3. Ye J, Fang L, Zheng H, Zhang Y, Chen J, Zhang Z, Wang J, Li S, Li R, Bolund L: **WEGO: a web tool for plotting GO annotations**. *Nucleic Acids Res* 2006, **34**(suppl 2):W293-W297.

4. Mortazavi A, Williams BA, McCue K, Schaeffer L, Wold B: **Mapping and quantifying mammalian transcriptomes by RNA-Seq**. *Nat methods* 2008, **5**(7):621-628.

5. Iseli C, Jongeneel CV, Bucher P: **ESTScan: a program for detecting, evaluating, and reconstructing potential coding regions in EST sequences**. In: *ISMB: 1999*; 1999: 138-148.

6. Audic S, Claverie JM: **The significance of digital gene expression profiles**. *Genome Res* 1997, **7**(10):986-995.
