# Supplementary material for: Transcriptome sequencing and analysis of the entomopathogenic fungus Hirsutella sinensis isolated from Ophiocordyceps sinensis
Source: BMC Genomics. 2015 Feb 21;16(1):106. doi: 10.1186/s12864-015-1269-y (PMC4342880; doi:10.1186/s12864-015-1269-y)
Supplement: Additional file 5: Table S4. — Biolog metabolic fingerprinting analysis of H. sinensis. [file 12864_2015_1269_MOESM5_ESM.doc]

### Additional file 5: Table S4 Biolog metabolic fingerprinting analysis of *H. sinensis*.

| **NO.** | **Carbon substrate** |  | **NO.** | **Carbon substrate** |  |
| --- | --- | --- | --- | --- | --- |
| 0 | Water | − | 48 | D-Ribose | + |
| 1 | Tween 80 | + | 49 | Salicin | + |
| 2 | N-Acetyl-D-Galactosamine | B | 50 | Sedoheptulosan | B |
| 3 | N-Acetyl-ß-D-Glucosamine | B | 51 | D-Sorbitol | − |
| 4 | N-Acetyl-ß-D-Mannosamine | + | 52 | L-Sorbose | + |
| 5 | Adonitol | B | 53 | Stachyose | − |
| 6 | Amygdalin | + | 54 | Sucrose | − |
| 7 | D-Arabinose | + | 55 | D-Tagatose | − |
| 8 | L-Arabinose | + | 56 | D-Trehalose | − |
| 9 | D-Arabitol | + | 57 | Turanose | + |
| 10 | Arbutin | − | 58 | Xylitol | B |
| 11 | D-Cellobiose | − | 59 | D-Xylose | + |
| 12 | a-Cyclodextrin | − | 60 | y-Aminobutyric Acid | − |
| 13 | ß-Cyclodextrin | − | 61 | Bromosuccinic Acid | − |
| 14 | Dextrin | + | 62 | Fumaric Acid | − |
| 15 | i-Erythritol | + | 63 | ß-Hydroxybutyric Acid | − |
| 16 | D-Fructose | + | 64 | y- Hydroxybutyric Acid | − |
| 17 | L-Fucose | − | 65 | p-Hydroxy-phenylacetic Acid | − |
| 18 | D-Galactose | B | 66 | a-Ketoglutaric Acid | − |
| 19 | D-Galacturonic Acid | − | 67 | D-Lactic Acid Methyl Ester | B |
| 20 | Gentiobiose | − | 68 | L-Lactic Acid | B |
| 21 | D-Gluconic Acid | + | 69 | D-Malic Acid | − |
| 22 | D-Glucosamine | − | 70 | L-Malic Acid | + |
| 23 | a-D-Glucose | − | 71 | Quinic Acid | − |
| 24 | a-D-Glucose-1-Phosphate | − | 72 | D-Saccharic Acid | − |
| 25 | Glucuronamide | B | 73 | Sebacic Acid | + |
| 26 | D-Glucuronic Acid | − | 74 | Succinamic Acid | + |
| 27 | Glycerol | + | 75 | Succinic Acid | − |
| 28 | Glycogen | + | 76 | Succinic Acid Mono-Methyl Ester | − |
| 29 | m-Inositol | B | 77 | N-Acetyl-L-Glutamic Acid | B |
| 30 | 2-Keto-D-Gluconic Acid | − | 78 | L-Alaninamide | − |
| 31 | a-D-Lactose | B | 79 | L-Alanine | − |
| 32 | Lactulose | + | 80 | L-Alanyl-Glycine | − |
| 33 | Maltitol | B | 81 | L-Asparagine | − |
| 34 | Maltose | − | 82 | L-Aspartic Acid | B |
| 35 | Maltotriose | − | 83 | L-Glutamic Acid | − |
| 36 | D-Mannitol | B | 84 | Gycyl-L-Glutamic Acid | − |
| 37 | D-Mannose | − | 85 | L-Ornithine | − |
| 38 | D-Melezitose | B | 86 | L-Phenylalanine | − |
| 39 | D-Melibiose | B | 87 | L-Proline | − |
| 40 | a-Methyl-D-Galactoside | − | 88 | L-Pyroglutamic Acid | − |
| 41 | ß-Methyl-D- Galactoside | − | 89 | L-Serine | + |
| 42 | a-Methyl-D-Glucoside | + | 90 | L-Threonine | − |
| 43 | ß-Methyl-D-Glucoside | − | 91 | 2-Aminoethanol | + |
| 44 | Palatinose | − | 92 | Putrescine | + |
| 45 | D-Psicose | B | 93 | Adenosine | + |
| 46 | D-Raffinose | − | 94 | Uridine | − |
| 47 | L-Rhamnose | − | 95 | Adenosine-5’-Monophosphate | − |
| *Notes: +, positive; −, negative; B,borderline* | | | | | |
