# Supplementary material for: Transcriptome sequencing and analysis of the entomopathogenic fungus Hirsutella sinensis isolated from Ophiocordyceps sinensis
Source: BMC Genomics. 2015 Feb 21;16(1):106. doi: 10.1186/s12864-015-1269-y (PMC4342880; doi:10.1186/s12864-015-1269-y)
Supplement: Additional file 6: Table S5. — The primers used for cloning and expressing genes involved in mannitol metabolic pathway. Table S6. The primers used for cloning and expressing genes involved in cordycepin metabolic pathway. Table S7. The primers used for cloning and expressing genes involved in purine nucleotides metabolic pathway. Table S8. The primers used for real-time PCR involved in mannitol metabolic pathway. Table S9. The primers used for real-time PCR involved in cordycepin metabolic pathway. Table S10. The primers used for real-time PCR involved in purine nucleotides metabolic pathway. [file 12864_2015_1269_MOESM6_ESM.doc]

### Additional file 6:

**Table S5** The primers used for cloning and expressing genes involved in mannitol metabolic pathway.

| **Gene** | **Primer** | **Sequence (5’-3’)** |
| --- | --- | --- |
| HK | manA1-F | ATTGAATTCATGTTGGCCGCCATCAGATCTC |
|  | manA1-R | AGTAAGCTTTTACTCGAGGGGGAAGCTCCATG |
|  | manA2-F | ATTGAATTCATGGTGGCAAAGACGGCCC |
|  | manA2-R | AGTAAGCTTCTACCGGTGCCTGTTCCACC |
|  | manA3-F | ATTGAATTCATGTTGCAGCGGATGGGCAAATG |
|  | manA3-R | AGTAAGCTTTTATTCAATTAGGGAGAGGGTGT |
|  | manA4-F | ATTGAATTCATGACCCTCGTCGAGGAGGTGC |
|  | manA4-R | AGTAAGCTTTTAACCGCAGGCGGAGAGGCG |
|  | manA5-F | ATTGAATTCATGGGCAAGGGCTTTGCCC |
|  | manA5-R | AGTAAGCTTTCATGATGCGCGACTCGCATC |
|  | manA6-F | ATTGAATTCATGTGTGTGTCGTCGGGTTG |
|  | manA6-R | AGTAAGCTTTCACCCGAGCGGTGCAAG |
| GPI | manB1-F | ATTGAATTCATGCTGCTGCAGATAGGCCGG |
|  | manB1-R | AGTAAGCTTTTACTCTTCCTCGTCGCCT |
|  | manB2-F | ATTGAATTCATGCTGGCTCCCCTTTCGACTC |
|  | manB2-R | AGTAAGCTTTTACGTCTCCGGGTCCGAGTTC |
|  | manB3-F | ATTGAATTCATGCTGATCCCGACCGACTTCATC |
|  | manB3-R | AGTAAGCTTTTAGGGGCTGCTCGTGGAGCC |
| mtlD | manC1-F | ATTGAATTCATGTTGACAGCAGCTTTCCTCGTC |
|  | manC1-R | AGTAAGCTTTTAGGCGTTTTCGCAGGCAATG |
|  | manC2-F | ATTGAATTCATGCGACGAGGAGGAGCAAGAG |
|  | manC2-R | AGTAAGCTTTTAGCCAGCTCCTTGGACTCTTC |

**Table S6** The primers used for cloning and expressing genes involved in cordycepin metabolic pathway.

| **Gene** | **Primer** | **Sequence (5’-3’)** |
| --- | --- | --- |
| ADPR-PPase | corA-F | ATTGAATTCATGAGCATCCTCCCGCGC |
|  | corA-R | AGTAAGCTTCTACAGCTTCTTTTCCCTCCTCAA |
| PRPS | corB1-F | ATTGAATTCATGGTCCGAAACATTGTCC |
|  | corB1-R | AGTAAGCTTTTAGCCGTGCTGGAAGAG |
|  | corB2-F | ATTGAATTCATGTTGCCTTTCCCAACTTTTC |
|  | corB2-R | AGTAAGCTTTCACTCCCCGTTATGGGTTC |
| purF | corC1-F | ATTGAATTCATGCTGACCAAGACCAGTTGTCTC |
|  | corC1-R | AGTAAGCTTTCACTCGGGCTTCCCGCC |
|  | corC2-F | ATTGAATTCATGCTGCTACGGGTCCATGGC |
|  | corC2-R | AGTAAGCTTTCATTTCGACAAGTGGCGGAG |
| purD | corD-F | ATTGAATTCATGCTGCTCATTAGGTCAGGG |
|  | corD-R | AGTAAGCTTTCACCACGAGCCAAAGTCG |
| GAR TFase | corE-F | ATTGAATTCATGTCGACGAACCAGTCTCTCC |
|  | corE-R | AGTAAGCTTTCATGGTTGGGGGGCAGC |
| purL | corF-F | ATTGAATTCATGCCGCACGAGACACTGGTC |
|  | corF-R | AGTAAGCTTCTACCCCACCCACCTCCGTG |
| purM | corG-F | ATTGAATTCATGCTGCTCATTAGGTCAGGG |
|  | corG-R | AGTAAGCTTTCACCACGAGCCAAAGTCG |
| PAICS | corH-F | ATTGAATTCATGGACCAGTACAACCAG |
|  | corH-R | AGTAAGCTTAGCGGATCGCCAGCAGGG |
| purC | corI-F | ATTGAATTCATGGCTCTCACAACCATCAAC |
|  | corI-R | AGTAAGCTTTTATCTGGTGACGTTTGTATCTTC |
| purB | corJ-F | ATTGAATTCATGCTGCGTGACGGGCTAGAC |
|  | corJ-R | AGTAAGCTTTCACGAGCCAATCTGGCCAG |
| purH | corK-F | ATTGAATTCATGCTGGGTGTACTTGAGGG |
|  | corK-R | AGTAAGCTTTCATTTCTCTCTGCTTCTCGC |
| purA | corL-F | ATTGAATTCATGGGGTCAGTTCAGTTCG |
|  | corL-R | AGTAAGCTTTCATCTATCGGAGACGAGGATG |
| 5'-nucleotidase | corM1-F | ATTGAATTCATGTTGGAAACAGCTCCCCTC |
|  | corM1-R | AGTAAGCTTTCACGAAAGCCGCTCTACTATC |
|  | corM2-F | ATTGAATTCATGGGCAACGACACGACGG |
|  | corM2-R | AGTAAGCTTTCACGTTGCCCGCATCTTG |
| N-glycosylase | corN-F | ATTGAATTCATGGGGCTTGGTTGGGGC |
|  | corN-R | AGTAAGCTTTCAGGGCCTCTGCCGCGTC |

**Table S7** The primers used for cloning and expressing genes involved in purine nucleosides metabolic pathway.

| **Gene** | **Primer** | **Sequence (5’-3’)** |
| --- | --- | --- |
| purine nucleosidase | punA-F | ATTGAATTCATGACCATGCCAGACTCGTC |
|  | punA-R | AGTAAGCTTCTA ACGCGTGCCGTTAGAG |
| adenosine kinase | punB-F | ATTGAATTCATGCTGTCTCACTCGCGGTTAG |
|  | punB-R | ATTAAGCTTCTAACATTCGGCGCACGCG |
| APRT | punC-F | ATTGAATTCATGCTGCCGGCGGCCG |
|  | punC-R | GCCAAGCTTCTACTCGAGTGGCAGATTGACC |
| AMP deaminase | punD1-F | ATTGAGCTCATGGTGAAGACGAACGTGC |
|  | punD1-R | ATTAAGCTTCTAGTTCCGTACCCTGCCAC |
|  | punD2-F | ATTGAATTCATGCTGCTGGCCAAGCTGG |
|  | punD2-R | ATTAAGCTTCTAGCCGGCCTCGAAGAG |
| IMP dehydrogenase | punE1-F | ATTGAATTCATGCTGCATCCGCAATGGC |
|  | punE1-R | ATTAAGCTTCTACGAGGATGAAGCCGTTTTC |
|  | punE2-F | ATTGAATTCATGGTGACCGACCTCACC |
|  | punE2-R | GCCAGACTCTCATGCATACAGCTTCTTCTC |
| GMP synthase | punF1-F | GTTGAATTCATGCTGATCTCCCATCACTCC |
|  | punF1-R | ATTAAGCTTCTACATCACAACGTAGGCGGG |
|  | punF2-F | ATTGAATTCATGCTGCTCCAGAACTTTGCCG |
|  | punF2-R | ATTAAGCTTCTAAGCCGGCAGCCCGCC |
|  | punF3-F | ATTGAATTCATGCTGACTGCCAACCACCTC |
|  | punF3-R | GCCAAGCTTCTAGACGAGCTTGTCAAAGTG |
| guanine deaminase | punG1-F | ATTGAATTCATGAAGCCCGGGAAGAAAAAC |
|  | punG1-R | ATTAAGCTTCTAGGTCTCGGGGACGCG |
|  | punG2-F | GCCGAATTCATGCTGCAAAAGTACACGTTC |
|  | punG2-R | ATTAAGCTTTTACGGGGGGCCCGCGG |
| xanthine dehydrogenase | punH-F | ATTGAATTCATGGCGCCCGTCGCCC |
|  | punH-R | ATTAAGCTTTCATCGTCGCCCCGGCC |

**Table S8** The primers used for real-time PCR involved in mannitol metabolic pathway.

| **Gene** | **Primer** | **Sequence (5’-3’)** |
| --- | --- | --- |
| 18S rRNA | 18S-F | GCAGTGGCATCTCTCAGTC |
|  | 18S-R | TCATCGATGCCAGAACC |
| HK | ManA1-F | CAAGGTATTTGAGGGCCAG |
|  | ManA1-R | CTTCAATCGCCGACAGAAAC |
|  | ManA2-F | GTGTGTCGGGCATGTTTCTC |
|  | ManA2-R | GAAGAGCGAAGTGGCATCGT |
|  | ManA3-F | TTGCCACCTGCGTCTTTACC |
|  | ManA3-R | TGAGTCTTTGGGAAGAGTCG |
|  | ManA4-F | ATCAGGGTCGTTGCCATTG |
|  | ManA4-R | CGATGCGCCGTATCATTGT |
| GPI | ManB1-F | GAGTCCAACGGCAAGTCAATC |
|  | ManB1-R | CAGGGCTCGCCAAAGAG |
|  | ManB2-F | ACACGCTCAACCTCCTCCTT |
|  | ManB2-R | ATTGATCTTGTCGCCGG |
| mtlD | ManC-F | ACCACGGACCGAACAATCAC |
|  | ManC-R | GTGATGAGCTCCGCAATCAG |

**Table S9** The primers used for real-time PCR involved in cordycepin metabolic pathway.

| **Gene** | **Primer** | **Sequence (5’-3’)** |
| --- | --- | --- |
| 18S rRNA | 18S-F | GCAGTGGCATCTCTCAGTC |
|  | 18S-R | TCATCGATGCCAGAACC |
| ADPR-PPase | Cor-A-F | TCGAGGACGGCGAGTTCAT |
|  | CorA-R | CTGTGCCTCAAACTTCTTGC |
| PRPS | CorB1-F | GGCATCTCAATCGCCAAC |
|  | CorB1-R | TCTCGGACAGACTCGCCAAT |
|  | CorB2-F | ATCATCACCGTCGACCTCCAT |
|  | CorB2-R | ATGGGCTCGGCATGAAGATT |
|  | CorB3-F | CCCAGTACCAGGGCTTCTT |
|  | CorB3-R | GTGCTTCACGATGTAGTTTTGC |
| purF | CorC1-F | GTCAAGTCCAAGGTTATTTGC |
|  | CorC1-R | CCAAAGAGGTGGTCGAGTG |
|  | CorC2-F | TCACGGGTGGGACCGATTAT |
|  | CorC2-R | AATGTCGCGCATGATCTTG |
| purD | CorD-F | AGGATGCTCTGAAGCAGATC |
|  | CorD-R | GAGCAAAAGCTCCTCGATG |
| GAR TFase | CorE1-F | GGCCGGTTGGATGTACATT |
|  | CorE1-R | GGTGCAGGTTGATGACTTTG |
|  | CorE2-F | CTCCCAAGAAACTCGACATG |
|  | CorE2-R | GGGTGATGCAGGCTGAG |
| purL | CorF-F | AACGCCGACCTGGACTTTG |
|  | CorF-R | GTGCATGTGTTGATGACCATCTG |
| purM | CorG-F | CGTCATCGAGGAGCTTTTG |
|  | CorG-R | GCGGTAGGGACTTGGTTGT |
| purC | CorI-F | AGTTTCCCGAGCCCATCTAC |
|  | CorI-R | GGCATCGTCGGGATGTATGT |
| purB | CorJ-F | AGTTTCCCGAGCCCATCTAC |
|  | CorJ-R | GGCATCGTCGGGATGTATGT |
| purH | CorK-F | CAGCTTTGGCGACATGATC |
|  | CorK-R | CGTCCGATACCTCCTTGG |
| purA | CorL-F | CCCAACCACCCACATCAAG |
|  | CorL-R | GGCGGCCATCCTCTATGAAT |
| 5'-nucleotidase | CorM1-F | TCTAAAACGGAGCGGTTGCT |
|  | CorM1-R | CCTGGCGTTTGTCGTTGAC |
|  | CorM2-F | CCGCCGTGGATAATGAAAAC |
|  | CorM2-R | ATCCGCACTCTTGTCCTTG |
| N-glycosylase | CorN-F | TCTCGGAGCTGTGCATCG |
|  | CorN-R | GCTCGTCCATTCGTCGTTG |

**Table S10** The primers used for real-time PCR involved in purine nucleosides metabolic pathway.

| **Gene** | **Primer** | **Sequence (5’-3’)** |
| --- | --- | --- |
| 18S rRNA | 18S-F | GCAGTGGCATCTCTCAGTC |
|  | 18S-R | TCATCGATGCCAGAACC |
| purine nucleosidase | PunA-F | ACCCCCGGATCAAGCTACTAG |
|  | PunA-R | GTTGCGCGTCGTGTGTTC |
| adenosine kinase | PunB-F | CTGTCTCACTCGCGGTTAGGT |
|  | PunB-R | CCCAATGGACCACGACAAGT |
| APRT | PunC-F | CACCCGTGGCAATGATGTC |
|  | PunC-R | GGAGTACGGCAAAGACCTCTTC |
| AMP deaminase | PunD-F | CCGGACAGAAGGGAAGAGTTT |
|  | PunD-R | GTCACGTAGCGTTTGAGAACGT |
| IMP dehydrogenase | PunE-F | TGATCTCCCGGTCCGACTT |
|  | PunE-R | TGCTTGCTGTCGGGTAGCT |
| GMP synthase | PunF1-F | GCAAAAACATCTCGGCATCA |
|  | PunF1-R | CGGTCAAGGAACAGCTTGGA |
|  | PunF2-F | AAATGGCCCCCGTAAACG |
|  | PunF2-R | GATGGTGTCGAAAGTGCTGTGA |
| guanine deaminase | PunG-F | CGTGTTTGGGTGGGAGACTT |
|  | PunG-R | ACCTTTTTGGTGTTTCGATCGT |
