# Supplementary material for: Transcriptome sequencing and analysis of the entomopathogenic fungus Hirsutella sinensis isolated from Ophiocordyceps sinensis
Source: BMC Genomics. 2015 Feb 21;16(1):106. doi: 10.1186/s12864-015-1269-y (PMC4342880; doi:10.1186/s12864-015-1269-y)
Supplement: Additional file 7: — List of 18S rRNA gene, mannitol anabolic functional genes, cordycepin anabolic functional genes and purine nucleotides anabolic functional genes including GenBank accession numbers. [file 12864_2015_1269_MOESM7_ESM.doc]

### Additional file 7: List of 18S rRNA gene, mannitol anabolic functional genes, cordycepin anabolic functional genes and purine nucleotides anabolic functional genes including GenBank accession numbers

***Hirsutella sinensis* 18S rRNA (partial), 5.8S rRNA and 25S rRNA (partial) genes and internal transcribed spacers 1 and 2 (ITS1, ITS2), strain L0106.**

**(GenBank accession number, KP090933)**

tccgtaggtgaacctgcggagggatcattatcgagtcaccactcccaaaccccctgcgaacaccacagcagttgcctcggcgggaccgccccggcgccccagggcccggaccagggcgcccgccggaggacccccagaccctcctgtcgcagtggcatctctcagtcaagaagcaagcaaatgaatcaaaactttcaacaacggatctcttggttctggcatcgatgaagaacgcagcgaaatgcgataagtaatgtgaattgcagaattcagtgaaccatcgaatctttgaacgcacattgcgcccgccagcactctggcgggcatgcctgtccgagcgtcatctcaaccctcgagccccccgcctcgcggcggcggggcccggccttgggggtcacggccccgcgccgccccctaaacgcagtggcgaccccgccgcggctcccctgcgcagtagctcgctgagaaccccgcaccgggagcgcggaggcggtcacgccgtgaaaccaccacaccctccagttgacctcggatcaggtagggatacccgctgaacttaagcatatcaataagcggaggaa

**Mannitol** **anaboli****c functional genes from** ***Hirsutella sinensis* strain L0106 (Anamorph *Ophiocordyceps sinensis*).**

***Hirsutella sinensis* strain L0106 hexokinase (HK-1)** **mRNA, partial cds.**

**(GenBank accession number, KP090934)**

**Gene sequence**

ttggccgccatcagatctccaggtttcatccaacccagtactagccgtgacgcttctcgagacgcacacttgctccaacaaggtgcctgtcgatcggcccgtccgcctgctagatgccatctcatcctcgagccatcgcatgcaaccttgtcaccgccccaacctgcctcttcagagcccgtctatttacactccctgtcccccccgcagtcttgtcttgacttccccaggcatcgtgtctttcaagggtcactcccaggcatcgtgtctttcaagggtcattcccaggcaacccctcgactctcgtttacctcatcttcccccaatgtcatgccgtcgctcgtttctgcaatgacgactctgcgaaaagccttcatctccgccatcctcgagagcttgctccgcggcaagtctctgatacaggccatcttgacctattgcatcagccccctcaaggccggccccgcctcctttgcacgcgacactccggccaagagtctccaggagttcctcaaggacgccgaggccgctctgctcggccccgtcagcggccatggcctcctccagctgtccgccggcttgaagaagcagttctttgagcggctgcagacagacacgcaatgcatgctgccttcgtacagccaccagctccccacgggcaacgagtcgggccggtacgtgaccgtcgatgtcggtgggtcgaccttgagagtcgccctggtggaactgcgtggtggagagaagcagggcgagatagtcagcatgcgcaccttccgcatcggcaagctcgccaaggacctcgagggcatggcctttttcgactggatggccgaaaggatacgggaaacgctgtcgacggggctcagcctcgagcccggccccagcaagtcgatacccctggcattggcatggagcttccccctcgag

**Protein sequence**

LAAIRSPGFIQPSTSRDASRDAHLLQQGACRSARPPARCHLILEPSHATLSPPQPASSEPVYLHSLSPPQSCLDFPRHRVFQGSLPGIVSFKGHSQATPRLSFTSSSPNVMPSLVSAMTTLRKAFISAILESLLRGKSLIQAILTYCISPLKAGPASFARDTPAKSLQEFLKDAEAALLGPVSGHGLLQLSAGLKKQFFERLQTDTQCMLPSYSHQLPTGNESGRYVTVDVGGSTLRVALVELRGGEKQGEIVSMRTFRIGKLAKDLEGMAFFDWMAERIRETLSTGLSLEPGPSKSIPLALAWSFPLE

***Hirsutella sinensis* strain L0106 hexokinase (HK-3)** **mRNA, complete cds.**

**(GenBank accession number, KP090935)**

**Gene sequence**

atggtggcaaagacggcccgggagctctttgcctttttggcaaagcagatcgagctgttcctccgtgagcaccacgccgaacacttcgagagccacgtgcgccgtcgcaacacctccagcacgcccatgggctaccgggaggagcacatctttcgtctcggcttcacctttagcttccccgttaagcagctgggtatcaacaagggcaagctcataagatggacaaagggcttcgatatccccgatgccttgggcaaggacgtgtgcgccctgctccaggacgaaatcgaccggttgcgcctccctgtcaaggtggctgctctcgtcaacgacaccgtaggaacgcttatggcgcgctcgtacacgtccatgggaaagcatcgctccatcctcggcggcatctttggcacgggcaccaacggcgcgtacattgagaagacggccaacattcgtaagcccatcgagggccagtacgacacgtcgacgggggaaatggtcgtcaacacggagtggggctcgttcgacaaccagctcaacgtgctgccctcgactccctgggacgaggctctggacgtgcagagcgtgaatcctggggttcaaatgtttgagaagcgtgtgtcgggcatgtttctcggcgagatcgtgcggctggcagttctggacatgatgaagaacgatgccacttcgctcttcaaggacctcaactcgagcttcaacgactgggggaccacgacaaacatcagcccccagtctggtatgttcgaatctgggggcctggacagcgccatcatgtcggtcgcagcctcggacaacagtcccgagctgtccaccctacggcaagagctagagaacacgctccaggtctacaacccgtcgctggaggatgcccaggccttcaaggccgttgccggttccgtggctcgccgcgccgcgaggctctctgccgtcgccatcggcgccgtggctctcaagtctggcaagattgacgatccaaaggaagaaatcatcgacattggcgtcgacggcagcctggtggagcactatcccttctttcgagacatgatctacgaggcgctgcccgcaatcgacggcatcgggcccgagggcgtgaagaagatccgcatcgggattgccaaggacggcagcggcgtgggcgcggccttgatcgcgctcgtcgcccagcggatggagaagccgggcgactttttggcagatctaagaaaggacattaagcgcagcctggacgcgatgccagatcctatcgaggaaccgacagtatcgacgactgtgttggcggtggcgggcgttgtcgggctcgctgcgattgcagccatttggtggaacaggcaccggtag

**Protein sequence**

MVAKTARELFAFLAKQIELFLREHHAEHFESHVRRRNTSSTPMGYREEHIFRLGFTFSFPVKQLGINKGKLIRWTKGFDIPDALGKDVCALLQDEIDRLRLPVKVAALVNDTVGTLMARSYTSMGKHRSILGGIFGTGTNGAYIEKTANIRKPIEGQYDTSTGEMVVNTEWGSFDNQLNVLPSTPWDEALDVQSVNPGVQMFEKRVSGMFLGEIVRLAVLDMMKNDATSLFKDLNSSFNDWGTTTNISPQSGMFESGGLDSAIMSVAASDNSPELSTLRQELENTLQVYNPSLEDAQAFKAVAGSVARRAARLSAVAIGAVALKSGKIDDPKEEIIDIGVDGSLVEHYPFFRDMIYEALPAIDGIGPEGVKKIRIGIAKDGSGVGAALIALVAQRMEKPGDFLADLRKDIKRSLDAMPDPIEEPTVSTTVLAVAGVVGLAAIAAIWWNRHR

***Hirsutella sinensis* strain L0106 hexokinase (HK-4)** **mRNA, partial cds.**

**(GenBank accession number, KP090936)**

**Gene sequence**

ttgcagcggatgggcaaatgcttcttggccgacaagggcctgctcggcgaggacttgggccgcatcgtcaagtcggcctgtgagcactgcggcctcaacgtcgagctgcgggtcattctcaacgactcaagcgcctgcctcctgtcgcgggcctactcgtacacctcgacccgcttcggcctcatcctcggcaccggggtcaacatggccgccttcctccctgtcatgagcatcggcaggcccaagtttggcgtccggcccgacggctggttcgacgaagccagccacgtcattgtcaacacggagctgagcatgtttggacacggcatcctgcccatgacgaggtgggaccgccagctaaccaaggagcaccctcggcctgacttccaacccttggagcacctggtcagcggcatgtacctcggcgagattgttcggcaggccctcgtcgaggccattggtgacaccaggattctgggtggcgtggttccgccatccctcgaggccccttattccttgggcgccgacaccctctccctaattgaaag

**Protein sequence**

LQRMGKCFLADKGLLGEDLGRIVKSACEHCGLNVELRVILNDSSACLLSRAYSYTSTRFGLILGTGVNMAAFLPVMSIGRPKFGVRPDGWFDEASHVIVNTELSMFGHGILPMTRWDRQLTKEHPRPDFQPLEHLVSGMYLGEIVRQALVEAIGDTRILGGVVPPSLEAPYSLGADTLSLIE

***Hirsutella sinensis* strain L0106 hexokinase (HK-5)** **mRNA, partial cds.**

**(GenBank accession number, KP090937)**

**Gene sequence**

atgaccctcgtcgaggaggtgcggaggctggagggactcttcgtcgtcgacaagaagaagcttaaggaaatcaccgaccacttcatgtcggagctggccaagggcttgagcgtcgagggcggcagtatccccatgaacccgacctgggtcatgtccttccccgacggccacgagacgggcacctttctcgccctcgacatgggaggcaccaacctgcgcgtgtgccaaatcaccctgacggaccaaaagtctgagttcgacatcatccagtccaagtaccgcatgcccgaggagctcaaaacgggcaaaagcgacgagctctgggagtatattgccgactgcctgcaccagttcatcgagacgcaccacggcgactgcaacaagatggagaagctgcccctcgggttcacgttttcctaccctgccacccaagactacattgacgcgggcgtgcttcaacgatggaccaagggcttcgacattgccggcgtcgagggcgaaaacattgtgcccatgttcgaggctgcgctggccaagcgtggcgtgcccatcaagctgacggccttgatcaatgacacgacggggaccatgattgcctcggcatacacggacaccaagatgaagattggctgcatctttggaaccggctgcaacgccgcctacatggaggactgcggctcgattcccaagctcgccgacatgaaactgcccgccgacacgcccatggccatcaactgcgagtggggtgcctttgacaacgagcacaaggtgctgccccggaccgactacgacaagattatcgatcgcgactctccccggccgggtcagcaggcattcgagaagatgattgctggtctgtatctgggggagattttccgactggtcatggtggacctttacgacaaccgagacgccaaggtatttgagggccagaacattgacctgttgcgcaagccctacgccttggactcgtcgtttctgtcggcgattgaagaagacccctttgagaacctgcaggagacgcaagacctgttcacaaacaagctcaacattacttgcaaccgcggcgagctggaactcatgcgacgactggccgagctcatcggcacccgcgccgcccgcctctccgcctgcggt

**Protein sequence**

MTLVEEVRRLEGLFVVDKKKLKEITDHFMSELAKGLSVEGGSIPMNPTWVMSFPDGHETGTFLALDMGGTNLRVCQITLTDQKSEFDIIQSKYRMPEELKTGKSDELWEYIADCLHQFIETHHGDCNKMEKLPLGFTFSYPATQDYIDAGVLQRWTKGFDIAGVEGENIVPMFEAALAKRGVPIKLTALINDTTGTMIASAYTDTKMKIGCIFGTGCNAAYMEDCGSIPKLADMKLPADTPMAINCEWGAFDNEHKVLPRTDYDKIIDRDSPRPGQQAFEKMIAGLYLGEIFRLVMVDLYDNRDAKVFEGQNIDLLRKPYALDSSFLSAIEEDPFENLQETQDLFTNKLNITCNRGELELMRRLAELIGTRAARLSACG

***Hirsutella sinensis* strain L0106 hexokinase (HK-6)** **mRNA, complete cds.**

**(GenBank accession number, KP090938)**

**Gene sequence**

atgggcaagggctttgccctcccaccagacggcgagctcggcgcgcgcctccagcagggctacgacgaggcgcgggtggccgcccacctcccccccatcagggtcgttgccattgccaacgactcggtcgcgaccctcgtgtccttcatcttcaactacaatgatacggcgcatcgccaggccgccatgggcctgatcctcggcaccggcagcaacgccacggtcccgctgcggtccaaccgcctccatgccagtaagcggccgcaaaaagtgagcgtcttgcccggcgaggtggtcgacgacgacgttgtcattgccgtcaacaccgagtggagcatcagggggacagcgccgccgatgcgccagctggggctcatcactcggtgggacgacgagctgagcgcgcaaaacgaaaccccgggcttccagccgctcgagtacatgacggcaggccgctacctcggggagctgggccgtatcatgctgcttagctacatgacggagacgctcggcctgcggcaaggagcgctgccgtcggcgctgctcgagacgcacagcctgacgacgacgttcctcggccacttcaagccgccggggccggcggcggccctcgtgtcgaagctgaggacggcttttccggaacgccaggagacggggtttgcttggaccgaacatctggccgaagccctgtaccgcatagccaaggcaatcgagttgcgggcggccggcatcatatcggccggcatcctggccctgctgacgttggccgaggagctgccggccgagggagggagccaaggtccggacgcccgagagctcggggtggggtacacgggcgggtgcatcgtgcacttccaggactacttggcagactgccagaggctggtggacgggctcgtggcgaagcgatatggcgaggagacgcaggtgcgcgtggcgttgagcccttgccacgacggggggataacgggagccggcatactggtggctgcggctctcagtagccaggaagccagagatgcgagtcgcgcatcatga

**Protein sequence**

MGKGFALPPDGELGARLQQGYDEARVAAHLPPIRVVAIANDSVATLVSFIFNYNDTAHRQAAMGLILGTGSNATVPLRSNRLHASKRPQKVSVLPGEVVDDDVVIAVNTEWSIRGTAPPMRQLGLITRWDDELSAQNETPGFQPLEYMTAGRYLGELGRIMLLSYMTETLGLRQGALPSALLETHSLTTTFLGHFKPPGPAAALVSKLRTAFPERQETGFAWTEHLAEALYRIAKAIELRAAGIISAGILALLTLAEELPAEGGSQGPDARELGVGYTGGCIVHFQDYLADCQRLVDGLVAKRYGEETQVRVALSPCHDGGITGAGILVAAALSSQEARDASRAS

***Hirsutella sinensis* strain L0106 hexokinase (HK-8)** **mRNA, complete cds.**

**(GenBank accession number, KP090939)**

**Gene sequence**

atgtgtgtgtcgtcgggttgcgtctgcaccaatcccacagcagaccgagttcaatatcaacatttggaaacacaaaactccaggcacggtgcgacagaccgagtgtcggtccggcgagaacgaaaggcgcccattaaacactcagtcgtcccgctcgatgcacgccagcgcgaccgccgctcccatcagggagctttccctggccgggacaaggcgaatgctcctgggttccgccaggcccttgctctcgaccagctcgtccacgtatcgttggcagttgccgaggtaccccgtatagctttcgatgacgctgccgttgaacgcaacagtggtgctttccaacctcatgtcggcctcggccttgcaccgctcgggtga

**Protein sequence**

MCVSSGCVCTNPTADRVQYQHLETQNSRHGATDRVSVRRERKAPIKHSVVPLDARQRDRRSHQGAFPGRDKANAPGFRQALALDQLVHVSLAVAEVPRIAFDDAAVERNSGAFQPHVGLGLAPLG

***Hirsutella sinensis* strain L0106 glucosephosphate isomerase (GPI-1)** **mRNA, partial cds.**

**(GenBank accession number, KP090940)**

**Gene sequence**

ctgctgcagataggccgggaagcgatgaagatactggtcgaatggggcaacgaggtgagtctgggcctggaaaaagtccgagtaccagacgctgagcagaccgccgagaatgggaatgttgtccttgatgggcgtgttgcggaagtgttggtccatggcatgggcgccgctgaggagcttgtgaaagttgtcgaagccgacgtagatggcgacgctgaggccaatggcgctccagaccgagtagcggccgccgacccagctctcaaagccaaacatgttcttgctgtcgatgccaaacttggtcacctctggctcgttggtggagagggcgacaaagtgcttcgcaatgtcgcccttggcgcccgacttttcgaggaaccacgtcttggccgtgtttgcgttggtcgtcgtctcggccgtggtgaacgtcttggaggcgacgaggaagagcg

**Protein sequence**

LLQIGREAMKILVEWGNEVSLGLEKVRVPDAEQTAENGNVVLDGRVAEVLVHGMGAAEELVKVVEADVDGDAEANGAPDRVAAADPALKAKHVLAVDAKLGHLWLVGGEGDKVLRNVALGARLFEEPRLGRVCVGRRLGRGERLGGDEEE

***Hirsutella sinensis* strain L0106 glucosephosphate isomerase (GPI-2)** **mRNA, partial cds.**

**(GenBank accession number, KP090941)**

**Gene sequence**

ctggctcccctttcgactcgtcaaagcaaaacagctactgaccccgtctctgctcaagtcatccgatcctcctcgcttccaccgacggcaacaatggctcccgcaaacacgctgcctgcctgggcagagcttcaagctcatcgcgacaacgtgggcaagaagtttgtcctcaaggacgcctttgcctcggacaaggaccgcttcaaccgcttctctcgcacctttacctcggacggcgtctcggccgatatcctcttcgactttagcaagaacttcgtcaccgaggacacgctcaacctcctcgtcaagcttgccgagcaggccggcgtcgagcgcaagcgcaatgccatgttttccggcgacaagatcaattttaccgagaaccgcgccgtcttccacacggccctgcgcaacgtcggcggcgtcgagatgaaggtcgacggccaggatgtcatgaactgccccggcggcgtcaacgacgtcctcaagcacatgaaggacttttcggcccaggtccgcagcggcgagtggaagggcttcaccggcaagaagctcaccacaatcatcaacattggcattggtggatccgacctcggccccgtcatggtgaccgaggccctcaagcactacggcgcccacgacatgacgcttcacttcgtctccaacattgacggcacccacatggccgaggccctcaagaactcggacccggagacg

**Protein sequence**

LAPLSTRQSKTATDPVSAQVIRSSSLPPTATMAPANTLPAWAELQAHRDNVGKKFVLKDAFASDKDRFNRFSRTFTSDGVSADILFDFSKNFVTEDTLNLLVKLAEQAGVERKRNAMFSGDKINFTENRAVFHTALRNVGGVEMKVDGQDVMNCPGGVNDVLKHMKDFSAQVRSGEWKGFTGKKLTTIINIGIGGSDLGPVMVTEALKHYGAHDMTLHFVSNIDGTHMAEALKNSDPET

***Hirsutella sinensis* strain L0106 glucosephosphate isomerase (GPI-3)** **mRNA, partial cds.**

**(GenBank accession number, KP090942)**

**Gene sequence**

ctgatcccgaccgacttcatcctcgccgcgcagtcgcacaaccccacgtccaacaaccttcaccagaagatgctcgcctccaactactttgcccaggccgaggccctcatggtcggcaagactgatgacgaggtcaaggccgaggggacggcgagcgatctcgtgccccacaagcgcttcctcggcaaccggccgacgacgtcgatccttgtgggaggctccattgggccggcggagcttggtgccttgattgtctactacgagcacctgacgtttaccgagggcgccatctgggacatcaacagcttcgaccagtggggcgtcgagctgggcaaggtgctggccaagaagatccttaaggagctcgacgagcccggcagcggcgagggccacgacggctccacgagcagcccc

**Protein sequence**

LIPTDFILAAQSHNPTSNNLHQKMLASNYFAQAEALMVGKTDDEVKAEGTASDLVPHKRFLGNRPTTSILVGGSIGPAELGALIVYYEHLTFTEGAIWDINSFDQWGVELGKVLAKKILKELDEPGSGEGHDGSTSSP

***Hirsutella sinensis* strain L0106 mannitol-1-phosphate 5-dehydrogenase (mtlD-1) mRNA, partial cds.**

**(GenBank accession number, KP090943)**

**Gene sequence**

ttgacagcagctttcctcgtccttctcaccatggccaagaaggctgttcattttggcgccggcaacattggccgtggcttcgtcgcctgcttcctgcacaactcgggctacgaagtcgtctttgccgatgtcgtcgactctctcatcgacaggatcaacgccacgccctcgtacaaggtcatcgaggttggcgaagagggcaccacggaccgaacaatcacaaactaccgcgccatcaactccaagacgcacgaggaggacctgattgcggagctcatccctgccgacgttgccacgtgctccgtcggccccaacattctcaagttcattgcccccgtcctcgcaaaggccatcgcccgccgtcctgatggcaaccctctgcacgtcattgcctgcgaaaacgccat

**Protein sequence**

LTAAFLVLLTMAKKAVHFGAGNIGRGFVACFLHNSGYEVVFADVVDSLIDRINATPSYKVIEVGEEGTTDRTITNYRAINSKTHEEDLIAELIPADVATCSVGPNILKFIAPVLAKAIARRPDGNPLHVIACENA

***Hirsutella sinensis* strain L0106 mannitol-1-phosphate 5-dehydrogenase (mtlD-2) mRNA, partial cds.**

**(GenBank accession number, KP090944)**

**Gene sequence**

cgacgaggaggagcaagaggcgtacgtgaacaagatcatcaagcgcatcgccaacccccacctggaagacgccggcgagcgcgtcggccgcgcccccttgcgcaagccctcgcgcaaggagcgcttcattggccccgccgccgagcttgccgaaaaggaccagagcatcaagttcctcctcgatgccgtcgagatgtgcttccgcttccaaaacgtcgacgacgacgaagagtccaaggagctggc

**Protein sequence**

RRGGARGVREQDHQAHRQPPPGRRRRARRPRPLAQALAQGALHWPRRRACRKGPEHQVPPRCRRDVLPLPKRRRRRRVQGAG

**Cordycepin anabolic functional genes from *Hirsutella sinensis* strain L0106 (Anamorph *Ophiocordyceps sinensis*).**

***Hirsutella sinensis* strain L0106 ADP-ribose pyrophosphatase** **(ADPR-PPase) mRNA, complete cds.**

**(GenBank accession number, KP090945)**

**Gene sequence**

atgagcatcctcccgcgccgcctgctcactgccttcagtcaaactcgtcctctcgtgtccctcgcgcactcgccgcagcgcagcctcatgacgtcttctagctcggccgcgtggctggagcaaatccccatcgacctgccccatggcatctcccggcatcagctacagtcctttaggccgttcaacaactgggctttgaccctgcataaatccctcggcctccagacaaactcgtcgcaccccttccacgccgagccctacgccctccgctccatcaccatccagtcctacgaccgcttcggcggcgaccgcctcggcttcgtcaagctgaccgcctccgtctccaacgccgccggcgagtccctccccgccgccaccctcctgcgcggcccctcggtgggcatgctcgtcatgctcatacccgacgacgcgccctccggctccgacgagcgctacgtcgtcttgacggttcagccgcgcatccccgccgccagcctgagcttcgccgagctgccggccggcatggtggacgacgcgggcagcttcaagggcgcggctgccaaggaaattcaggaggagctgggcatcaccatccacgaggacgacttgacgtgcctgagcgatatggccggccccgcccctcaagacgtcgacgagggtctcgcggctgccatgtttcccagcgcgggcggctgcgatgagcacgtcaccctctacagccacgagcggcgcatccccagggaccagctacgcgagtggagcgggcggctgacggggctgcggtcccagggcgagaagattaccctcagggttgtgcccatgaaggacctctggagagagggggcacgcgacgccaaggcactgggggcgctcgccctgtgggaggggttgaggagggaaaagaagctgtag

**Protein sequence**

MSILPRRLLTAFSQTRPLVSLAHSPQRSLMTSSSSAAWLEQIPIDLPHGISRHQLQSFRPFNNWALTLHKSLGLQTNSSHPFHAEPYALRSITIQSYDRFGGDRLGFVKLTASVSNAAGESLPAATLLRGPSVGMLVMLIPDDAPSGSDERYVVLTVQPRIPAASLSFAELPAGMVDDAGSFKGAAAKEIQEELGITIHEDDLTCLSDMAGPAPQDVDEGLAAAMFPSAGGCDEHVTLYSHERRIPRDQLREWSGRLTGLRSQGEKITLRVVPMKDLWREGARDAKALGALALWEGLRREKKL

***Hirsutella sinensis* strain L0106 ribose-phosphate pyrophosphokinase** **(PRPS-1) mRNA, complete cds.**

**(GenBank accession number, KP090946)**

**Gene sequence**

atggtccgaaacattgtcctgctcggcggcaactcgcatccgcacctgacggagagcgtctgcaacatcctctgcatccccccgggcgaccgcatcctcaccaagttctcgggtggcgagagccgttgcgaaatcaaggactcggtccgcggcaaggatgtctacatcatccagtctggcgcgggctgcgtcaacgacaacatcatcgacctctgcatcatgatctcggcctgcaagacgggctcggcaaagcgcgtcaacgccgtcatccccctcttcccctactcgcgccaacccgactggccctacaacaagatgggcgccccgctgtccaaacccgccgaggcccagtccgcaaaggactacacctttgagagcgtgccggccacgcctcaccacggcgttccccgcaccaacggcctcggcattgcaaacggcgcccacagcctcgccgagaagctgtccaagagcgtcatctccggagacgcccgagccgagtccaacggtgcagtccacctgcccaagcgctccgaccacatgtccggcgtcggctccaacggccgcgcccatcacaaaggcgacgcccccgtctcctcgctcaccaacttcaccacgcacgactacgagaaccccagcagcatctccgccttgcagcccaagcccggctacaagcagtgggtcgcgcaggcgggcacgctcattgccgatctcctcacctgcgccggcgccgaccacatcatcaccatggacctccacgacccccagtaccagggcttcttcgacgtgcccgtcgacaacctctacgggaagcccctgctgcaaaactacatcgtgaagcacattcccgactacaaggaggccgtcgtcgtctctcccgacgctggcggggcaaagcgagcctcggccattgccgacgacctcgacatgaagtttgctctcatccacaaggaacgccgtcccatcaagttcaacgagcaacgcaatgccagcatgatgctcgtcggcgacattaccgaccgcgtctgcatcctcgtcgacgatcttgccgataccgcaaacactgttactcgtgccgccaagctgctcaagcgcgtgggcgccacgcgcgtctatgccctcctcacccacggcatcttcagcggagatgccattccgcgccttaatgcctcggccattgacaagatcatagtcaccaactcggtgcctcaggaggagcatcgccggctgtgccccaagttggaggttttggacatagcccccgtcttcgccgaggccatccgccgcgtccaccacggcgagtccatcagcaggctcttccagcacggctaa

**Protein sequence**

MVRNIVLLGGNSHPHLTESVCNILCIPPGDRILTKFSGGESRCEIKDSVRGKDVYIIQSGAGCVNDNIIDLCIMISACKTGSAKRVNAVIPLFPYSRQPDWPYNKMGAPLSKPAEAQSAKDYTFESVPATPHHGVPRTNGLGIANGAHSLAEKLSKSVISGDARAESNGAVHLPKRSDHMSGVGSNGRAHHKGDAPVSSLTNFTTHDYENPSSISALQPKPGYKQWVAQAGTLIADLLTCAGADHIITMDLHDPQYQGFFDVPVDNLYGKPLLQNYIVKHIPDYKEAVVVSPDAGGAKRASAIADDLDMKFALIHKERRPIKFNEQRNASMMLVGDITDRVCILVDDLADTANTVTRAAKLLKRVGATRVYALLTHGIFSGDAIPRLNASAIDKIIVTNSVPQEEHRRLCPKLEVLDIAPVFAEAIRRVHHGESISRLFQHG

***Hirsutella sinensis* strain L0106 ribose-phosphate pyrophosphokinase** **(PRPS-2) mRNA, partial cds.**

**(GenBank accession number, KP090947)**

**Gene sequence**

ttgcctttcccaacttttcctcatctcccattctcaactttatcccccattgcctcgaccttgtgtctgcaaaaaaatctcccccacgaacccgactttcttctcacacaatcgcccgcggcaatgttggatcagatggcgaatgaaatcaagcttatctcggggagcggtcacccggagctgagtggccagattgccacccggctgggcatctcaatcgccaacacgatgagcctcaattactccaatcaggaaacgagtgtttccattggcgagtctgtccgagacgaggacgtctttatcctgcagtccaccgcaccgggagacgtcaatgacgggctcatggagctgctcatcatgattcacgcttgcaagacagcctcggctcggcgcatcaccgccgtcatccccaacttcccctacgcccggcaggacaagaaggacaagtctcgcgctcccatcagcgctaggctcattgcaaacatgctgcaggtttccggttgcaaccatgtcatcaccatggacttgcacgcctctcaaattcagggcttcttcaatgttcccgtggacaacctctacgctgagccgtctgttctgcgttggatcgaacagaaccttgacgtggagaattgcgtcatcgtctctcccgacgccggcggtgccaagcgcgcaacctccattgccgatcgcctgaacacagcctttgccctgatccacaaggagcgcccccgtcctaatgtcgtggggcgcatggttcttgttggcgatgtccaggacaaggtggccattcttgtcgatgacatggctgatacctgcggaacactcgtcaaggcggcccagactgtcaacgagcatggcgcccgcaaggtctatgccgttgtcacgcacggcatcctgagcggcagcgccatcgataacatcaaccggtcgtgcctctccggcctggtcgtgaccaacactgttcctctcggtgacaagcccgaccgatgccctaagctcaaggtcatcgacgtgtctggtaccctagctgaggcgattcgccgaacccataacggggag

**Protein sequence**

LPFPTFPHLPFSTLSPIASTLCLQKNLPHEPDFLLTQSPAAMLDQMANEIKLISGSGHPELSGQIATRLGISIANTMSLNYSNQETSVSIGESVRDEDVFILQSTAPGDVNDGLMELLIMIHACKTASARRITAVIPNFPYARQDKKDKSRAPISARLIANMLQVSGCNHVITMDLHASQIQGFFNVPVDNLYAEPSVLRWIEQNLDVENCVIVSPDAGGAKRATSIADRLNTAFALIHKERPRPNVVGRMVLVGDVQDKVAILVDDMADTCGTLVKAAQTVNEHGARKVYAVVTHGILSGSAIDNINRSCLSGLVVTNTVPLGDKPDRCPKLKVIDVSGTLAEAIRRTHNGE

***Hirsutella sinensis* strain L0106 amidophosphoribosyltransferase (purF-2) mRNA, partial cds.**

**(GenBank accession number, KP090948)**

**Gene sequence**

ctgaccaagaccagttgtctctcactcgcctttgcatcttcagtcgactgcgccgactcttgttctcatccggcgcctcggcagagtcctcggcagagtccttcgccaccccccaacgtctccactaaaggcccgtcgcatcggcacccaacgcgcactcgttcgcccttgaagctcgtctcgtcggcagaaatgtgcggcatcgtcgccctcatattggccgacaccgactcgcacaacgccgacgcggctgtcgatctgcatgaaagtctctactatctccaacatagaggccaagatgccgcgggcatcaccacctgcggcgcgggcggcagaatcttccagtgcaagggcaacggcatggtagccaaggtgtttggggatggcaagcgcgtcgccgacctacctggatacatgggcatcgcccatcttcgctacccaaccgcgggaacgagctcgagcgcagagtcgcaaccgttctacgtcaactcgccctacggaatatgttgcgctcacaacggaaacatcaccaacgcccccgagctgcgccgtttcctcgaccaagaggcgcaccgccatgttaacacggattccgatagcgaactgatgctcaacatctttgccaacgccctcaacgagactggcaaggcccgggtcaacgtcgacgacatcttcaaggccctgaccaagatgtatcagatatgcgagggcgcgtgggcaaacacagccatgattgcaggcttcggcatttttgctttccgagactcgtttggtatccggcccttggtcatgggcagtcggccctcggcgacggtcacgggtgggaccgattatatgttggcgtcggagagcatcgcgcttcggcaagttggcttcaagatcatgcgcgacattctccccggcgaggcagtctttatccaaaagggcgggaagcccgag

**Protein sequence**

LTKTSCLSLAFASSVDCADSCSHPAPRQSPRQSPSPPPNVSTKGPSHRHPTRTRSPLKLVSSAEMCGIVALILADTDSHNADAAVDLHESLYYLQHRGQDAAGITTCGAGGRIFQCKGNGMVAKVFGDGKRVADLPGYMGIAHLRYPTAGTSSSAESQPFYVNSPYGICCAHNGNITNAPELRRFLDQEAHRHVNTDSDSELMLNIFANALNETGKARVNVDDIFKALTKMYQICEGAWANTAMIAGFGIFAFRDSFGIRPLVMGSRPSATVTGGTDYMLASESIALRQVGFKIMRDILPGEAVFIQKGGKPE

***Hirsutella sinensis* strain L0106 amidophosphoribosyltransferase (purF-3) mRNA, partial cds.**

**(GenBank accession number, KP090949)**

**Gene sequence**

ctgctacgggtccatggccaggttgtggagaccgacatcctcccggcgctcggggcttcggggctccgtgcccggtgccagcgaggccccgtcatgtcgtccgttgacggcgacgggcgagtcgtcggcggctgtgtgggcgagcccactgctcccgttggtgctcggccgccgggcactcgcaaatgccgccgacttcaacttcttcttgcgtattcgatcgaggtgctccaggtagccgtcgggaaggcccgtcttgtactcgccgcagaagacgcccacctcaaagtccttgatcaggccctggggcgaggactctgtgcaggcagccttcagatcctcgaggtcctggaatatcacgtcgtctgcgttgatgtgcttggcaatgtcctgtctcgtcttgccgtgcgcgatgagctcgttggggctggcgaggtcaatgccgtgaatgtgcggatacgtgatgggaggcgcgcagctcgcaaagatgaccttgcgggccccagcctccctcgccatgctgacaatctctctgcttgtcgtgccgcgaacgatggagtcatcaaccaagcatacaacacgtccctggaactcggattccatggcgctcagcttcctccgaacgcccttttcgcgcgccttttgcccgggcaggataaaggtcctgtacacgtagcggttcttgacaaatccgttggagaagggcttgccgagctgttccgacacaatggcggcggcagtattgctcgtctctggcaccgggatgaagacatcgatttccttgatgccgtcttcgccgagggtctccttgagcttcttggccagcttgacgcccatattttgtctactcgtgtggatcgagatgccgtccatgacagtatcgggtcttgcatagtagacgtactcaaagatgtcaggcgagtatgcctggcgctccgccacttgtcgaaa

**Protein sequence**

LLRVHGQVVETDILPALGASGLRARCQRGPVMSSVDGDGRVVGGCVGEPTAPVGARPPGTRKCRRLQLLLAYSIEVLQVAVGKARLVLAAEDAHLKVLDQALGRGLCAGSLQILEVLEYHVVCVDVLGNVLSRLAVRDELVGAGEVNAVNVRIRDGRRAARKDDLAGPSLPRHADNLSACRAANDGVINQAYNTSLELGFHGAQLPPNALFARLLPGQDKGPVHVAVLDKSVGEGLAELFRHNGGGSIARLWHRDEDIDFLDAVFAEGLLELLGQLDAHILSTRVDRDAVHDSIGSCIVDVLKDVRRVCLALRHLSK

***Hirsutella sinensis* strain L0106 phosphoribosylamine-glycine ligase (purD-1) mRNA, partial cds.**

**(GenBank accession number, KP090950)**

**Gene sequence**

ctgctcattaggtcagggccgcaggactactattctaacaacaatcccctgccgctgccgaccgaactgatcgacagactcgcagatacagcgaacatgaccgatctccgcgttctgctcattggcggtggcggccgcgaacatgcgcttgcatggaagctgagccagtcgccccgagtcaattgcattctggtagtgcccggcaatggtggcaccgcgtcatgcccgaagacggcaaacgtgacctccgttgcggccgatgatttttcagggctcgttgatttctcaaggtcaaatggcatcaatctggtcgtcccgggccccgaagcgctactcgtcaacggggtagagggcttcttcaagagggccggcataccgttcttcggcccgtccaaggaggcggcgcggctggaaggcagcaagacgtactcgaaagattttatgatgaaataccagattcccacggccgcatacgaaaacttttccgactacgacaaggccatgcgctacctcgacagggttagtcacgacatcgtcatcaaggcgacaggcttagcggccgggaaaggcgtcattctcccccagacgaaagaagaggccaaggatgctctgaagcagatcatggtggacaaggctttcggtgctgctggcgacgaggtcgtcatcgaggagcttttgctcggggaagagctcagtgtcctcacattttgcgacggatatacaaccaagtccctaccgctcgctcaagaccacaagaggatattcgatggagatcagggccccaacacaggcggcatgggttgctatgcgcctactaacattgccacgcgggagctgatggaaataatcgacgcggaaatagtcaagcccaccatatcaggcatgcgtcgagagaggcatcccttccgaggcatcctgtttaccggcctcatggtcacgagcgcgggacccaaggtcctcgagtacaatgtgcgattcggtgaccccgaaactcagaccgttctgccactgctgtcaggcgacaccgatctggccgacatcatgctcgcctgcgccgagggatacttagacgactgccatctcaagatcgacaacaagttcagcgccaccgttgtcctggccgcccgaggttaccctggctcatacgccaaaggcacttcgatgaccgttcgggatgcttctaaaggaagcacaatcttccacgccggcaccaatctgagcgaaggccagctgacgacatctgggggccgtgtcattacaatcaactcggtcggcgattcgctgagggacgctgtcgatgcgtgttacgccgccctctcctctggagtaattgagttcgaggggatgttcttccgaaaggacattgcacatcgggccctcagagaatcgagcagggtcgccctgacctacgcccaggctggcgtggacatccaagcaggaaacgacttcgtggaaaaaatcaagcaggcggttgccagcaccaagcgacctggcgctgacgccgaaattggcggtttcggtggagagttggacctctcccagtgtggataccaagacgcgcccatcttggtcgccgctatcgacggcgtggggacgaaactcatgattgcgcagtcgatgaagcggcacgataccgtgggcattgatctagtggccatgaatgtcaacgacttgattgtccagggggccacgccgctcatgttcctcgactactacggctgcagtaaactcgacttggcctcggcttcctcgtttgttcggggcgtcgccgatggctgccgccaggccggatgcgccctggttggcggggaaacggcagagatgccgggcatgtatcagggcgaggactacgacgcagccggttgcgcgattggcgccgtcgtcgcctctagccgcctcccacgcaaagacaccatggttgaaggggacgtgctcttgggcttggcatctcacggcgtccactccaacggcttctccctcgtgagacgcattgtgcagagagccggtctcgactacaactccaaggcgccctgggactcatgccggactgtcggcgaatcgctgctaacgcccacccgcatctacgttacgagccttctccctgtccttgcccagataaagggcctggcacacatcaccggaggcggcctcatcgagaacgtgccgcgcatgcttcccgaggctcgcctcgctgccgacatcgactttggctcgtgg

**Protein sequence**

LLIRSGPQDYYSNNNPLPLPTELIDRLADTANMTDLRVLLIGGGGREHALAWKLSQSPRVNCILVVPGNGGTASCPKTANVTSVAADDFSGLVDFSRSNGINLVVPGPEALLVNGVEGFFKRAGIPFFGPSKEAARLEGSKTYSKDFMMKYQIPTAAYENFSDYDKAMRYLDRVSHDIVIKATGLAAGKGVILPQTKEEAKDALKQIMVDKAFGAAGDEVVIEELLLGEELSVLTFCDGYTTKSLPLAQDHKRIFDGDQGPNTGGMGCYAPTNIATRELMEIIDAEIVKPTISGMRRERHPFRGILFTGLMVTSAGPKVLEYNVRFGDPETQTVLPLLSGDTDLADIMLACAEGYLDDCHLKIDNKFSATVVLAARGYPGSYAKGTSMTVRDASKGSTIFHAGTNLSEGQLTTSGGRVITINSVGDSLRDAVDACYAALSSGVIEFEGMFFRKDIAHRALRESSRVALTYAQAGVDIQAGNDFVEKIKQAVASTKRPGADAEIGGFGGELDLSQCGYQDAPILVAAIDGVGTKLMIAQSMKRHDTVGIDLVAMNVNDLIVQGATPLMFLDYYGCSKLDLASASSFVRGVADGCRQAGCALVGGETAEMPGMYQGEDYDAAGCAIGAVVASSRLPRKDTMVEGDVLLGLASHGVHSNGFSLVRRIVQRAGLDYNSKAPWDSCRTVGESLLTPTRIYVTSLLPVLAQIKGLAHITGGGLIENVPRMLPEARLAADIDFGSW

***Hirsutella sinensis* strain L0106 phosphoribosylglycinamide formyltransferase (GAR TFase -1) mRNA, complete cds.**

**(GenBank accession number, KP090951)**

**Gene sequence**

atgtcgacgaaccagtctctccactcttgcccccggaacgacgccatcggtgtggcggtgtcgctgatggggaaagaacccgtcagggtcgatgtcccctgccgcagacccggcgacgatgtcacggtcagcgacgacggacccgtcttccgcgcgactctcaaggctttggagcagaaaacgggcaacatgcgcacgcagatgaaaaaggtcctgaagcgagcagaacaggctcacctgtctcagatcgaagccaacgatgcattcgttgcctttatggaggccctgcgcgaggcctccacgacaaacgctaacgcggtgcagccggctctggagcattactttgacaatattgcccgcgaagttctgacttatgagcggcaaaacactgtcaacctgcaaaggatcatcattgaacccgtcaacaagctttaccagttcgacattaaacaggcggaatccaagaagcgagatttcgaggaggagagcaaagattactacgcctacgtgtctcgttaccttggccagcgtcaagactcggtcaaggctaagaaacttgccgaaagcgactccaagtaccagaacaagcggcgcaactttgagctcaagagatttgactattctagcttcatgcaggacctccacggcggccgcaaggaccaggaggtcttgtctcatctgacgaaatacgctgatgcgcaaacaaagggcttcctcgccactgccaagaagattgaagggttcttaccacagctcgaggccctctcgagcgaagtccaggacgccgacaaggaatatcagtatcgtcgccgcgaacgcgaggagaagcggcgtgtcttggagaagagcaatacgccctacatggagcccgagcaagcgagcatcggctcttcaatccccattgcttcgacagcgaatggaactgccggcaacgtctctgattccgagctcggtcgcgcagacagcacgggatcccaagttaagaacgccatctctatcgggggtggccagacgaatgtcggccacggcgtggaactctcgaggtctcctggcagcttggcgcagcctgtcgttggcagccccgtacaagcgtccaagttcaaggggattcgcgacttggaggagcgagactcggcacaagcggcggtcatccagcgaaaagaaggattactctgggcgctcaacaggccgggcgggcatgtggacccgcgcacattgaacaagcaaggctggcataagttctggattgtcctggatcaaggtaagctgtcggaatacagcaactggaaacagaggctcgatcttcacatggaccccatcgacctccgaatggcgtcggttcgcgaggcgcgaaacgctgagcgtaggttctgcttcgaggtcatcacgccaagcttcaagcgcgtgtatcaggcgacgtcggaggaagatatgaacagctggatcgtctcgatcaacaacgccctgcaaagcgccatggaggggagggcggttcaagaaaaggttggctccggcgcaaagagtcccggagaatcgtccgtcaagcgcgacattggctcgattctaacaggcaagacgcagtcactcagccacggcgcccatcacccgcaccatcattctagcgctggaagcggcatgccattgcggcgaatcacggttggtgcgagaccgacaatgattcggacgcccagttcgggctacgaggaaaagcccgacaagttgcttcagatggttcgagacgccgaccagggcaactactggtgcgccgactgcggatccggatccaaggtcgaatgggtgtccatcaacctgggcatcgtgctctgcatcgaatgcagcggcatccatcggtcgctgggcacgcatatcagcaaagtgcggtctctcacgctggatatcaagtcgtttaccatcgatatcgtcgaagtgcttctgctcattggaaaccgggtctccaacatggtctgggaggccaaggtcgaaatgcccaacaagccgagcgcacaggccacgcgcgagcagcgactccgattcatcactgccaagtacgtggagagggcattcgtcgaacccatctcgtcgacgctgtcgcggtacggcacggcagacgagacgttgctagccgccattaagaagaacgagattcagcaagtgttgtacgccctggcactgagagcgaaccccaacgtggtggacaagatgagagggacacaccccatctggctcgccgctggcggcggcggatccggcctcgccgtcgccgacgccaggcgtgggcgcgtcggaccaggaggtcaaggcagtgccgttccccgtggcggagcttctcatccagaacggggccgagatcccgacaatgctgcccgcgttcccgctggggcggtctgcacagcagtactacgaacaaaagctcgggaaaagcaacgcgggctcgacggacggaatgccgccgctgccccccaaccatga

**Protein sequence**

MSTNQSLHSCPRNDAIGVAVSLMGKEPVRVDVPCRRPGDDVTVSDDGPVFRATLKALEQKTGNMRTQMKKVLKRAEQAHLSQIEANDAFVAFMEALREASTTNANAVQPALEHYFDNIAREVLTYERQNTVNLQRIIIEPVNKLYQFDIKQAESKKRDFEEESKDYYAYVSRYLGQRQDSVKAKKLAESDSKYQNKRRNFELKRFDYSSFMQDLHGGRKDQEVLSHLTKYADAQTKGFLATAKKIEGFLPQLEALSSEVQDADKEYQYRRREREEKRRVLEKSNTPYMEPEQASIGSSIPIASTANGTAGNVSDSELGRADSTGSQVKNAISIGGGQTNVGHGVELSRSPGSLAQPVVGSPVQASKFKGIRDLEERDSAQAAVIQRKEGLLWALNRPGGHVDPRTLNKQGWHKFWIVLDQGKLSEYSNWKQRLDLHMDPIDLRMASVREARNAERRFCFEVITPSFKRVYQATSEEDMNSWIVSINNALQSAMEGRAVQEKVGSGAKSPGESSVKRDIGSILTGKTQSLSHGAHHPHHHSSAGSGMPLRRITVGARPTMIRTPSSGYEEKPDKLLQMVRDADQGNYWCADCGSGSKVEWVSINLGIVLCIECSGIHRSLGTHISKVRSLTLDIKSFTIDIVEVLLLIGNRVSNMVWEAKVEMPNKPSAQATREQRLRFITAKYVERAFVEPISSTLSRYGTADETLLAAIKKNEIQQVLYALALRANPNVVDKMRGTHPIWLAAGGGGSGLAVADARRGRVGPGGQGSAVPRGGASHPERGRDPDNAARVPAGAVCTAVLRTKAREKQRGLDGRNAAAAPQP

***Hirsutella sinensis* strain L0106 phosphoribosylformylglycinamidine synthase (purL-1) mRNA, complete cds.**

**(GenBank accession number, KP090952)**

**Gene sequence**

atgccgcacgagacactggtcggtggctcgtgctacaccgccccgaccgcgcaggagctcaaagcccgcgccaacaagaccagccagcccaagatcaaggacatccgcggccaatgggtctattatgtcgacctgcggcgcgcagacgcaaccgtcgtcggccaggtcaaggagctgctgcacgacgtcgactcacagccgctgctggcctcggagagcggcgcaaactcggtcacggcctttgtcacgccgcgctacctctcgccctggagctcgcaggccaccagcatcgcccacgtgtgcgggctcaaggaccatgtgcgacgcgtcgagaggggccgcctcgtcgtcgtcgagttcgaggcgccgcttcacccggggcaagacgcctcgtttcgccacgtcctctacgaccgcatgactgagacctttgcgctcgagaaaccgtccctacatgccatgttcgaggcgggcgcgccgtcgcctctcgtcatcgtcgatatctttgccggtggctgcgattcgatcggcatcttgcgagagtataaccagcaaaagggcctcagtctcgacgagtccgagatgcagtacctcgtcgacgcctttacggagctgggccggccgccccacgatgtcgagctcttcatgtttgctcaggtcaactcggagcattgccgccacaaagtcttcaacgccagctggacaatcgacggagtggctcaggacaagagtctcttcgagatgatccgaaacacccacacaaagacgccagaatataccgtctctgcctacagcgataacgccgctgttcttcagggcgagctggccaacttctgggcccccgactactccaccgggacctggaagctcacccccgaggttgcccatattttggccaaagtagagacgcataatcatcctaccgccatctcaccgtttcccggtgccgctaccggttccggcggggagattcgggacgaagcggccgtgggcaggggctccgtcacaaagggaggcctctgcggattctgggtgtctgacctccttatccccgaccaccgtgctccttgggagctcgacatcggtcgcccggcacactacgccagcagtctcgacatcatgttggaggcccccatcgggagcgcgcgattcaacaacgagttcgggcgtccatgcctcactggctgcttccgccccctcctgactccggccgctggccatgggtcttccgcaagcgaggcaaaggagtggcgcggataccacaagcccatcatgctggccggcggcgtgggatccgtccgtccccaaaacgcactcaaggaggaaaagcttgtccgcgaaggcgcacacgtcatcgtcctcggcggcccagccatgctcatcggcctcggcggtggcgctgcctcgagcaacgcgtcgggtgaaggtaacgccgacctggactttgacagcgtgcagcgtggcaacccagagatggaacgacgggcccagatggtcatcaacacatgcacggcactcggagaccagaacccgattgccatgatccacgatgtcggtgccggaggtctttcgaatgctcttcccgaactcgtcaaggacgccggctacggaggcaactttgagctccgccaggtcgagagtgcagacagcagcatgagccccctccagatatggtgcaacgaggcgcaggaacgatacgttctcttgatcaactcggagaacatgaaccgcttcaccagcatctgccgtcgggagcgctgtggcttctccgacgtcggggctgttgcctcgaccgacgccggcggcgtggccaagttggtccttaccgaccgggagtccaaggaatatccgcgaccgattgatctgcccatggacactctcttccctcccaagcggcagcagaatcgcacagtggcgtctcgcaagccacaacttctaccctttgatgccctgggctccctccgacagacgtgtggtgacgacgtcgagactgctgacctgttcaagaaagccgtcgagcgtgtgttcctggtgccagccgtcggatccaagtcgttcctcatcacaatcggcgacaggaccgtcggcggcctcaccacgcgcgatcaaatggttgggccgtggcagacgcccgtggcagacgtggcggttacggcgacgggctttagcctgggcggcaaacaaagcaccggcgaggccatggcggtgggagagaagccgaccctagcgctgattgatgccgccgcttcggcaaggatggcggtggcggaaagtctgttgaacattggcgccgccgacatcatgggcgacttgcgacgcgtcaagctggctgcgaactggatggcggccgtcaaccaccctggcgaaggcgccgccctgtacgaggcggtccgtgccattggcatggagatgtgccccgagctgaacattagcatcccggtcggcaaggattccacttcgatgaaggccgtttggaaggacaaggaggagctgaagagcgtgacggcgcccgtctcggtcgccatctcggcgtttgccgttgtcgaggacgtgcggaagacgtggacgccacagctccggagagtcgacgaggtgggagagacgatcctcttgtttgtcgacctcgccgaggggcggcaggcgctgggcggctcggcgctggcgcagtcgtttgggtccattggcgatgaagctccggacatgaagaactttggtctcatggccgactactttgacgcgctggggcagctgcacaagagcggcgtggtgctggcgtaccatgaccggtccgacggcggtttgatgacgacgattgcggagatgatgtttgctggccggtgcggcgtcgacatgatgatggacggcatctccaagtctggtcgggcgtctgacatggtcgaggctctcttcaacgaggagctcggcgccgtcttccaggtgcgggccgaggacgagatcaacttcaagagatgctttgcgacgtgcgggccgccggctgggctggtccgcaagtttgccgtggtcaagtccaagggcaggcagacgctgacgattcgccacggcggcactacctttgccacgctggaccgcgccgagatgcagcagtggtggtccaagacgtcccacgagatgcagcggctacgggacaacccggcctgcgccgagtccgagtattccaccattgcggactcggaggatccgggcatctcgtacaggctcaccttttcgccgtcggacaagatcatgcccctgacgtcgtctatctcgggcttctttggcaagacgccgcgcgtggccatactccgggagcagggcgtcaatggccatgccgagttggcctttgccttcaaggcggcaggcttccagccagtggacattcacatgacagacatattggatggccgctccctggccgactttacgggcctggctgcccccggtggcttctcctacggcgacgttctcggtgccgggcagggctgggccaagtcgatcttgatgcacgagaatgcgcggcgggagtttgccgagtttttcaagcggacagacacgtttgcgctgggagtctgcaacgggtgccagctgctcacgaggatcaaggagctgattcccggcgcggagcactggccgacgtttgtcgacaatgcctcgcagcagtttgaggcgcggtacagcatggtcaagattgagcaagacgagagcaagccgtcggtctttttccacgggatgaacgggtcggccctccccgtggtggtctcgcacggagaaggcagagcggcgttcccgtcgcccaactcgctacaggagctgtcgggcgcgggcatgatcccgctgcgctacgtggacaaccgcctcaaggtgacggagcggtacccgctgaacccgaacggcagtccgggcggcgttgccggcgtgtcgaccaaggacggccggttcgtggccatgatgccccaccccgagcggacgattctggccgacgtgagcagctacgtgccgcaggaggcagtcgaggaatggggcgagtttgggccgtggttgcgcgtcttccgcagcgcacggaggtgggtggggtag

**Protein sequence**

MPHETLVGGSCYTAPTAQELKARANKTSQPKIKDIRGQWVYYVDLRRADATVVGQVKELLHDVDSQPLLASESGANSVTAFVTPRYLSPWSSQATSIAHVCGLKDHVRRVERGRLVVVEFEAPLHPGQDASFRHVLYDRMTETFALEKPSLHAMFEAGAPSPLVIVDIFAGGCDSIGILREYNQQKGLSLDESEMQYLVDAFTELGRPPHDVELFMFAQVNSEHCRHKVFNASWTIDGVAQDKSLFEMIRNTHTKTPEYTVSAYSDNAAVLQGELANFWAPDYSTGTWKLTPEVAHILAKVETHNHPTAISPFPGAATGSGGEIRDEAAVGRGSVTKGGLCGFWVSDLLIPDHRAPWELDIGRPAHYASSLDIMLEAPIGSARFNNEFGRPCLTGCFRPLLTPAAGHGSSASEAKEWRGYHKPIMLAGGVGSVRPQNALKEEKLVREGAHVIVLGGPAMLIGLGGGAASSNASGEGNADLDFDSVQRGNPEMERRAQMVINTCTALGDQNPIAMIHDVGAGGLSNALPELVKDAGYGGNFELRQVESADSSMSPLQIWCNEAQERYVLLINSENMNRFTSICRRERCGFSDVGAVASTDAGGVAKLVLTDRESKEYPRPIDLPMDTLFPPKRQQNRTVASRKPQLLPFDALGSLRQTCGDDVETADLFKKAVERVFLVPAVGSKSFLITIGDRTVGGLTTRDQMVGPWQTPVADVAVTATGFSLGGKQSTGEAMAVGEKPTLALIDAAASARMAVAESLLNIGAADIMGDLRRVKLAANWMAAVNHPGEGAALYEAVRAIGMEMCPELNISIPVGKDSTSMKAVWKDKEELKSVTAPVSVAISAFAVVEDVRKTWTPQLRRVDEVGETILLFVDLAEGRQALGGSALAQSFGSIGDEAPDMKNFGLMADYFDALGQLHKSGVVLAYHDRSDGGLMTTIAEMMFAGRCGVDMMMDGISKSGRASDMVEALFNEELGAVFQVRAEDEINFKRCFATCGPPAGLVRKFAVVKSKGRQTLTIRHGGTTFATLDRAEMQQWWSKTSHEMQRLRDNPACAESEYSTIADSEDPGISYRLTFSPSDKIMPLTSSISGFFGKTPRVAILREQGVNGHAELAFAFKAAGFQPVDIHMTDILDGRSLADFTGLAAPGGFSYGDVLGAGQGWAKSILMHENARREFAEFFKRTDTFALGVCNGCQLLTRIKELIPGAEHWPTFVDNASQQFEARYSMVKIEQDESKPSVFFHGMNGSALPVVVSHGEGRAAFPSPNSLQELSGAGMIPLRYVDNRLKVTERYPLNPNGSPGGVAGVSTKDGRFVAMMPHPERTILADVSSYVPQEAVEEWGEFGPWLRVFRSARRWVG

***Hirsutella sinensis* strain L0106 phosphoribosylformylglycinamidine cyclo-ligase (purM-1) mRNA, partial cds.**

**(GenBank accession number, KP090950)**

**Gene sequence**

ctgctcattaggtcagggccgcaggactactattctaacaacaatcccctgccgctgccgaccgaactgatcgacagactcgcagatacagcgaacatgaccgatctccgcgttctgctcattggcggtggcggccgcgaacatgcgcttgcatggaagctgagccagtcgccccgagtcaattgcattctggtagtgcccggcaatggtggcaccgcgtcatgcccgaagacggcaaacgtgacctccgttgcggccgatgatttttcagggctcgttgatttctcaaggtcaaatggcatcaatctggtcgtcccgggccccgaagcgctactcgtcaacggggtagagggcttcttcaagagggccggcataccgttcttcggcccgtccaaggaggcggcgcggctggaaggcagcaagacgtactcgaaagattttatgatgaaataccagattcccacggccgcatacgaaaacttttccgactacgacaaggccatgcgctacctcgacagggttagtcacgacatcgtcatcaaggcgacaggcttagcggccgggaaaggcgtcattctcccccagacgaaagaagaggccaaggatgctctgaagcagatcatggtggacaaggctttcggtgctgctggcgacgaggtcgtcatcgaggagcttttgctcggggaagagctcagtgtcctcacattttgcgacggatatacaaccaagtccctaccgctcgctcaagaccacaagaggatattcgatggagatcagggccccaacacaggcggcatgggttgctatgcgcctactaacattgccacgcgggagctgatggaaataatcgacgcggaaatagtcaagcccaccatatcaggcatgcgtcgagagaggcatcccttccgaggcatcctgtttaccggcctcatggtcacgagcgcgggacccaaggtcctcgagtacaatgtgcgattcggtgaccccgaaactcagaccgttctgccactgctgtcaggcgacaccgatctggccgacatcatgctcgcctgcgccgagggatacttagacgactgccatctcaagatcgacaacaagttcagcgccaccgttgtcctggccgcccgaggttaccctggctcatacgccaaaggcacttcgatgaccgttcgggatgcttctaaaggaagcacaatcttccacgccggcaccaatctgagcgaaggccagctgacgacatctgggggccgtgtcattacaatcaactcggtcggcgattcgctgagggacgctgtcgatgcgtgttacgccgccctctcctctggagtaattgagttcgaggggatgttcttccgaaaggacattgcacatcgggccctcagagaatcgagcagggtcgccctgacctacgcccaggctggcgtggacatccaagcaggaaacgacttcgtggaaaaaatcaagcaggcggttgccagcaccaagcgacctggcgctgacgccgaaattggcggtttcggtggagagttggacctctcccagtgtggataccaagacgcgcccatcttggtcgccgctatcgacggcgtggggacgaaactcatgattgcgcagtcgatgaagcggcacgataccgtgggcattgatctagtggccatgaatgtcaacgacttgattgtccagggggccacgccgctcatgttcctcgactactacggctgcagtaaactcgacttggcctcggcttcctcgtttgttcggggcgtcgccgatggctgccgccaggccggatgcgccctggttggcggggaaacggcagagatgccgggcatgtatcagggcgaggactacgacgcagccggttgcgcgattggcgccgtcgtcgcctctagccgcctcccacgcaaagacaccatggttgaaggggacgtgctcttgggcttggcatctcacggcgtccactccaacggcttctccctcgtgagacgcattgtgcagagagccggtctcgactacaactccaaggcgccctgggactcatgccggactgtcggcgaatcgctgctaacgcccacccgcatctacgttacgagccttctccctgtccttgcccagataaagggcctggcacacatcaccggaggcggcctcatcgagaacgtgccgcgcatgcttcccgaggctcgcctcgctgccgacatcgactttggctcgtgg

**Protein sequence**

LLIRSGPQDYYSNNNPLPLPTELIDRLADTANMTDLRVLLIGGGGREHALAWKLSQSPRVNCILVVPGNGGTASCPKTANVTSVAADDFSGLVDFSRSNGINLVVPGPEALLVNGVEGFFKRAGIPFFGPSKEAARLEGSKTYSKDFMMKYQIPTAAYENFSDYDKAMRYLDRVSHDIVIKATGLAAGKGVILPQTKEEAKDALKQIMVDKAFGAAGDEVVIEELLLGEELSVLTFCDGYTTKSLPLAQDHKRIFDGDQGPNTGGMGCYAPTNIATRELMEIIDAEIVKPTISGMRRERHPFRGILFTGLMVTSAGPKVLEYNVRFGDPETQTVLPLLSGDTDLADIMLACAEGYLDDCHLKIDNKFSATVVLAARGYPGSYAKGTSMTVRDASKGSTIFHAGTNLSEGQLTTSGGRVITINSVGDSLRDAVDACYAALSSGVIEFEGMFFRKDIAHRALRESSRVALTYAQAGVDIQAGNDFVEKIKQAVASTKRPGADAEIGGFGGELDLSQCGYQDAPILVAAIDGVGTKLMIAQSMKRHDTVGIDLVAMNVNDLIVQGATPLMFLDYYGCSKLDLASASSFVRGVADGCRQAGCALVGGETAEMPGMYQGEDYDAAGCAIGAVVASSRLPRKDTMVEGDVLLGLASHGVHSNGFSLVRRIVQRAGLDYNSKAPWDSCRTVGESLLTPTRIYVTSLLPVLAQIKGLAHITGGGLIENVPRMLPEARLAADIDFGSW

***Hirsutella sinensis* strain L0106 phosphoribosylaminoimidazole carboxylase (PAICS-1) mRNA, partial cds.**

**(GenBank accession number, KP090953)**

**Gene sequence**

atgtctcgcccccccgtgattggactgctcggcggcggccagctgggccggatgctgtgcgaggcggccggaccgctgggctaccacgtcgtcgtgctcgacgaggacggatgcccggccaagcaggtcaacggcagcgacaggcacgtgtccgggtccttcaaggacgccgccaaggtgcgggagctagcggcacgctgcgacgtgctgacggtcgagattgagcacgtcaacgccgatgtgctccaggaaattgccgtccgcggcgtgccctcggcctcgggcgagctgaggagagtgcctgtccacccgtcgtggcgcacactgcgccttgtccaggataagtacgagcaaaaggagcactttcgcgcaaacggcatccccgtggcgccgcagatggccctcgacggcggcgagttcctggccgcctcgctcaaagacgcctgccaccacctggcctctcccttcatggtcaaggcccgcaagggttcctacgacggccgcggcaacttcaaggtcggcgggctcgacgacttcgaggccgccatagacgccctcggcgccctgccgctctacgccgagaaatgggtcccctttgccatggagctggccgtcatggtccttcgcaccgaggacgacgccggcgagtgcacgggcgtctactcgtacccggccgtcgagacggtccacgaggacgacgtctgcaagacggtcctgatgcccccgcgcagcgtcgacgctgccgtctgccacaaggcccagcgcgttgctgagcaggttgtcaggagcttgtgggggaggggcgtctttgccgtcgagatgttcctgctgcaggacggaaccatcatggtcaacgaggtggcgcccaggccgcacaactcgggccactacaccatcgaggcggtgccgtacatgtcgcagtacaaggcacagctctgcgccatcctcgaccttgtgcccaagtcgcttcgcctgacgccgcgcgccgcccaggccgccatggtcaacatcctgggcggcgccgccccggactcgcacgacaagctcgtcggcctggccgagactgagtacattgacggcaccgacatttatctgcacctctatggcaaggcgtccaagcccggccgcaagattggccacattacctttaccacgccgtcgcccgacgtcgacctgctccgcaccattggcccctttatcaaccaggtcgacgccatgaggcgggagcgccttgacgcctcggccaagcagctgcgccccgaggcagcgggcgcctcgtcttcggacgaactcacccagaccgccaagggcggcagcggcaagagctcgcgcgacgccaagtcgcccctcgtcgtggtgaccatgggctctgactcggacctgtcggtgctgtcggccggcctcgacgtcctggagcgcttcggcgtgccctacgactgcaccatcacgtcggcgcaccgcaccccggcccgcatggccgagctgggacgcgacgccgcggcgcgcggcgtcaaggtgctcatcgccgcggccggcggcgccgcccacctgccgggcatgctggcctcggagacgacggtgcccgtcattggcgtccccgtcaaggcgacccatctcgacggcaacgacagcctactgagcattgtgcaaatgcccaggggcatccccgtggccacggtcggcatcaacaacccgacaaacgccgccctgctggcgatccgc

**Protein sequence**

MSRPPVIGLLGGGQLGRMLCEAAGPLGYHVVVLDEDGCPAKQVNGSDRHVSGSFKDAAKVRELAARCDVLTVEIEHVNADVLQEIAVRGVPSASGELRRVPVHPSWRTLRLVQDKYEQKEHFRANGIPVAPQMALDGGEFLAASLKDACHHLASPFMVKARKGSYDGRGNFKVGGLDDFEAAIDALGALPLYAEKWVPFAMELAVMVLRTEDDAGECTGVYSYPAVETVHEDDVCKTVLMPPRSVDAAVCHKAQRVAEQVVRSLWGRGVFAVEMFLLQDGTIMVNEVAPRPHNSGHYTIEAVPYMSQYKAQLCAILDLVPKSLRLTPRAAQAAMVNILGGAAPDSHDKLVGLAETEYIDGTDIYLHLYGKASKPGRKIGHITFTTPSPDVDLLRTIGPFINQVDAMRRERLDASAKQLRPEAAGASSSDELTQTAKGGSGKSSRDAKSPLVVVTMGSDSDLSVLSAGLDVLERFGVPYDCTITSAHRTPARMAELGRDAAARGVKVLIAAAGGAAHLPGMLASETTVPVIGVPVKATHLDGNDSLLSIVQMPRGIPVATVGINNPTNAALLAIR

***Hirsutella sinensis* strain L0106 phosphoribosylaminoimidazole-succinocarboxamide synthase (purC-1) mRNA, complete cds.**

**(GenBank accession number, KP090954)**

**Gene sequence**

atggctctcacaaccatcaaccttccctcgctggaaaaggtggcctcgggcaaggtacgcgacctcttcgcccttgacgaccccaatgcgctgctctttgtcgcgtcggaccgcctgtcggcctttgacgtcgtgatgaagaacggcatccccaacaagggcgccatcctgaccctcgcctctgcccactggttccgcgttctatcagagcgcattcccggcctgaggacgcactttgtcacgctcgacgtgcccgcaagcctgtccgtcgaggaggccaagaccattaagaaccgcagcatgcaggtgcgcaagctccaggtgctcaagatcgaggccattgtgcgcggctacttaaccggcagcgcctggaacgagtaccaggccaagggtacggtccacggcctagccatgccggcggggatgcagcagtcgcaaaagtttcccgagcccatctacacgccaagcacaaaggccgatctcggcgagcacgatgagaacatacatcccgacgatgcctggaaggagctgggcgacaaggagacggctcgccaggtccaagagctgtccctcaaaatctacaaggccgcagcggaatacgccgaggagcgcggcatcatcatcgccgacaccaagtttgagtttgccaaggacgacgagggccacatttacctcgtagacgaagtgctgacgccggattcgtcgcgtttctggccaaaggacacatacgacgtgggcagagaccaggacagcttcgacaagcaattcgtacgcaactggctcatcaaggagggtctcaagggcaaggagggcgtcgagattcccgaagacatttgcaaggccaccgagcagcggtacaaggatgtatttctgaagctcatgggaaagacgtttgatgacgcggtccgcagtaaagtccacgaagatacaaacgtcaccagataa

**Protein sequence**

MALTTINLPSLEKVASGKVRDLFALDDPNALLFVASDRLSAFDVVMKNGIPNKGAILTLASAHWFRVLSERIPGLRTHFVTLDVPASLSVEEAKTIKNRSMQVRKLQVLKIEAIVRGYLTGSAWNEYQAKGTVHGLAMPAGMQQSQKFPEPIYTPSTKADLGEHDENIHPDDAWKELGDKETARQVQELSLKIYKAAAEYAEERGIIIADTKFEFAKDDEGHIYLVDEVLTPDSSRFWPKDTYDVGRDQDSFDKQFVRNWLIKEGLKGKEGVEIPEDICKATEQRYKDVFLKLMGKTFDDAVRSKVHEDTNVTR

***Hirsutella sinensis* strain L0106 adenylosuccinate lyase (purB-1) mRNA, partial cds.**

**(GenBank accession number, KP090955)**

**Gene sequence**

ctgcgtgacgggctagacttgcttcttcccaagctcgccagggtcatcagctccctttccaagtttgcctgccagtataaggacctgccgacgctgggcttcacgcactatcaagcggcgcagcccattacccttgggcgccgcgccgcccagtggctccaggacctcgtcttcgatctcgacgatgtcgaatacgttagggccggactgcggttccgcggcgctcagggcaccaccggcacgcaggcgtcgtttatggagattttccaaaacgacgccgccaaagtcgacaagctgaacgagatgctctgccagaaggcgggcttcaccggctgctacgacatctccacccagacctacacccgcaaggtcgatctgcgagtagccaatgctctgtccgccctaggtgccacagcgacacgcattgcaacggatatccgccatctctgccatgacaagctgctggacgagcctcatgtcgctggccagattggctcg

**Protein sequence**

LRDGLDLLLPKLARVISSLSKFACQYKDLPTLGFTHYQAAQPITLGRRAAQWLQDLVFDLDDVEYVRAGLRFRGAQGTTGTQASFMEIFQNDAAKVDKLNEMLCQKAGFTGCYDISTQTYTRKVDLRVANALSALGATATRIATDIRHLCHDKLLDEPHVAGQIGS

***Hirsutella sinensis* strain L0106 phosphoribosylaminoimidazolecarboxamide formyltransferase (purH-1) mRNA, partial cds.**

**(GenBank accession number, KP090956)**

**Gene sequence**

ctgggtgtacttgagggtgattgtggcgacagtgaggtcgcgagcggcggcctcggacagggcccccgcgtccttgggggtgatgattgtgccaaacgaccttggagagatttcgacgtcgttgcggtgctgctgcaggttgattccgtagacggtgcgcgtctcggtgggcgcgggggggtactcggggtccatctggaggacgaggtacttgccgccctttttcttccggagcatctcgagtgcggcatcctcgtagccgggggcgatgacgccgtccgatacctccttggaaatgatggtggcggtgggcacgtcgacgacatcgctcagggcgatcatgtcgccaaagctgctcatgcggtcggcgccgcgggcgcgggcgtaggcctgggccagtggggaggagtcgatgccgtcaatgtcgccgacaaagtagacttggcgctcctcgggggtcaggggcaggccaatggcggcgccggcgggggagacgtgcttgaagctggcggcggcgggcttgcccagggcctgcttgagctccttgacgagcggccaggcattcaggcagtcgaggaggttgatgtagccgggggaaccacagaggaccttgaagggcagcttgtcaaagcttgtaaaggcggtggcgggcttctgatggggatttgcgccgtagcggagggccatgtactggtcgtcggcgtactccttgcggaaaaagtcggagatggcggcgtcgtagtcggccgtgtgctcaaaggccttgagtgcgtaccggttgcggctcgtctctgtgatttcgccctgctcgagctccttgaggaagccggcataatcgttggggtcgctgagaatggtgacgcgcttgtggttcttggccgcggcgcggatgagggtaacgccgccaatgtcaatctcctcgacggcctcggggatgctgacgtttatctttgcgacggtgtccttgaagggatacaggttgcagatgacgtagtcgaccttgttgatgttctggtcggccaggtccttctcatccgactcgaggttgcgggcgaggatgccggcgtggacggcggggtggagcgtcttgacacggccggcaagcatctcaggggccttcgtgatggcgcttatgtcctcgacggggaagcccgagtcgcgaatcatgcgagcagtgccccccgaggccaggatgcggacattctgctgcaccaagcccttggccaggtccagcaggccagtcttgtcgtagacggagacgatggcaatcttttggtctgctggcatggcgggcgaatttgttctcgtcactctgcgagggtctgcgaggagcccttcgtgttatatagtattgggggaaacgaggaagaagcaattgcgagaagcagagagaaa

**Protein sequence**

LGVLEGDCGDSEVASGGLGQGPRVLGGDDCAKRPWRDFDVVAVLLQVDSVDGARLGGRGGVLGVHLEDEVLAALFLPEHLECGILVAGGDDAVRYLLGNDGGGGHVDDIAQGDHVAKAAHAVGAAGAGVGLGQWGGVDAVNVADKVDLALLGGQGQANGGAGGGDVLEAGGGGLAQGLLELLDERPGIQAVEEVDVAGGTTEDLEGQLVKACKGGGGLLMGICAVAEGHVLVVGVLLAEKVGDGGVVVGRVLKGLECVPVAARLCDFALLELLEEAGIIVGVAENGDALVVLGRGADEGNAANVNLLDGLGDADVYLCDGVLEGIQVADDVVDLVDVLVGQVLLIRLEVAGEDAGVDGGVERLDTAGKHLRGLRDGAYVLDGEARVANHASSAPRGQDADILLHQALGQVQQASLVVDGDDGNLLVCWHGGRICSRHSARVCEEPFVLYSIGGNEEEAIARSREK

***Hirsutella sinensis* strain L0106 adenylosuccinate synthase (purA-2) mRNA, partial cds.**

**(GenBank accession number, KP090957)**

**Gene sequence**

atggggtcagttcagttcgcgccgcccagcgccgtccaacaacgccgaccccctggtccgtacccgagacttgacactgacgccgccgcaggagacgagggcaagggcaaactcaccgacatcctctgccccgacttcaagctctgcgcccgtgctgccggcggccacaacgccggccactccatcgtcgccaacggcgtctcttacagctttcacctcctcccctccggcctcgtcaatcccaactgcctcaacctcatcggctccggcgtcgtcttccacgtgcccagcttcttcaaggagcgcgccgagctgcaagaaaagggcctcgacaccaccgaccgcatcctcgtctccgataga

**Protein sequence**

MGSVQFAPPSAVQQRRPPGPYPRLDTDAAAGDEGKGKLTDILCPDFKLCARAAGGHNAGHSIVANGVSYSFHLLPSGLVNPNCLNLIGSGVVFHVPSFFKERAELQEKGLDTTDRILVSDR

***Hirsutella sinensis* strain L0106 5'-nucleotidase (5'-Nuc-1) mRNA, partial cds.**

**(GenBank accession number, KP090958)**

**Gene sequence**

ttggaaacagctcccctcggcccagtagttctgcagcacgtttgtccacagcgtcttgtgcttttccacgtcctcgaccagcgggacgttgacgctgtacagatcggcgctggcgtcagtgggccactgcttgtacagggcgtcgatgacctttacgctgtggcggcacgcggcctcgatgatgacgggatcgtggttgcgcgagaaaaaggcaaagctcagagcaatggaccgcctcctgcagacggcggcctcgagcgcggcgcccagggtgcccgagctcagagcaaagacggagctcgtgttcctgccatagttgggtccgctgacgaccaggtcgatggggcccttgtcccggaagaagtggtgcagaccaatctgcacgcacgacgcgggcgtgccgtcaatcaggatccattcctcgacgtcgcccgccgtcgacggccggtggtgtgtcgtgccctcggagtcgtctccgtggacctttgcactcgggcgatagtagagcggctttcg

**Protein sequence**

LETAPLGPVVLQHVCPQRLVLFHVLDQRDVDAVQIGAGVSGPLLVQGVDDLYAVAARGLDDDGIVVAREKGKAQSNGPPPADGGLERGAQGARAQSKDGARVPAIVGSADDQVDGALVPEEVVQTNLHARRGRAVNQDPFLDVARRRRPVVCRALGVVSVDLCTRAIVERLS

***Hirsutella sinensis* strain L0106 5'-nucleotidase (5'-Nuc-2) mRNA, complete cds.**

**(GenBank accession number, KP090959)**

**Gene sequence**

atgggcaacgacacgacggcgccctcgtcggagccgaggcggaccttttcgtcgggcagagatgaagccgcggatgccccggacctgcgcgtcctccactacaacgacgtctatcatgtcgacccggccagtgccgagcccgtcggaggccttgcgcgcttcatgaccttggtcaccgagtaccgggcgggccagcagtacagccagctgcccagcctcgtgaccctcttctccggcgacgccttcaacccgagtctcgagagcagcgtcaccaagggcaggcacatggtgcccgtcctcaacaccatcgggaccgattgcgcttgcgtcgggaaccacgacttggactttggcgtcgatcagttccgccacctcgcccaaaagtgcaagttcccatggctcctggccaacatcctcgacccggccctgggcgacgacgtgcctctgggccacgccgaccgcacccacatgtacaccgcgtcaaacggcatcaaggtgggcctcattggcttgggcgaaagagagtggctggagacgatcaacagcgtcccgcccaatctcatctatcgttcagcgactgcaacggcaaaggaactggtccccaagctgcgcgagcagggcgccgacattgtgatatgcctctcacacatgcgcgagcccaacgacaacaagctcgcccaacagacggacggcatcatggacatgatcctaggcgggcatgaccactactacagccacagcttggtcaacggcacccacgtcctgcgctcgggcaccgacttcaagcagatgagctacctcgaggcgcgtaggaaaagggacggctccggaaagtgggactttgacatctggcgccgcgacgtcacctcggacgtgccagaacacgagccgtcggccgagctcgcccgccatcttacctcgaagttgcaaaagtctctgtcgcggcccattggctggacggccatgccgctcgacgctcgcttcagcaccgtgcgcgtcaaggaatccaacattggcaactttgtctgcgacgtgatgcgacgctaccacagcgccgactgcacaatcatggctggcggcaccatccgtggcgatcaaatctatcctcccggagctgttcgaatcaaggacataacaaattgcttcccttttgaggatccagtcattctgctgcgagtctcgggccaagccatttgggatgctttggagaatggtgtttcgttgtatcccgctctagagggcaggttctctcacgtctccaacatggcctacgagttcgacccgagcagggagagtggcaagagactcacctccttgtgcatcgggggcgaggaatggatcccggagaagcagtatcttctcgcgacgagaggatacatggggcgcggaaaagacggcttcaccagcttgctcgtgaaatcagagggcggccaggcggaggaacttgtcgatgaagagcacggcattctgatttcagccatgttgcgacagtactttatgggcctgcagaccattggccagtggaagaagctgtcggaccactgggtcggcgtggccaagggcgccgtgccactggcatcggtgcgaaagagggagcacagcaaaatggatgacagtccgggtgtcttggggtcgctcacctcgagcggtaagcagcacggggacggcgagacaacttggcatggcttcctacaccgacgccttggcctcggcgagaagcctttggacgacgacgacgacgacgatcacttcgaccatgtacaccaagacgagggcccagacaacggcgacaccacggacaagatggattgcgaggtgctgcttttgagaaagttttggaaccgctgggcacgcaaggcgggcgtcaaggcaggcgtgtgcgatggcatgaaggagggcgagtttgacgtggattggacgcgagtgattgctccacgcctcgaggggagaatcaagatgcgggcaacgtga

**Protein sequence**

MGNDTTAPSSEPRRTFSSGRDEAADAPDLRVLHYNDVYHVDPASAEPVGGLARFMTLVTEYRAGQQYSQLPSLVTLFSGDAFNPSLESSVTKGRHMVPVLNTIGTDCACVGNHDLDFGVDQFRHLAQKCKFPWLLANILDPALGDDVPLGHADRTHMYTASNGIKVGLIGLGEREWLETINSVPPNLIYRSATATAKELVPKLREQGADIVICLSHMREPNDNKLAQQTDGIMDMILGGHDHYYSHSLVNGTHVLRSGTDFKQMSYLEARRKRDGSGKWDFDIWRRDVTSDVPEHEPSAELARHLTSKLQKSLSRPIGWTAMPLDARFSTVRVKESNIGNFVCDVMRRYHSADCTIMAGGTIRGDQIYPPGAVRIKDITNCFPFEDPVILLRVSGQAIWDALENGVSLYPALEGRFSHVSNMAYEFDPSRESGKRLTSLCIGGEEWIPEKQYLLATRGYMGRGKDGFTSLLVKSEGGQAEELVDEEHGILISAMLRQYFMGLQTIGQWKKLSDHWVGVAKGAVPLASVRKREHSKMDDSPGVLGSLTSSGKQHGDGETTWHGFLHRRLGLGEKPLDDDDDDDHFDHVHQDEGPDNGDTTDKMDCEVLLLRKFWNRWARKAGVKAGVCDGMKEGEFDVDWTRVIAPRLEGRIKMRAT

***Hirsutella sinensis* strain L0106 N-glycosylase/DNA lyase (N-Gly-1) mRNA, complete cds.**

**(GenBank accession number, KP090960)**

**Gene sequence**

atggggcttggttggggcgagtctgtgccagtcgacactcatgtttggcaaattgcccagagggactacaagtttggcaggtccaagacaaagactttcaacaaggtcctgtacgatgcggtcggggatcacttccgcgacgtttggggcaagtatgcgggctgggcgcactcggtactttttgcagcccatcttcgagaattttcggatcgcagtgtgaagaaggaggacgccgagcctttgcaaacgcacatgaacgaagggcaagcggtggacgggacccgcaagaggatgaccgcggcgaaggaggtcaagacggaagtcaaagtggaagagactgagcatgggaccgttgcggaagtagagagggccatgaagaggagacggacgacgcggcagaggccctga

**Protein sequence**

MGLGWGESVPVDTHVWQIAQRDYKFGRSKTKTFNKVLYDAVGDHFRDVWGKYAGWAHSVLFAAHLREFSDRSVKKEDAEPLQTHMNEGQAVDGTRKRMTAAKEVKTEVKVEETEHGTVAEVERAMKRRRTTRQRP

**Purine nucleotides anabolic functional genes from *Hirsutella sinensis* strain L0106 (Anamorph *Ophiocordyceps sinensis*).**

***Hirsutella sinensis* strain L0106 purine nucleosidase (iunH-1) mRNA, complete cds.**

**(GenBank accession number, KP090961)**

**Gene sequence**

atgaccatgccagactcgtcgggccagattcccgtctggctggactgcgatccgggccacgatgacgtgtttgccatgctgctggcggcacaccacccccggatcaagctactaggcatctcgaccgtgtttggaaatgcctcgctcgaacacacgacgcgcaacgccgcctcggtcctcacggcgctcggcaagcacgccgacgtacctctgcacgccggtctggccaaggcgctcgagcggccggccctgcacgcgcccaccaacatccacggcgactcgggcctcgacggcaccgacctgctgccggagcccgcgtgccggccctcggacgtgccggccgtcgatgccatggccgccgccctagccgcccagccgtcgggcaccgcctgggtcgttgccacgggcgccctcacaaacgtcggcgccctattccgcgcccgcccagacctcgtcgcccacgtccgcggcctcagcctgatgggcggctccctcggaggcggcttctccgacgcgccaatgggccacgtcgaccaccatcggaaacgccaccccctgggcagagttcaacatattcgtcgacccagaggccgccgcccacgtcttccacagccccgacattgccgccaagacaaccgtcgtgcccctcgacctgagccaccaggtcctcgccaccgaccaggtccgcgacctgctcctctacggcgcagacggcgacagggccggcccgggcaagtcgacactgcgcaccatgctcgtcgagctcctctactttttcgcaaagacgtattcgtaagttgcattttctcggcccgcctcagccagggctttatgctctctaacggcacgcgttag

**Protein sequence**

MTMPDSSGQIPVWLDCDPGHDDVFAMLLAAHHPRIKLLGISTVFGNASLEHTTRNAASVLTALGKHADVPLHAGLAKALERPALHAPTNIHGDSGLDGTDLLPEPACRPSDVPAVDAMAAALAAQPSGTAWVVATGALTNVGALFRARPDLVAHVRGLSLMGGSLGGGFSDAPMGHVDHHRKRHPLGRVQHIRRPRGRRPRLPQPRHCRQDNRRAPRPEPPGPRHRPGPRPAPLRRRRRQGRPGQVDTAHHARRAPLLFRKDVFVSCIFSARLSQGFMLSNGTR

***Hirsutella sinensis* strain L0106 adenosine kinase (ADK-1) mRNA, partial cds.**

**(GenBank accession number, KP090962)**

**Gene sequence**

ctgtctcactcgcggttaggtaacattgcgtcggagattgacttgtcgtggtccattgggctcacgagtcttgggatggacggccccttgttgcaacaggagcgcagacagctttggccgtgccacccttcatttcctcagttgatggctgtgattcgcgatagcccccagagcgcgcccaacagagacgtctgtctgctcgtcgcgtgccaccaggccgacctcacatccgacaactacgaagccttgccccgcgcgcggcactgtcttttcgtcacgacaaggcgcagcaagagcttgaaaaacatcgcgtgcgccgaatgtcc

**Protein sequence**

LSHSRLGNIASEIDLSWSIGLTSLGMDGPLLQQERRQLWPCHPSFPQLMAVIRDSPQSAPNRDVCLLVACHQADLTSDNYEALPRARHCLFVTTRRSKSLKNIACAEC

***Hirsutella sinensis* strain L0106 adenine phosphoribosyltransferase (APRT-1) mRNA, partial cds.**

**(GenBank accession number, KP090963)**

**Gene sequence**

ctgccggcggccgttgagaccggggacttcgaggataaagaggtagccaacgacttggcccttgagctgggagacgagatcggccgcagccttggccgagccacccgtggcaatgatgtcgtcgacaatgagcaccttttggcccgggccgacggcgtcctgctgcatctggaagaggtctttgccgtactccttgacgtattcggccgtgacgcagggcccggggagcttgccctttttgcgcaccgtggcgaagccaacaccgaggcgcaaggcgaggccgggcccgaagaggaagccgcgggcatcgagaccgacaatgacgtctggctttacgtgggcaaacgactggtcaatctgccactcgaggg

**Protein sequence**

LPAAVETGDFEDKEVANDLALELGDEIGRSLGRATRGNDVVDNEHLLARADGVLLHLEEVFAVLLDVFGRDAGPGELALFAHRGEANTEAQGEAGPEEEAAGIETDNDVWLYVGKRLVNLPLE

***Hirsutella sinensis* strain L0106 AMP deaminase (AMPD-1) mRNA, partial cds.**

**(GenBank accession number, KP090964)**

**Gene sequence**

atggtgaagacgaacgtgccggacagaagggaagagtttcgataccataccctcttgcaggagagagacgttctcaaacgctacgtgacgtacgaggcaaagaacgagtcactcggcgaagggcttcccgccgatgggaacgcggcggagccggcaagggccgctttcaatctgggggaaccggcgctgacaactcaagagaggggcggagctgtgatcaagacgacctgtccggcagcagatggcgcgcaaccgagggccctgcgccaaacgggggggttgccaggtgccatggcagatttgcacttgtcgggaagcgacccgagaatgtttccaggaattctcacccgagatcacaggagtggcagggtacggaac

**Protein sequence**

MVKTNVPDRREEFRYHTLLQERDVLKRYVTYEAKNESLGEGLPADGNAAEPARAAFNLGEPALTTQERGGAVIKTTCPAADGAQPRALRQTGGLPGAMADLHLSGSDPRMFPGILTRDHRSGRVRN

***Hirsutella sinensis* strain L0106 AMP deaminase (AMPD-6) mRNA, partial cds.**

**(GenBank accession number, KP090965)**

**Gene sequence**

atgattggaatcacccagccccgacgagttgccgccgtgagcatgtcgaggagggtggccgacgagttgagcggccacccgggcgcggtagcatatcagatccgcttcgagggcaccgtgaacgccagaaccgccctcaagttcatgacggatggtgttttgctgcgagaggttgcgcaggatatcagtctgcgtaagtactcggccatcataatagacgaggcgcacgaaaggagcgtcaacaccgacattctcattggcatgcttagccgcgtcgtcaagctgcggaccgagcttgcccatgaggacccgacactcaagccgctcaagctcatcatcatgtctgcgacgctaaggatagaagacttgacgatgaactcgacg

**Protein sequence**

MIGITQPRRVAAVSMSRRVADELSGHPGAVAYQIRFEGTVNARTALKFMTDGVLLREVAQDISLRKYSAIIIDEAHERSVNTDILIGMLSRVVKLRTELAHEDPTLKPLKLIIMSATLRIEDLTMNST

***Hirsutella sinensis* strain L0106 IMP dehydrogenase (guaB-1) mRNA, partial cds.**

**(GenBank accession number, KP090966)**

**Gene sequence**

ctgcatccgcaatggccgccgccacggtccgctcgagcgccaagctgcttgaccacagcaaggcgctcgagatgctggccgaatacgaaacggctgatgggctcgacatccacgagctcatggacacggccaagcacgggggcctcacctacaatgacttcctgctcatgccgggctacatcggcttccccgcctccgaggtggccctcgactcccccatcaccaagcgaatcaccctcaagacgccctttgtctcgtcgcccatggacacggtcacggagcatgagatggccatccacatggctctgcagggaggcctcggcgtcatccaccacaactgctctcccgaggcccaggccggcatggtccgcatggtcaagcgttacgaaaacggcttcatcctcgac

**Protein sequence**

LHPQWPPPRSARAPSCLTTARRSRCWPNTKRLMGSTSTSSWTRPSTGASPTMTSCSCRATSASPPPRWPSTPPSPSESPSRRPLSRRPWTRSRSMRWPSTWLCREASASSTTTALPRPRPAWSAWSSVTKTASSS

***Hirsutella sinensis* strain L0106 IMP dehydrogenase (guaB-2) mRNA, complete cds.**

**(GenBank accession number, KP090967)**

**Gene sequence**

atggtgaccgacctcaccactgctcccgacggcgtcactcttctcgaggccaacaagatccttgccaagtccaagaaaggcaagcttcccatcgtcgacaaggacaacaacctcgtctccatgatctcccggtccgacttgaccaagaatcaacactttcccctggcgtccaagctacccgacagcaagcagctcatttgcgccgctgccatcgggacccggcccgaagacaaggaccgactcaagaagctcgtcgatgccggtctcgacattgtcatcctcgacagctcgcagggcaacagcatctaccagaccgacatgatcaagtggatcaaggtcgagttccccaagatcgacgtcatcggcggcaatgtcgtgacgcgtgagcaggccgcgtctctcatcgcagccggtgtcgatggcctccggattggcatggggagtggctctgcctgcatcacccaggaggtcatggccgtcggccgtccccaggccgccgccgtctattccgtcagccactttgctgcgcggttcggtgttccctgcatcgccgatggcggcgtgcaaaacgtgggccacatcgtcaagggtctttccttgggcgcctctaccgtcatgatgggcggtctcctggccggcaccaccgagtcccccggcacgtcgtttgtctcgcgcgagggcaagctggtcaaagcataccgcggcatgggcagcatcgacgcgatgcaggacaagaaggctggcaacggcggcaaggacagccagaagagcaacgccggcaccgcccggtacttttccgagggcgacagcgtcctggtggcccagggggtgtcgggagccgtggcgcaccggggctccgtcggcaagtttgtgccgtacctggctgccggcctcaagcactcgatgcaggattgcggcatgaagagcctggcggagctgcatgaacgcaccgccgacggcaccgtgcgctttgagcttcgcacctcgagcgctcagctcgagggcaacgtcaacatggaggcgtatgagaagaagctgtatgcatga

**Protein sequence**

MVTDLTTAPDGVTLLEANKILAKSKKGKLPIVDKDNNLVSMISRSDLTKNQHFPLASKLPDSKQLICAAAIGTRPEDKDRLKKLVDAGLDIVILDSSQGNSIYQTDMIKWIKVEFPKIDVIGGNVVTREQAASLIAAGVDGLRIGMGSGSACITQEVMAVGRPQAAAVYSVSHFAARFGVPCIADGGVQNVGHIVKGLSLGASTVMMGGLLAGTTESPGTSFVSREGKLVKAYRGMGSIDAMQDKKAGNGGKDSQKSNAGTARYFSEGDSVLVAQGVSGAVAHRGSVGKFVPYLAAGLKHSMQDCGMKSLAELHERTADGTVRFELRTSSAQLEGNVNMEAYEKKLYA

***Hirsutella sinensis* strain L0106 GMP synthase (guaA-1) mRNA, partial cds.**

**(GenBank accession number, KP090968)**

**Gene sequence**

ctgatctcccatcactccgacggccctactggtgtcgaggcctgcgtaggcttgagacatttggttgtagatgcccgcctccttgatcatggagatgaagatgtggtcggcctttctggcgatggcgacacgctcgggagtcacctctccaatgattctgatggcaatgcccggccctgggaacggatgacgcatgacaaggtcgtcgtggataccaagttgccggccaaattcccgcacctcgtccttgaacagctctctcaagggctcaatcagtttcaggccctcaccgttcatcatgcgagccggcagcccgcctacgttgtgatgt

**Protein sequence**

LISHHSDGPTGVEACVGLRHLVVDARLLDHGDEDVVGLSGDGDTLGSHLSNDSDGNARPWERMTHDKVVVDTKLPAKFPHLVLEQLSQGLNQFQALTVHHASRQPAYVVM

***Hirsutella sinensis* strain L0106 GMP synthase (guaA-2) mRNA, partial cds.**

**(GenBank accession number, KP090969)**

**Gene sequence**

ctgctccagaactttgccgtcgcgatttgcggagccaagcagcattgggtcatgagcgacttcatcaagcatgaaatcactcgcatccggaagctcgttggcgacaaagctcaagttatcggagccgtctcgggcggtgtcgacagcactgtggcggctcggctgatgcaagaggcgattggcgagagattccatgctgttcttgtagacaatggcgtgatgcgtctcgacgaatgccagcaggtgcaggagacgctgcaaaaacatctcggcatcaacctgacaatcgtcgacgggtccaagctgttccttgaccgcctaaaaggtgttactgaacccgagaagaagcgatccatcattgggggaacctttatcgacctcttcgaagaggaggccatcagaattgaaaaggtggccgagaataccccccatgctggcaaggtggagtggttccttcagggtactctgtatccggatgtcatcgagtctctgagcttcaagggcccttcagcgaccattaaaacacatcacaacgtaggcgggctgccggctc

**Protein sequence**

LLQNFAVAICGAKQHWVMSDFIKHEITRIRKLVGDKAQVIGAVSGGVDSTVAARLMQEAIGERFHAVLVDNGVMRLDECQQVQETLQKHLGINLTIVDGSKLFLDRLKGVTEPEKKRSIIGGTFIDLFEEEAIRIEKVAENTPHAGKVEWFLQGTLYPDVIESLSFKGPSATIKTHHNVGGLPA

***Hirsutella sinensis* strain L0106 GMP synthase (guaA-3) mRNA, partial cds.**

**(GenBank accession number, KP090970)**

**Gene sequence**

ctgactgccaaccacctcttctgcaaaatggcccccgtaaacgatgatgccgcgccgcctcacagcactttcgacaccatcctagtcctagactttgggagtcagaccagccatctcatcattcggcgcctcagagccctcaatgtctacgccgagatgctgccctgcaccacccggctccaggacctgacctggtctccggtcggaatcatcctgagcgggggtccctcttccgtatatgatcaaggcgctcctcacgtcgaccccgccgtttttgacttgggcgtgcccattcttggaatctgctatggctgccaggagctcgcttggcgagcagacgctgagaatgtcgctcctggagaggcccgggaatacggccatgcagatgtcgccgttcgcaaggccggcggctccagccacgtcgacaagctctttgaaggcctgggggatataatgcatgtgtacatgagccactttgacaagctcgtcc

**Protein sequence**

LTANHLFCKMAPVNDDAAPPHSTFDTILVLDFGSQTSHLIIRRLRALNVYAEMLPCTTRLQDLTWSPVGIILSGGPSSVYDQGAPHVDPAVFDLGVPILGICYGCQELAWRADAENVAPGEAREYGHADVAVRKAGGSSHVDKLFEGLGDIMHVYMSHFDKLV

***Hirsutella sinensis* strain L0106 guanine deaminase (guaD-1) mRNA, partial cds.**

**(GenBank accession number, KP090971)**

**Gene sequence**

atgaagcccgggaagaaaaactggctcggcttgcaggcaaccacgtccacgtcgccctcgctccagccgaggcgcccgagcagctgctccttggccgccgcgccctcgccgcgccgcgtctcgacgccgacaatcttgccgcccttgtccacgcccacggcgccctcttcaagcacctcgagctgctcgcgcgtcctgctgtgcacaaacgtccccaccagcacctggttcctttcgagcgaccaagcagacgtcattttgcctcctctttcctccgagcttcggtggcttctgcaagagtcggccttgcgtcgcgtccccgagaccc

**Protein sequence**

MKPGKKNWLGLQATTSTSPSLQPRRPSSCSLAAAPSPRRVSTPTILPPLSTPTAPSSSTSSCSRVLLCTNVPTSTWFLSSDQADVILPPLSSELRWLLQESALRRVPET

***Hirsutella sinensis* strain L0106 guanine deaminase (guaD-2) mRNA, partial cds.**

**(GenBank accession number, KP090972)**

**Gene sequence**

ctgcaaaagtacacgttccccctcgaggccagcctcaaggacctggccagggcccgcagcgtctacacggcctgcgtgtgccggacgctggcccacggcaccacgacggccgcctactttgccacgattgacgtggccgccaccaacctgctggccgacctgtgcctcggcctgggccagcgggcctttgtcgggcgcgtgtgcatggacacgcccggcatctgccccgactactatcgtgacgagtcggccgacgagtcgctcagggccacgctcgagaccattgcccacgtcaggcgcatcgacgccagctacgaaatcgtctcgcccatcctgacgccgcgcttcgccccctcgtgctcgggccagtcgatgttgggcctcgccgacgtgcaccgccagcaggggctgcccatccagacgcacatttcggagcacgccgacgacgtcaagctcgcgacggacctgttccccgagtcgggctcgtacgccagaatctacgacgaccacggcctgctgacggccaagacgattctcgcccacgccgtccacctgaccgagggcgaggccacgctcgtgtcggcgcgcggcgcaaaggtggcccactgcccgtgcagcaacgcgtgcctgacgagcggcgaggcccgcgtgcgctggctgtgggaccgcggcatcgacgtgggcctcggcaccgccatgagcggcgggtcacgccccccgcccctcccggcggggggccccgcgggccccccg

**Protein sequence**

LQKYTFPLEASLKDLARARSVYTACVCRTLAHGTTTAAYFATIDVAATNLLADLCLGLGQRAFVGRVCMDTPGICPDYYRDESADESLRATLETIAHVRRIDASYEIVSPILTPRFAPSCSGQSMLGLADVHRQQGLPIQTHISEHADDVKLATDLFPESGSYARIYDDHGLLTAKTILAHAVHLTEGEATLVSARGAKVAHCPCSNACLTSGEARVRWLWDRGIDVGLGTAMSGGSRPPPLPAGGPAGPP

***Hirsutella sinensis* strain L0106 xanthine dehydrogenase/oxidase (XDH-1) mRNA, complete cds.**

**(GenBank accession number, KP090973)**

**Gene sequence**

atggcgcccgtcgcccttctccccgagcgcaacgctcagccggcttccctggcctcgctgacggccgactttgacgacaccgtcagcttctacctcaacggcacaaaggtggtcctcgacgacgtagaccccgacgtcaccgtgctcgagtacctccgcggcatcggcttgacgggcaccaaactcggctgcggcgaaggcggatgcggcgcctgcaccattgtcgtgagccagtacaatcccacctcgaagcaaatataccatgccagcatcaatgcctgtctggcgcccctggccagcctcgacggcaagcacgtcatcaccatcgagggcatcggcaacactcaacgacctcatcccgcccaggagcgcctcgcaaaggggaacggcagccagtgcggcttttgcacccccggcatcgtcatgagcctgtacgccctgctgcgcaacaactcggatccctcccacgacgacatcgaggaggcctttgacggcaacctgtgccgctgcaccggctataggcccatcttggacgcggcccagacctttagtggtgccgacaagtctgcgcagtctcggtctacaggccaatgcgatggcagccccgagaatgtcggctgctgcatgtccaacggcaacggccccgtcgacggcgtctgctgcatgcggaagaagacggcgctggatgaccagcccatcaagcgcttcaccccccctggattcatcgagtacaattccgacacccaactcatcttcccccctgccctcaagaagcacgagatgcggccgctggcctttggcaacaagaggaagaagtggtaccggcccgtcaccctcgaccagctcctccagatcaagagcgtgcacccccaggccaagatcatcggcggcagcaccgagacgcaaatcgaaatcaagttcaaggcgctgcagtaccccgtctccgtgtacgcgggtgacattcccgagctgcgccagtttgccatgcatgacgaccacgtcgaggttggcggaaacgtcgtcttgaccgacctggaggacatttgcgcaaaggcaatcaagcgctacggccacgagagaggccaggtctttgagggcatcttgaagcagctcaagttctttgccgggaggcagatacgcaacgtcggcacgcccgccgggaacctcgccaccgcgtcacccatttcggacttgaaccccatcttgtgggcggcaaacgctgtgctggtggccaagtcggccaccaacgagactgaaatccccatggaccagttctttacgggttatcgtaaaaccgccctccctcaagacgccgtcattgcgtccatcaggatacccgtcactgctcagaagggagagtttctccgcgcgtacaagcaggcgaagcgcaaggacgacgatattgccattgtcacaggggcgctgagggtcaagctggatgacgcgggcgtcgtcaccgactgcaacttgatctacggcggcatggcggcaatgaccgtgtcggccaagacggcgacccaatatctcattggcagacggttcgccgagctcgagacgctcgaaggagccatgagcgccctgggggccgactttgacctgcagttcagcgttcccggcggcatggcctcgtaccgaaaggcgctggcgctcggcttcttctatcgcttttaccatgacgtactcaccaacctggatggaaagtgcggccatgtcgacaaggaggccattgacgaggttgagcgctgcatctccaagggatcagtcgacgaggctgcctctgtggcttacgaaaaggagataacgggcaaatccaacacccacctggccgcgctcaagcagacgacgggcgaggcccagtataccgacgacattcctcctctgaagaacgagctacatggctgctgggtcatgtcgaccaagccccatgcgagaataaattcgattgactactctgtagcgctggatatgcccggcgtggttgactacgtcgacaagaacgacatgccctctgccgaggccaacaagtttggggcaccgcactttgacgaggtcttcttcgcagaaggcgtggtgcacacagccggccagcccatcgccatgatcctggccacgtcggcgtccaaggcgcaggaggcggccagggccgtcaaggtcgagtacgaagagcttcctgcggtgcttacaatggaagaagccgtggagcaggagagctttcaccccttttaccgcgaaatcaagcggggagacaccgaggcggccttcaagagctgtgaccacgtctttaccggcacggtcagaatgggcgggcaggaacacttttacctcgagacgaatgcctgcctggtcattccgaagcccgaggacggagaaatggaagttttcgccagcacgcaaaatgccaacgaaacacaagtattcgtgtcgcgagtgtgcgaggtgcaggcaaacaaggttgtcgtccgtgtcaagagactcggcggcgggttcggcggcaaggaaacgagatccgtggtgctcagctcggcggcggcgctggcagccaagaagacaaaacggccgatacgctgcatgttgacgcgcgaagaggacatgattgccacgggccagcggcaccccttcctcgcgaggtacaaggtcggcgtcaacaaggatggcaagctgcaggcgctggacctcgacgtcttcaacaatgccggctggacgtttgatctcagcgccagcgtcaacgagcgcgccatgacgcacagcgacggctgctacgacatccccaacgtctttgttcgaggccgcgtctgccggacaaacaccatgtccaacacggcttttcgcggcttcggcgggccgcagggcatgttcatcgccgagacgtacatggaggagacggcggaccggctgggcatggccgcggagacgcttcgagagatcaacctttacaagccgcgcggcgtcacgcactttaaccagacgctcgaggactggcacgtccccctcatgtaccagcaggtgcaagacgagtcgtcgtatcccgaacggcgcatcatggtggacaggttcaaccaagagtacaagtggcgcaagaggggcctggcattgatcccgacaaagtttggcatctcgtttacggccctgttcctcaaccaggccggggcgctggtgcacatctaccacgacgggtccgtcctggtggcgcacggcggcacggagatgggccagggcctgcacacgaagctggcgcagattacggcccaggcgctctcggtgccgctggacaacgtcttcatctcggagacggccaccaacacggttgccaacgcgtcggcgacggcggcgtcggcgtcgtcggacctcaacggctacgccatctacaacgcctgcgagcagctcaacgagcggctcgcacggagcggctcgcgccgtaccgcgagaagctgggggccggggcgacgatga

**Protein sequence**

MAPVALLPERNAQPASLASLTADFDDTVSFYLNGTKVVLDDVDPDVTVLEYLRGIGLTGTKLGCGEGGCGACTIVVSQYNPTSKQIYHASINACLAPLASLDGKHVITIEGIGNTQRPHPAQERLAKGNGSQCGFCTPGIVMSLYALLRNNSDPSHDDIEEAFDGNLCRCTGYRPILDAAQTFSGADKSAQSRSTGQCDGSPENVGCCMSNGNGPVDGVCCMRKKTALDDQPIKRFTPPGFIEYNSDTQLIFPPALKKHEMRPLAFGNKRKKWYRPVTLDQLLQIKSVHPQAKIIGGSTETQIEIKFKALQYPVSVYAGDIPELRQFAMHDDHVEVGGNVVLTDLEDICAKAIKRYGHERGQVFEGILKQLKFFAGRQIRNVGTPAGNLATASPISDLNPILWAANAVLVAKSATNETEIPMDQFFTGYRKTALPQDAVIASIRIPVTAQKGEFLRAYKQAKRKDDDIAIVTGALRVKLDDAGVVTDCNLIYGGMAAMTVSAKTATQYLIGRRFAELETLEGAMSALGADFDLQFSVPGGMASYRKALALGFFYRFYHDVLTNLDGKCGHVDKEAIDEVERCISKGSVDEAASVAYEKEITGKSNTHLAALKQTTGEAQYTDDIPPLKNELHGCWVMSTKPHARINSIDYSVALDMPGVVDYVDKNDMPSAEANKFGAPHFDEVFFAEGVVHTAGQPIAMILATSASKAQEAARAVKVEYEELPAVLTMEEAVEQESFHPFYREIKRGDTEAAFKSCDHVFTGTVRMGGQEHFYLETNACLVIPKPEDGEMEVFASTQNANETQVFVSRVCEVQANKVVVRVKRLGGGFGGKETRSVVLSSAAALAAKKTKRPIRCMLTREEDMIATGQRHPFLARYKVGVNKDGKLQALDLDVFNNAGWTFDLSASVNERAMTHSDGCYDIPNVFVRGRVCRTNTMSNTAFRGFGGPQGMFIAETYMEETADRLGMAAETLREINLYKPRGVTHFNQTLEDWHVPLMYQQVQDESSYPERRIMVDRFNQEYKWRKRGLALIPTKFGISFTALFLNQAGALVHIYHDGSVLVAHGGTEMGQGLHTKLAQITAQALSVPLDNVFISETATNTVANASATAASASSDLNGYAIYNACEQLNERLARSGSRRTARSWGPGRR
